# Structures of DPAGT1 Explain Glycosylation Disease Mechanisms and Advance TB Antibiotic Design

**DOI:** 10.1016/j.cell.2018.10.037

**Published:** 2018-11-01

**Authors:** Yin Yao Dong, Hua Wang, Ashley C.W. Pike, Stephen A. Cochrane, Sadra Hamedzadeh, Filip J. Wyszyński, Simon R. Bushell, Sylvain F. Royer, David A. Widdick, Andaleeb Sajid, Helena I. Boshoff, Yumi Park, Ricardo Lucas, Wei-Min Liu, Seung Seo Lee, Takuya Machida, Leanne Minall, Shahid Mehmood, Katsiaryna Belaya, Wei-Wei Liu, Amy Chu, Leela Shrestha, Shubhashish M.M. Mukhopadhyay, Claire Strain-Damerell, Rod Chalk, Nicola A. Burgess-Brown, Mervyn J. Bibb, Clifton E. Barry III, Carol V. Robinson, David Beeson, Benjamin G. Davis, Elisabeth P. Carpenter

**Affiliations:** 1 Structural Genomics Consortium, University of Oxford, Oxford, OX3 7DQ, UK; 2 Chemistry Research Laboratory, University of Oxford, Oxford, OX1 3TA, UK; 3 School of Chemistry and Chemical Engineering, Queen's University, Belfast, UK; 4 Department of Molecular Microbiology, John Innes Centre, Norwich, NR4 7UH, UK; 5 Tuberculosis Research Section, Laboratory of Clinical Immunology and Microbiology, National Institute of Allergy and Infectious Diseases, National Institutes of Health, Bethesda, Maryland 20892, USA; 6 Department of Chemistry, Oxford, OX1 3QZ, UK; 7 Neurosciences Group, Nuffield Department of Clinical Neuroscience, Weatherall Institute of Molecular Medicine, University of Oxford, Oxford, OX3 9DS, UK

**Keywords:** DPAGT1, GPT, Protein N-glycosylation, congenital myasthenic syndrome, congenital disorders of glycosylation, tunicamycin

## Abstract

Protein N-glycosylation is a widespread post-translational modification. The first committed step in this process is catalysed by dolichyl-phosphate N-acetylglucosamine-phosphotransferase DPAGT1 (GPT/E.C. 2.7.8.15). Missense DPAGT1 variants cause congenital myasthenic syndrome and disorders of glycosylation. In addition, naturally-occurring bactericidal nucleoside analogues such as tunicamycin are toxic to eukaryotes due to DPAGT1 inhibition, preventing their clinical use. Our structures of DPAGT1 with the substrate UDP-GlcNAc and tunicamycin reveal substrate binding modes, suggest a mechanism of catalysis, provide an understanding of how mutations modulate activity (thus causing disease) and allow design of non-toxic “lipid-altered” tunicamycins. The structure-tuned activity of these analogues against several bacterial targets allowed the design of potent antibiotics for *Mycobacterium tuberculosis*, enabling treatment *in vitro*, *in cellulo* and *in vivo,* providing a promising new class of antimicrobial drug.

## Highlights

• Structures of DPAGT1 with UDP-GlcNAc and tunicamycin reveal mechanisms of catalysis
• DPAGT1 mutations in patients with glycosylation disorders modulate DPAGT1 activity
• Structures, kinetics and biosynthesis reveal role of lipid in tunicamycin
• Lipid-altered, tunicamycin analogues give non-toxic antibiotics against TB

## Graphical Abstract

**Figure:**
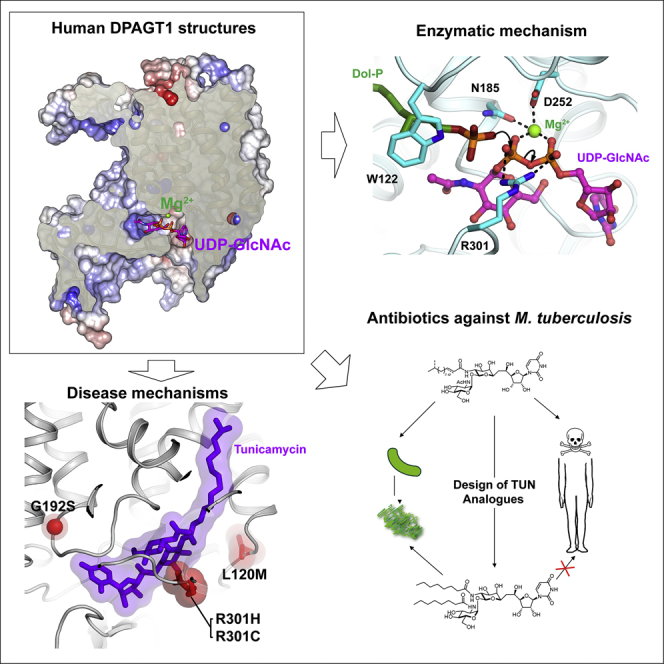

## Teaser

Structural insights into tunicamycin’s toxic interactions with the human N-linked glycosylation pathway allows the identification of non-toxic antibiotics effective against tuberculosis in mice

## Introduction

**Figure 1:**
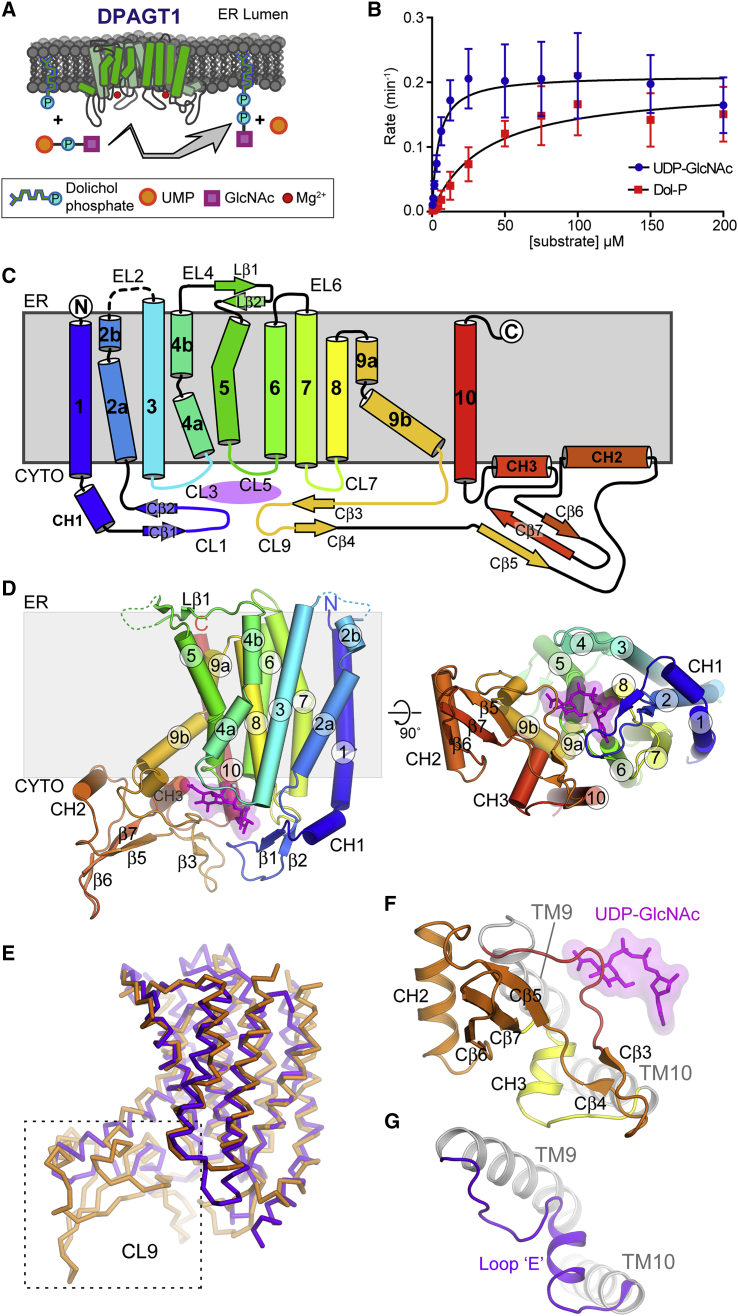
Structural Features of DPAGT1 (A) Cartoon of DPAGT1 reaction. (B) Michaelis-Menten kinetics, non-titrated substrate at 200 μM. Measurements were performed with three biological and three technical repeats. Data presented are means ± SD. (C) Topology of DPAGT1; helices shown as cylinders, strands as arrows and active site in magenta. (D) Schematic of DPAGT1 structure. Views shown looking along membrane plane and onto the cytoplasmic face; UDP-GlcNAc in magenta. (E) Comparison of DPAGT1 (orange) and MraY (PDB: 5CKR; purple) folds. (F) CL9 domain in DPAGT1. (G) CL9 strand (loop “E”) and helix in MraY.

N-glycosylation of asparagine residues is a common post-translational modification of eukaryotic proteins, required for protein stability, processing, and function. Many diseases are associated with incorrect glycosylation (Freeze et al., 2012). This process requires dolichol-PP-oligosaccharides that provide the oligosaccharides that are transferred (Helenius and Aebi, 2004). The first step in dolichol-PP-oligosaccharide production involves the ER integral membrane enzyme dolichyl-phosphate alpha-N-acetyl-glucosaminyl-phosphotransferase (DPAGT1, E.C. 2.7.8.15, also known as GlcNAc-1-P Transferase [GPT]). It catalyzes the transfer of an N-acetyl-D-glucosamine-1-phosphoryl unit (GlcNAc-1-P) from UDP-N-acetyl glucosamine (UDP-GlcNAc) onto dolichyl phosphate (Dol-P) (Figure 1A) (Heifetz and Elbein, 1977, Lehrman, 1991). The product GlcNAc-PP-Dol is anchored to the ER membrane by its dolichyl moiety and then monosaccharide units are added sequentially to build the N-glycan that is then transferred.

Mutations in DPAGT1 impair protein N-glycosylation, leading to at least two syndromes, depending on the extent of loss of activity. Congenital myasthenic syndrome (DPAGT1-CMS OMIM ref: 614750) is a neuromuscular transmission disorder characterized by fatigable weakness of proximal muscles (Basiri et al., 2013, Belaya et al., 2012, Iqbal et al., 2013). Reduced endplate acetylcholine receptors (AChR) and abnormal synaptic structure may be the result of incorrect glycosylation of AChR and other proteins. Mutations in DPAGT1 also cause congenital disorder of glycosylation type Ij (CDG-Ij, OMIM ref: 608093) (Carrera et al., 2012, Selcen et al., 2014, Wu et al., 2003, Würde et al., 2012), a more severe multisystem syndrome that can cause intellectual disability, epilepsy, microcephaly, severe hypotonia and structural brain anomalies.

**Figure S1:**
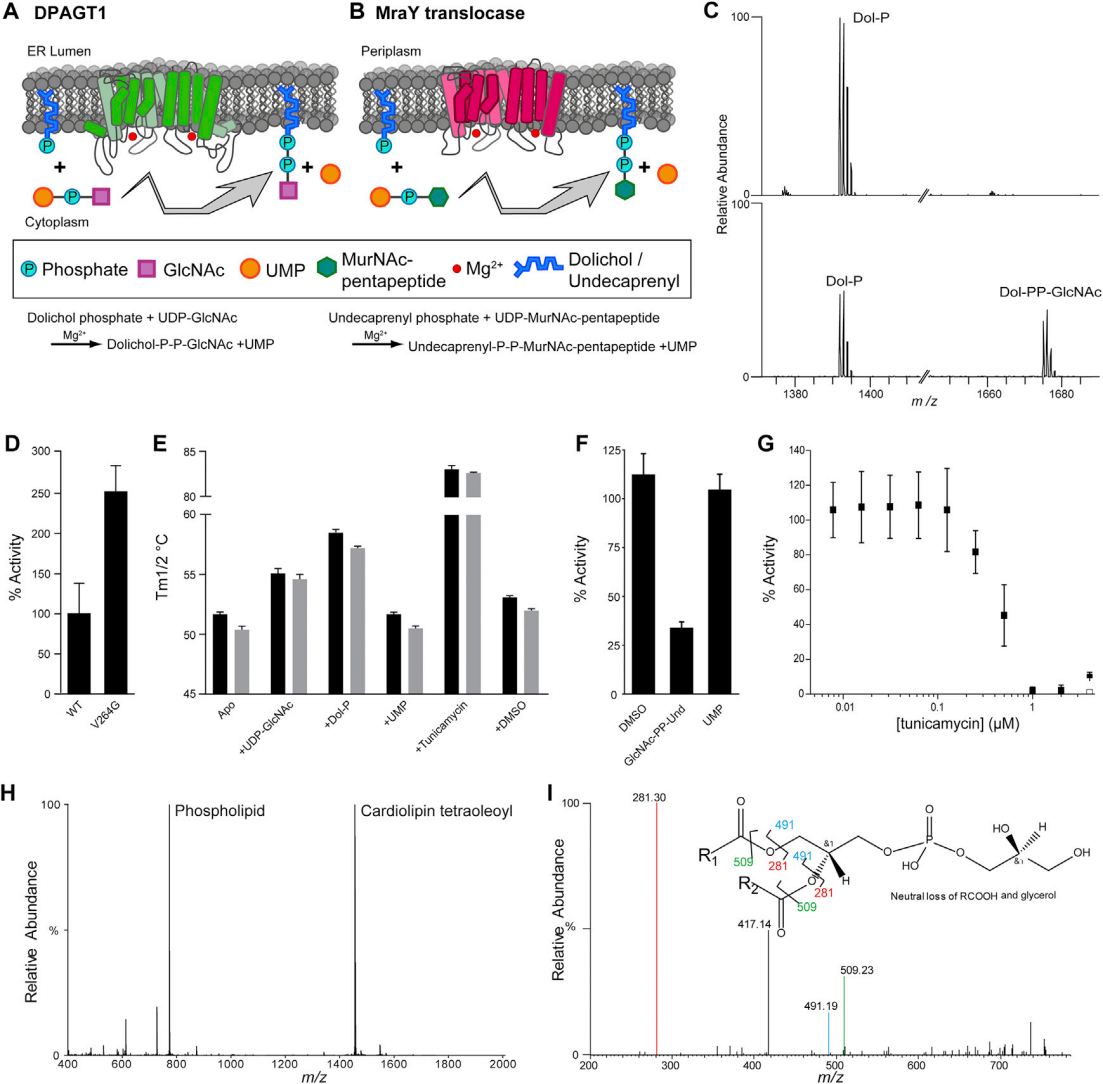
Biochemical and Biophysical Characterization of DPAGT1, Related to Figure 1 (A) Cartoon of DPAGT1 showing the reaction it performs. (B) Cartoon of MraY showing the reaction it performs. (C) The identity of the substrate Dol-P and the product, GlcNAc-PP-Dol, was confirmed by mass spectrometry. Top spectrum is DPAGT1 incubated with Dol-P only, bottom spectra are DPAGT1 incubated with both Dol-P and UDP-GlcNAc. (D) Comparison of the catalytic activity of DPAGT1 WT and Val264Gly mutant protein (n=9). (E) The thermostability of DPAGT1 WT (black) and Val264Gly (grey) mutant proteins tested using label free differential scanning fluorimetry. The effects of addition of the substrates Dol-P and UDP-GlcNAc, and the inhibitor tunicamycin on thermostability of DPAGT1 were also tested (n=9). (F) Product inhibition was observed with the product analogue GlcNAc-PP-Und, but not with UMP (n=9). (G) DPAGT1 is completely inhibited by a 1:1 ratio of tunicamycin:protein (n=9). (H) Lipidomics analysis of OGNG purified DPAGT1 showed the presence of co-purified phospholipid in addition to the supplemented cardiolipin associated with the protein. (I) The presence of phosphatidylglycerol is confirmed by tandem mass spectrum of the most intense phospholipid in the lipidomics analysis. For all panels, data presented are means ± SD.

Inhibition of polyisoprenyl-phosphate N-acetylaminosugar-1-phosphoryl transferases (PNPTs) such as DPAGT1 and the bacterial enzyme MraY, is lethally toxic to many prokaryotic and eukaryotic organisms. *Streptomyces* bacteria exploit this toxicity by producing the PNPT inhibitor tunicamycin, which blocks MraY, a critical enzyme in biosynthesis of cell walls in many bacterial pathogens (Figures S1A and S1B) (Dini, 2005). Unfortunately, it also inhibits eukaryotic PNPTs, such as DPAGT1 (Heifetz et al., 1979) causing severe toxicity in eukaryotic cells. However, although bacterial (e.g., MraY) and human (e.g., DPAGT1) PNPTs are similar, it should be possible to design synthetically-altered tunicamycin analogues that specifically inhibit bacterial proteins.

Here we present structures of human DPAGT1 with and without ligands. The protein production methods, structures, assays and complexes are components of a “target enabling package” developed at the Structural Genomics Consortium (released June 2017, http://www.thesgc.org/tep/DPAGT1), which has already been used by others (Yoo et al., 2018). These structures, combined with mutagenesis and activity analysis, reveal both the mechanism of catalysis by DPAGT1 and the molecular basis of DPAGT1-related diseases. To improve the effectiveness of tunicamycin as a drug, we modified its core scaffold, **TUN**, using a scalable, semi-synthetic strategy that enabled selective lipid chain addition. These analogues show nanomolar antimicrobial potency, ablated inhibition of DPAGT1, much reduced toxicity, allowed effective treatment of *Mycobacterium tuberculosis* (*Mtb*) in mammals, providing leads for tuberculosis (TB) antibiotic development.

## Results

### DPAGT1 Activity and Architecture

To determine the structure of DPAGT1, we expressed both full-length wild-type (WT) DPAGT1 and the missense mutant Val264Gly in the baculovirus/insect cell system. The Val264Gly mutation is a variant found in some CMS patients (Belaya et al., 2012), which improved crystallization behavior compared to WT. We tested the enzymatic activity of WT and Val264Gly DPAGT1 to confirm that the protein was functional. Product GlcNAc-PP-Dol was confirmed by mass spectrometry (Figure S1C). WT DPAGT1 has an apparent *K*_m_ of 4.5 ± 0.8 μM and a k_cat_ of 0.21 ± 0.007 min^-1^ towards UDP-GlcNAc (Figure 1B, STAR Methods); Dol-P displayed an apparent *K*_m_ of 36.3 ± 7.2 μM and a k_cat_ of 0.20 ± 0.012 min^-1^ (Figure 1B). The Val264Gly mutant showed 2.5-fold higher activity (Figure S1D) and similar thermostability to WT protein (Tm_1/2_ of 51.7 ± 0.2 °C for WT and 50.4 ± 0.3 °C for mutant, Figure S1E, STAR Methods, Table S1). Adding the product analogue GlcNAc-PP-Und (equimolar to Dol-P and UDP-GlcNAc) reduced activity 3-fold, the addition of the other product, UMP, had no effect (Figure S1F). Tunicamycin fully inhibited at 1:1 molar ratio with DPAGT1 (Figure S1G). While both substrates thermostabilized WT and mutant DPAGT1 by 3–7 °C, tunicamycin thermostabilized both by more than 30 °C (Figure S1E). Interestingly, phosphatidylglycerol co-purified with DPAGT1, even when not added to the purification, suggesting a role for phosphatidylglycerol in the stability of DPAGT1 (Figures S1H and S1I).

**Figure S2:**
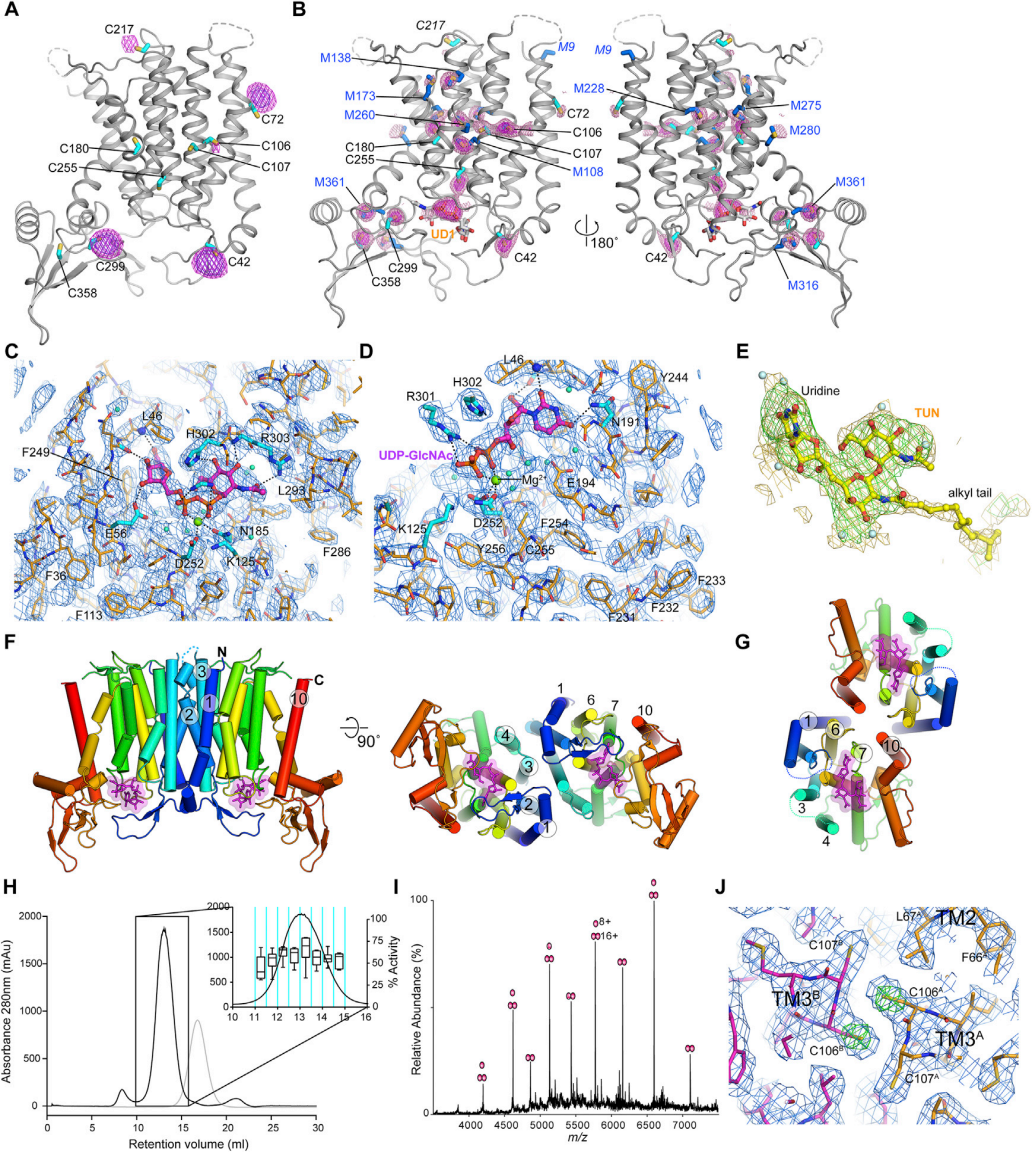
Electron Density Maps, Heavy Atom Phasing and Dimer Interface Analysis for the Structure of Dimeric WT and Gly264Val Mutant of DPAGT1 Solved at Resolutions up to 3.2 Å, Related to Figure 2 (A) Hg bound sites from a soaked crystal. 6 Å anomalous difference Fourier map calculated from a dataset collected from a crystal soaked with EMTS is contoured at 5σ (purple) and 3σ (magenta mesh) and overlaid on the final model. Cysteine positions are highlighted by cyan sticks. Labelling of five of the nine ordered cysteines (Cys42, Cys72, Cys106 (weak), Cys217 and Cys299) is observed under the soaking conditions used. (B) S-SAD peaks. The PHASER-EP log-likelihood map after anomalous model completion with sulphur atoms is shown overlaid on a cartoon representation of the final UDP-GlcNAc complex. The positions of cysteine (cyan sticks) and methionine (blue sticks) residues are highlighted. The map is contoured at 4.5σ (magenta) and 2.5σ (pink mesh). Peaks are observed for 16 sulphur atoms (out of a total of 18 possible ordered sulphurs) and the pyrophosphate of the UDP-GlcNAc is also resolved. (C and D) Two views of final 2F_o_-F_c_ AUTOBUSTER electron density map around the active site in the UDP-GlcNAc complex. The map has been sharpened using a *B*-factor of -100 Å^2^ in COOT and is contoured at 1.5σ and overlaid on the final model. UDP-GlcNAc is shown in ball-and-stick form (carbon-magenta, oxygen-red, phosphorus-orange, nitrogen-blue). (E) Omit F_o_-F_c_ electron density map for tunicamycin. The omit difference density is contoured at 2.5σ (green mesh) and a sharpened omit F_o_-F_c_ density map (*B*=-100 Å^2^) is contoured at 2σ and overlaid on the final tunicamycin coordinates. Density for tunicamycin’s alkyl tail is not as well resolved as the TUN core. (F and G) Comparison of dimer organization in DPAGT1 (F) and unbound MraY (PDB: 5JNQ) (G) in crystals, indicating that DPAGT1 and MraY are both “head-to’head” dimers in their respective crystals, but the dimer interfaces are unrelated. (H) Size exclusion chromatography from a DPAGT1 purification with activity data per fraction per unit of protein shown as box plot, indicating that there is no difference in the activity of DPAGT1 across the peak (n=6). (I) Native mass spectrometry confirms that DPAGT1 is a mixture of monomers and dimers. (J) Electron density at the dimer interface between adjacent Cys106 residues in TMH3, with the sharpened (-100 Å^2^) 2*F*o*F*c density shown in blue (contoured at 1.0sigma) and “omit” density from a refinement where the sidechain of Cys106 was omitted (green mesh, contoured at 6.0sigma).

The crystal structures of the WT DPAGT1 and Val264Gly mutant were solved by X-ray crystallography using molecular replacement with the bacterial homologue MraY (Chung et al., 2013, PDB: 4J72, 19% identity) as an initial model (STAR Methods). The apo WT- and Val264Gly-DPAGT1 proteins gave structures to 3.6 Å and 3.2 Å resolution (Table S2). Complexes with UDP-GlcNAc and tunicamycin gave data to 3.1 Å and 3.4 Å resolution, respectively (Figure 1, Figures S2A–S2E and Table S2). In the crystals DPAGT1 is a dimer formed through a crystallographic 2-fold axis (Figure S2F), with an 1850 Å^2^ interaction surface. The interface observed is similar to that seen in the Yoo et al., 2018 structures, although in that case the crystals show a dimer in the asymmetric unit. Interestingly, comparison with the dimeric bacterial homologue MraY, showed that the surface that forms the dimer interface differs between DPAGT1 and MraY (Figure S2G) (Yoo et al., 2018). In solution, DPAGT1 exists predominantly as a dimer, as shown by native mass spectrometry, although the monomer was also detected (Figures S2H and S2I). A Leu103Phe mutation introduced at the dimer interface to disrupt this interaction gave unstable protein, suggesting that DPAGT1 dimerization plays a role in stability. In the dimer interface the sidechains of Cys106 are adjacent, but no intermolecular disulfide bond was observed in our electron density maps (Figure S2J). To assess the presence of this potential disulfide, DPAGT1 was purified without reducing agents and its mass matched only monomer (STAR Methods). No covalent, disulfide-bonded dimer was observed. We concluded that DPAGT1 exists predominantly as a non-covalent dimer in solution and that dimerization is important for its stability.

The DPAGT1 structure (as reported here and by Yoo et al., 2018) consists of 10 transmembrane helices (TMH1 to 10), with both termini in the ER lumen (Figures 1C and 1D). Five loops connect the TMHs on the cytoplasmic side of the membrane (CL1, -3, -5, -7, and -9), which form the active site, 3 loops on the ER side of the membrane (EL2, -4, -6) and one (EL8) embedded in the membrane on the ER side. DPAGT1 has a similar overall fold to MraY (Chung et al., 2013, Yoo et al., 2018) (Figure 1E). A feature of the eukaryotic DPAGT1 PNPT family not found in prokaryotic PNPTs is a 52-residue insertion between Arg306-Cys358 in CL9, following TMH9. This motif adopts a mixed α/β fold with an extended structure with 2 β-hairpins, a 3-stranded β-sheet (Cβ5-Cβ7) and two amphipathic α-helices (CH2/CH3). This CL9 domain (Figure 1F) forms part of the substrate recognition site in human DPAGT1 but not in bacterial MraY (Figure 1G).

**Figure 2:**
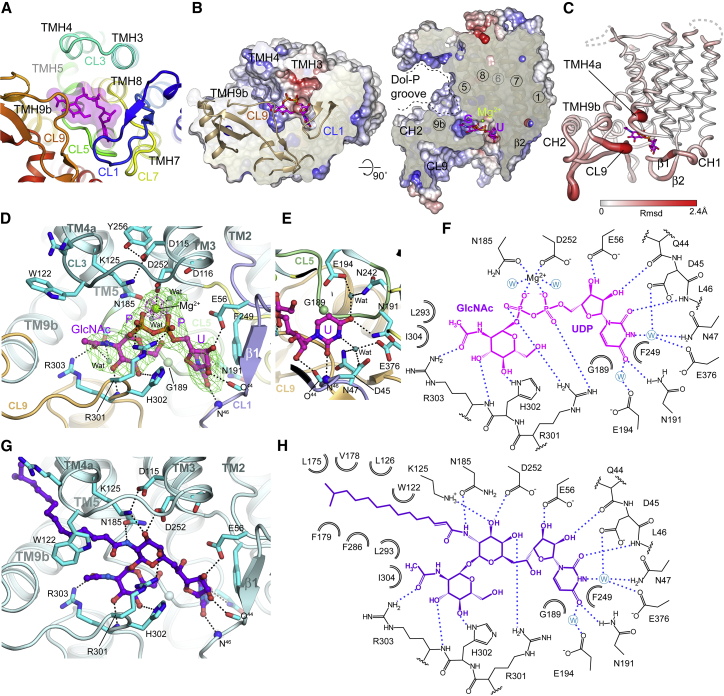
DPAGT1 Active Site (A) The loops that form the active site; UDP-GlcNAc in magenta. (B) Sliced molecular surface showing occluded active site cleft and putative Dol-P recognition groove. Surface is coloured by electrostatic potential; UDP-GlcNAc in magenta. (C) Conformational changes with UDP-GlcNAc binding. Protein depicted in tube form with the tube thickness and colouring reflecting the rmsd in mainchain atomic positions between the unbound and UDP-GlcNAc-bound structures. (D) UDP-GlcNAc binding in active site. Omit Fo-Fc difference electron density shown for UDP-GlcNAc (green mesh, contoured at 3σ) and 4 Å anomalous difference Fourier electron density (magenta mesh, contoured at 15σ) from a dataset with MnCl_2_. (E) Recognition of uridine moiety of UDP-GlcNAc. (F) Schematic representation of interactions made by UDP-GlcNAc. (G) Tunicamycin binding in active site. (H) Schematic representation of interactions made by tunicamycin.

The active site is on the cytoplasmic face of the membrane, formed by the 4 cytoplasmic loops between the TMHs (Figure 2A). The long CL1 loop forms the “back wall”; CL5 and CL7 form the base and the “side walls” are formed by TMH3-CL3-TMH4, TMH9b and the extended loop at the start of the CL9 domain (Ile297-Pro305). The entrance to the catalytic site, between TMH4 and TMH9b, is open and accessible from the lipid bilayer via a 10 Å wide cleft. Within the membrane, adjacent to the active site, there is a hydrophobic concave region (Figure 2B), created by a 60^°^ bend in TMH9 midway through the bilayer, which creates a groove in the DPAGT1 surface (Figure 1D).

### Binding Mode for UDP-GlcNAc and Metal Ion

The structure of the DPAGT1/UDP-GlcNAc complex reveals an overall stabilization of the active site, due to movements of CL1 and CL9, and the N terminus of TMH4 (Figure 2C), without any global changes in conformation. The C-terminal end of TMH9b and the following loop region (Phe286-Ile304) display the largest conformational change with an induced fit around the GlcNAc-PP (Figure 2C).

The uridyl moiety of UDP-GlcNAc lies in a narrow cleft at the back of the active site formed by CL5 and CL7. The uracil ring is sandwiched between Gly189 and Phe249 with additional recognition conferred by hydrogen bonds between the Leu46 backbone amide, the Asn191 sidechain to the uracil carbonyls (Figures 2D and 2E) and an extensive hydrogen bond network involving two waters links the uracil ring to five residues (Figures 2D and 2E). Hydrogen-bonding of the ribosyl hydroxyls to the Gln44 mainchain carbonyl and Glu56 sidechain carboxylate complete the recognition of the uridyl nucleotide.

The pyrophosphate bridge is stabilized by interactions with Arg301 and by the catalytic Mg^2+^ (Figure 2D). The Arg301 sidechain coordinates one pair of α and β phosphate oxygen atoms, whilst the Mg^2+^ ion is chelated by the second pair of α and β phosphate oxygens. Each oxygen atom is thus singly coordinated in an Arg-Mg^2+^-“pyrophosphate pincer.” The octahedral coordination of the Mg^2+^ ion is completed by the sidechains of conserved residues Asn185 and Asp252, and 2 water molecules. Data from DPAGT1 co-crystallized with UDP-GlcNAc and Mn^2+^ gave a single anomalous difference peak at the metal ion binding site, confirming the presence of a single Mg^2+^ ion in the active site (Figure 2D). The position of the Mg^2+^ ion differs by 4 Å from that observed in MraY unliganded structure and the co-ordination differs (Chung et al., 2013).

The GlcNAc moiety-binding site is formed by the CL9 domain and the CL5 loop, although all the direct hydrogen bond interactions are with the CL9 domain. The OH3 and OH4 hydroxyls of GlcNAc form hydrogen bonds with the sidechain of His302 and the mainchain amide of Arg303, respectively. The mainchain of residues 300–303 and the sidechain of Arg303 define the GlcNAc recognition pocket by specifically recognizing the N-acetyl substituent, forming a wall to the sugar-recognition pocket that appears intolerant of larger substituents, thereby “gating” substrate. This structure is absent in MraY, which has a much smaller CL9 loop.

**Figure 3:**
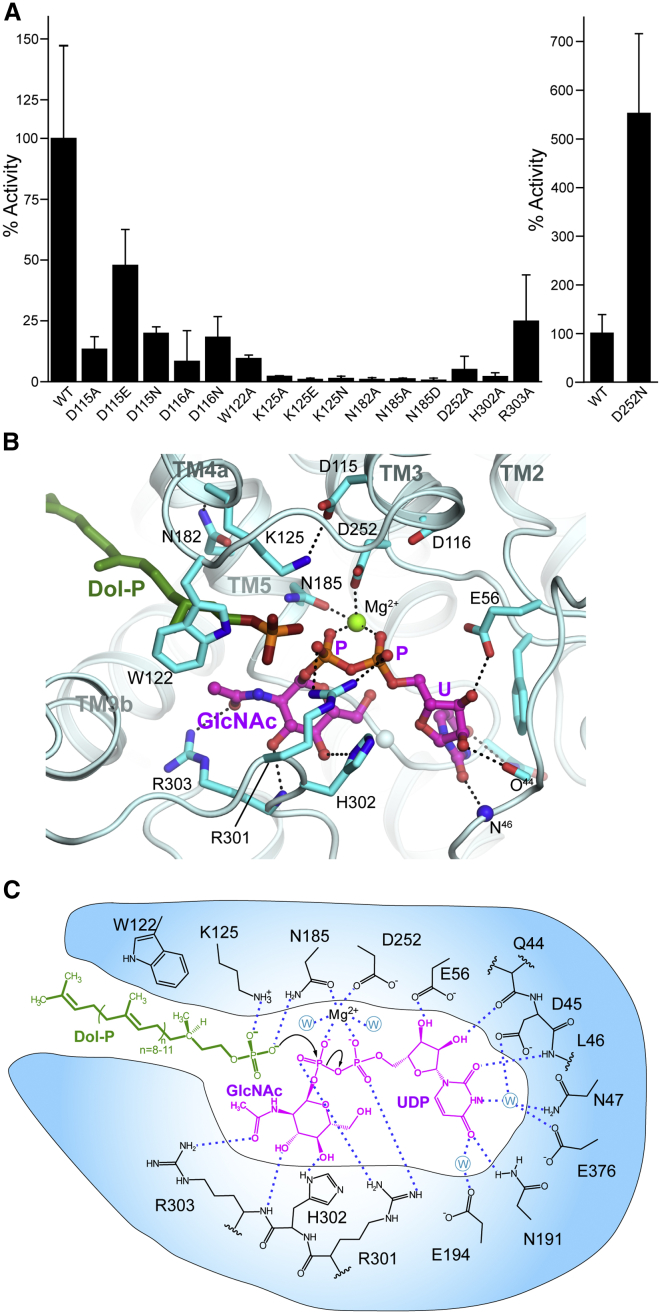
Proposed DPAGT1 Catalytic Mechanism (A) Relative activity of active site mutant residues; (n=9). (B) UDP-GlcNAc complex active site structure with Dol-P modelled based on tunicamycin complex lipid chain position. (C) Proposed DPAGT1 catalytic mechanism. For all panels, data presented are means ± SD.

Surprisingly, this structure does not support prior predictions that the highly conserved “aspartate rich” D^115^Dxx(D/N/E)^119^ motif is directly involved in Mg^2+^ binding and/or catalysis (Lloyd et al., 2004). This sequence is adjacent to the active site, but these residues do not directly coordinate Mg^2+^ or substrate (Figure 2D). Instead, Asp115 is hydrogen-bonded to Lys125 and Tyr256. Lys125 lies adjacent to the phosphates (Figure 2D) and has been implicated in catalysis (Al-Dabbagh et al., 2008). Asp116 forms hydrogen bonds to Ser57 and Thr253 and N-caps TMH8, thus stabilizing residues that interact with the UDP ribosyl moiety (Glu56) and the Mg^2+^ (Asp252). DPAGT1 with residues Asp115 and Asp116 mutated to Asn, Glu, or Ala retained at least 10% of WT activity (Figure 3A), suggesting they are not essential for catalysis. The third residue in this motif, Asn119, makes no significant interactions. Thus, 2 of the 3 conserved residues perform structural roles; none are directly involved in Mg^2+^-binding or catalysis.

### Comparison of the UDP-GlcNAc and Tunicamycin Complexes

The structure of the complex between tunicamycin and DPAGT1 (this work and Yoo et al., 2018) suggests that inhibition is achieved through partial mimicry of the Michaelis complex formed during catalysis with acceptor phospholipid Dol-P and UDP-GlcNAc. The uridyl and GlcNAc moieties in tunicamycin and UDP-GlcNAc occupy essentially identical sites (Figures 2G and 2H). In tunicamycin the pyrophosphate of UDP-GlcNAc is replaced by a galactosaminyl moiety, which displaces the Mg^2+^ and interacts with the sidechains of Arg301, Asp252, and Asn185.

Tunicamycin’s mimicry of Dol-P also gave critical insight into the potential binding of co-substrate Dol-P. The lipid chain of tunicamycin occupies the concave groove that runs along TMH5, between helices TMH4 and TMH9a (see above). The sidechain of Trp122 pivots around its Cβ–Cγ bond to lie over the lipid chain, trapping it in a tunnel. The surface beyond this hydrophobic tunnel, up to the EL4 loop on the ER lumen face of the membrane is highly conserved, so it is likely that the Dol-P lipid moiety could bind to this surface. At the other end of the tunicamycin lipid moiety, the amide forms polar interactions with Asn185 and lies close to Lys125, suggesting that the amide moiety partially mimics the phosphate head-group of Dol-P (Figures 2G and 2H).

### The DPAGT1 Catalytic Mechanism

Alternative mechanisms have been proposed for the PNPT family including one-step, nucleophilic attack (Al-Dabbagh et al., 2008) or two-step, double displacement via a covalent intermediate (Lloyd et al., 2004). We did not observe any covalent modification of DPAGT1 in the presence of UDP-GlcNAc, nor did we see release of UMP in the absence of Dol-P as might be predicted for a two-step mechanism. Also, the active site lacks suitably placed residues that could act as a nucleophile. Therefore the most probable mechanism involves direct nucleophilic attack by Dol-P phosphate oxygen atom on the phosphorus atom of the β-phosphate of UDP-GlcNAc, causing phosphate inversion and loss of UMP (Figures 3B and 3C).

When bound to DPAGT1, UDP-GlcNAc adopts a bent-back conformation, with the donor sugar lying below the phosphates, rotated towards the uridine (Figure 2D). The pyranose ring is inclined so that the O6 hydroxyl of the GlcNAc is within 3.1 Å of the O5B atom of the α-phosphate. This orientation presents the β-phosphate of the UDP-GlcNAc to the position that would be occupied by the phosphate of the Dol-P, exposing the lowest unoccupied molecular orbital (LUMO) of its β-phosphate electrophile for reaction with the Dol-P phosphate *O*-nucleophile (Figures 3B and 3C).

Providing the correct geometry for this one-step phosphoryl transfer appears to be key to catalysis. Analyses of other enzymatic phosphoryl transfer reactions suggest that a bridging Arg (in a very similar position to Arg301) and bridging metals (in a very similar position to the Mg^2+^ ion) do not tighten the transition state but instead provide binding energy that optimizes geometry and alignment for attack (Lassila et al., 2011). They may also preferentially favor the formation of trigonal bipyramidal geometry during nucleophilic attack. Similarly, despite classical emphasis on reducing electrostatic repulsions between anionic nucleophiles with anionic electrophiles, such as those present in phosphoryl transfer (Westheimer, 1987), such effects are small in model systems (Lassila et al., 2011). This suggests that the role of Lys125, which would be close to the phosphate oxygens in Dol-P, would be mainly to guide the phosphate into position. The correct alignment of Dol-P for attack would be further facilitated by the “grip” provided by Trp122 holding the Dol-chain into the tunnel observed in the tunicamycin⋅DPAGT1 complex.

Representative residues proposed to bind Dol-P, sugar and pyrophosphate were probed by mutagenesis. Mutation of Mg^2+^-chelating residues to Ala (Asn185Ala and Asp252Ala) reduced DPAGT1 activity to 1.2% and 7%, respectively (Figure 3A). A conservative Asn185Asp mutation, expected to retain Mg^2+^-binding activity, also ablated activity (0.7% of WT) suggesting an additional role for Asn185 in catalysis. The amide group of Asn185 lies within 4 Å of the predicted Dol-P phosphate-binding site, forming hydrogen bonds with the nucleophilic oxygen of Dol-P to guide it towards the β-phosphate. Lys125 Mutations (Lys125Ala, Lys125Glu, and Lys125Gln) near the Dol-P phosphate binding site, all reduced the activity to below 2.2%, consistent with it playing a critical guiding role. Interestingly, an Asp252Asn mutation increased activity 5-fold (Figure 3A). This mutation removes a coordinating negative charge from the Mg^2+^, making it more electropositive and the β-phosphorus more electrophilic, potentially increasing its susceptibility to nucleophilic attack.

Mutation of His302, which hydrogen bonds to the O4 oxygen of GlcNAc in UDP-GlcNAc to hold it in its bent-back conformation, caused 98% loss of activity, consistent with it playing a role in aligning the nucleophile to attach the β-phosphate. Finally, mutations of Arg301, found in patients with CDG-Ij (Imtiaz et al., 2012), that is part of the “pyrophosphate pincer” caused ∼95% loss of activity.

### Mutations in DPAGT1 in CMS and CDG

**Figure 4:**
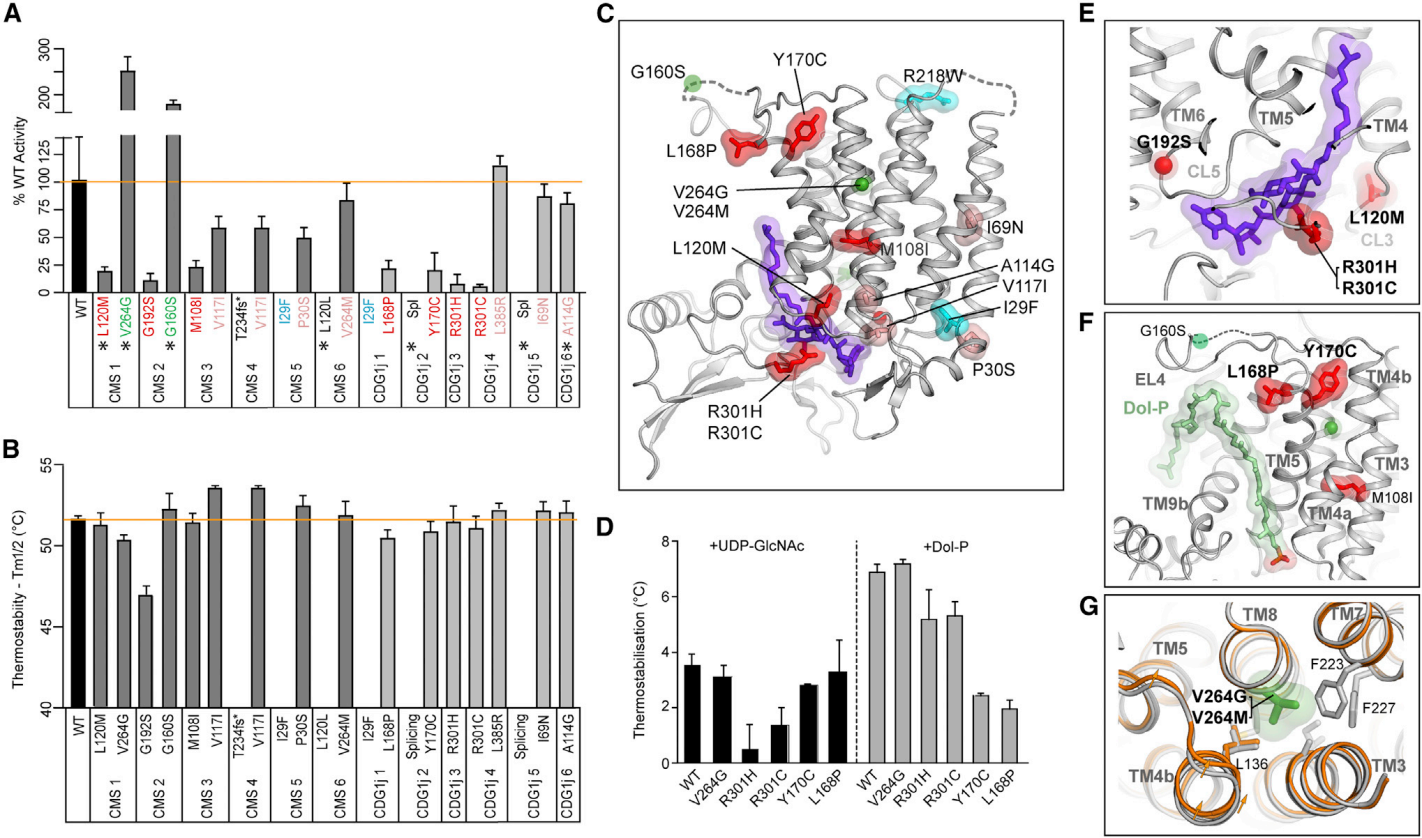
DPAGT1 CMS and CDG-Ij Missense Variants Analyses (A) Relative activity and (B) thermostability for DPAGT1 missense variants found in CMS (dark grey) or CDG-Ij (mid grey). WT activity/stability is indicated by an orange line. Mutations are colored in panels A, D, E-G as follows: 25% or less activity: red; 50%–100% WT activity: pink; >WT activity: green; instability on purification: cyan; confirmed splicing variants indicated with a ^∗^; Spl: unknown splicing variant. For all measurements n=9. (C) Location of variants mapped onto tunicamycin (purple) complex, colored as above. (D) Changes in thermostabilization of selected variants by substrates UDP-GlcNAc and Dol-P (n=9). (E) Location of variants near UDP-GlcNAc binding site. (F) Variants near predicted Dol-P binding site. Dol-P (pale green) has been modelled based on the tunicamycin lipid tail. (G) Environment of Val264. Structures of WT (grey) and V264G variant (orange) are shown illustrating the subtle conformational difference in TM4b and EL4. For all panels data presented are means ± SD.

**Figure S3:**
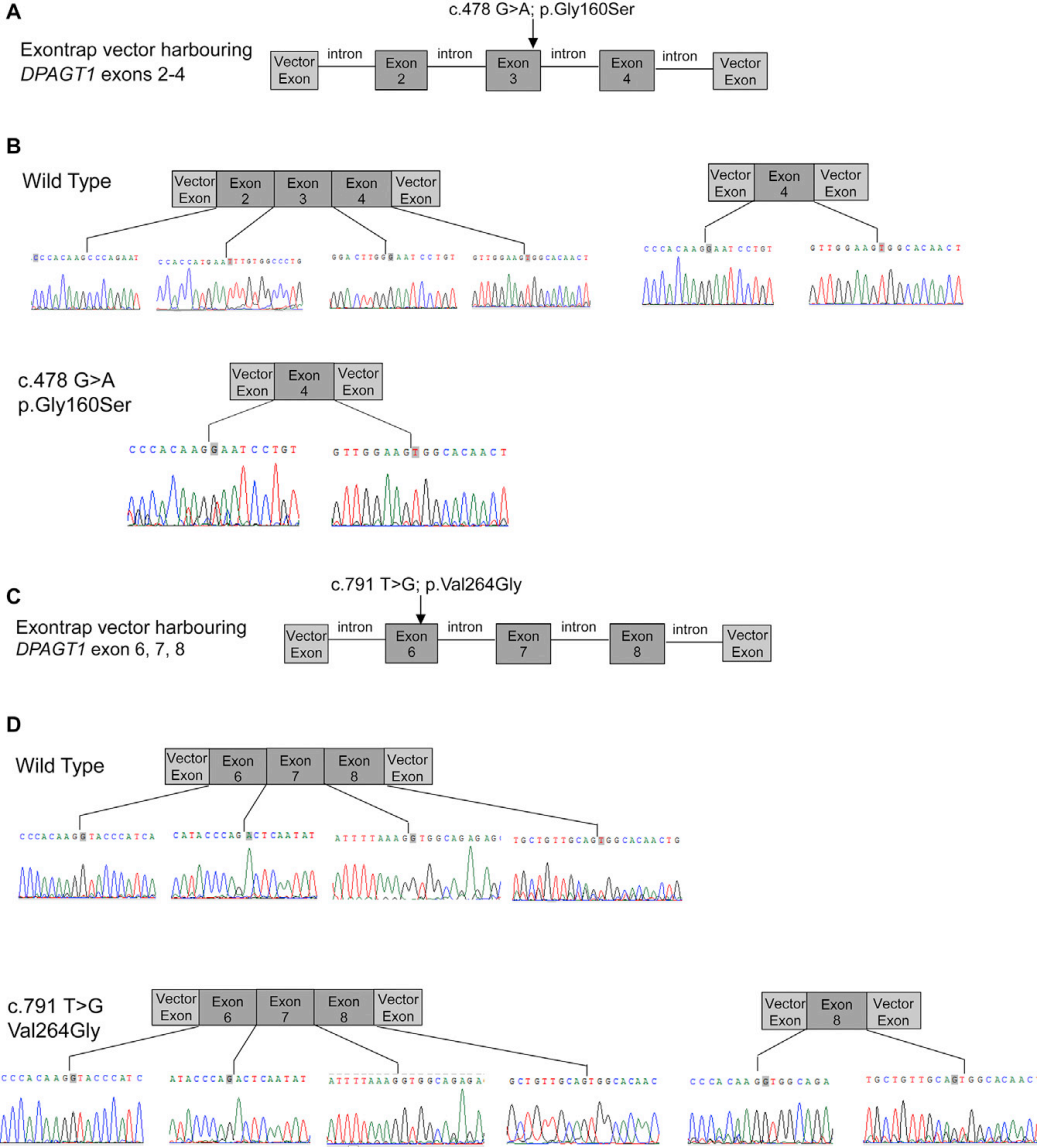
The c.478G>A; Gly160Ser and c.791T>G; Val264Gly Mutations Are Associated with Exon Splicing Errors, Resulting in the Loss of Exons 2 and 3 from the Transcript Harbouring c.478G>A; Gly160Ser and the Loss of Exons 6 and 7 from the Transcript Harbouring c.791T>G; Val264Gly, Related to Figure 4 (A) Schematic of pET01 exon trap vector with DPAGT1 exons 2-4 inserted showing the location within the genomic sequence of c.478G>A; Gly160Ser. (B) Sequencing data and schematic diagrams showing aberrant splicing that results from genomic sequence harbouring the c.478G>A; Gly160Ser variant. RT-PCR on RNA produced in TE671 muscle cell line following transfection with the “exon trap” vector gave wild type RNA sequence, but also some transcripts that excluded exons 2 and 3. When the genomic sequence contained the c.478G>A; Gly160Ser variant was transfected only RNA missing exons 2 and 3 was detected. (C) Schematic of pET01 exon trap vector with DPAGT1 exons 6-8 inserted showing the location within the genomic sequence of the c.791T>G; Val264Gly variant. (D) Sequencing data and schematic diagrams showing sequences obtained following RT-PCR on TE671 cells transfected with the “exon trap” vector containing human genomic DNA that is either wild type or has the c.791T>G; Val264Gly variant. Wild type sequence generated RNA harbouring only exons 6, 7 and 8, as shown. The c.791T>G; Val264Gly variant generated some RNA transcripts containing exons 6, 7 and 8 (as for wild type) but for the majority of transcripts exons 6 and 7 were excluded and only exon 8 was present.

DPAGT1-CMS and CDG-Ij are recessive disorders, caused by loss of DPAGT1 function (Table S1), through loss of enzymatic activity, protein truncation or RNA splicing defects. Structures, catalytic activity (Figure 4A), and thermostability measurements (Figure 4B) for purified mutant proteins allowed us to examine how DPAGT1 variants cause disease (Figures 4 and S3, Table S1). Surprisingly, in most cases mutated DPAGT1 protein had close to WT thermostability (Figure 4B), suggesting correct folding. Only two (Ile29Phe and Arg218Trp, Basiri et al., 2013, Iqbal et al., 2013) gave protein that was too unstable to purify, due to insertion of large hydrophobic sidechains either at the ER surface or in the core of the protein (Figure 4C).

Many CMS patients have compound heterozygous mutations, and in general one allele had a much greater impact on DPAGT1 function, with either loss of at least 75% of the WT catalytic activity (Leu120Met, Gly192Ser, Met108Ile, Figure 4A, Table S1) or protein quantity (e.g., truncation: Thr234Hisfs^∗^116, (Belaya et al., 2012); low protein yield: Ile29Phe (Klein et al., 2015); exon skipping: Leu120Leu (Selcen et al., 2014). However, expressed protein from the second allele had 50% or more WT catalytic activity (Val117Ile, Pro30Ser, Val264Met) or even increased activity (Val264Gly and Gly160Ser, Belaya et al., 2012) (Figure 4B, Table S1). The only homozygous CMS mutation, Arg218Trp (Basiri et al., 2013), gave protein that was too unstable to purify. Patients with the more severe CDG-Ij disease often had significant loss of function for both alleles, with less than 25% activity (Leu168Pro, Tyr170Cys, Arg301His, Arg301Cys), low protein yield/stability (Ile29Phe), or disrupted splicing (with WT activity and thermostability for the purified protein, e.g., Ala114Gly, Würde et al., 2012). The Leu385Arg (Carrera et al., 2012) and Ile69Asn (Timal et al., 2012) mutations are unusual in that they did not show loss of stability or activity, suggesting that splicing changes should be investigated.

As expected, mutations with less than 25% activity generally lie in or near the substrate-binding regions. In two variants found in patients with CDG-Ij (Arg301His and Arg301Cys, Carrera et al., 2012, Imtiaz et al., 2012), the Arg301 that “pincers” the UDP-GlcNAc pyrophosphates is mutated (Figures 2D and 2F) and these mutations gave reduced thermostabilization by UDP-GlcNAc (Figure 4D), confirming a role in UDP-GlcNAc binding. Similarly, the CMS variant Leu120Met lies close to the Dol-P/UDP-GlcNAc interface on the critical CL3 loop that forms one side of the active site (Figure 4E) and the variant Gly192Ser (Belaya et al., 2012) lies within the highly conserved CL5 loop in the uridyl recognition pocket (Figure 4E) where it would disrupt substrate binding. Two CDG-Ij mutations (Leu168Pro and Tyr170Cys, Iqbal et al., 2013, Wu et al., 2003) are of particular interest as they lie on the ER side of the membrane, at the top of the predicted Dol-P binding site, an extensive, conserved intramembrane groove between TMH4, TMH5, and TMH9, below the EL4 loop (Figure 4F). Both mutations cause a reduction in thermostabilization by Dol-P, confirming their involvement in Dol-P binding. The Met108Leu mutation lies within a hydrophobic cluster in the core of DPAGT1 where a mutation would disrupt the positions of TMH4 and TMH5, which form the back of the Dol-P binding site (Figure 4F). Conversely, many of the mutations that maintain at least 50% of WT activity lie on the protein surface (e.g., Pro30Ser, Ala114Gly, Val117Ile) and involve small sidechain changes (Figure 4C).

Given that DPAGT1-CMS is a recessive disorder, we were surprised to find an increased enzymatic activity with the Val264Gly and Gly160Ser variants. Val264 is mutated to either Gly or Met (Selcen et al., 2014), giving either a 2.5-fold increase or a slight (18%) decrease in catalytic activity. Val264 is located on TMH8 in the core of the protein adjacent to TMH3/4 (Figure 4G). Comparing the WT and Val264Gly structures showed a small (1–1.5 Å) movement at the C-terminal end of TMH4b towards TMH8 (Figure 4G), which would affect both the dimer interface and the exact position of EL4, which forms the top of the Dol-P lipid-binding site. Conversely, the Val264Met variant would be poorly accommodated at this buried site. As the Gly160Ser mutation lies in the disordered EL4 luminal loop (Figure 4F), it is unclear why the activity increases. Since these missense variants cause an unexpected increase in enzyme activity, we explored other causes of pathogenicity. Tissue from muscle biopsies were unavailable, so we used the “exon trap” system to detect abnormal RNA splicing. Both mutations, c.478G>A, p.Gly160Ser, and c.791T>G, p.Val264Gly, gave rise to abnormal RNA splicing of their respective RNA transcripts (Figure S3), thus explaining the pathogenicity of these variants.

### Development of Non-toxic “TUN-X,X” Analogues of Tunicamycin

The potent “off-target” inhibitory effects of tunicamycin on DPAGT1 prevent its use as an antibiotic. We used structural data coupled with genetics to design analogues (**TUN-X,X**) that retained anti-microbial activity yet no longer inhibited DPAGT1. We previously cloned and sequenced the tunicamycin biosynthetic gene cluster (*tun*) from *Streptomyces chartreusis* and expressed it heterologously in *Streptomyces coelicolor* (Wyszynski et al., 2010, Wyszynski et al., 2012). The cluster contains 14 genes, *tunA-N*. In-frame deletion mutations in all of the cloned *tun* genes in *S. coelicolor* (Widdick et al., 2018) revealed new insights into tunicamycin biosynthesis. Interestingly, deletion of *tunI* and *tunJ*, encoding components of an ABC transporter conferring immunity to tunicamycin, could only be achieved with mutations elsewhere in the *tun* gene cluster. Sequencing of one of the Δ*tunI* mutants revealed a G-to-A missense suppressor mutation in *tunC*, resulting in a Gly70Asp substitution in the N-acyltransferase TunC that attaches the lipid of tunicamycin. This presumed loss of function mutation abolished antibacterial activity, consistent with a key role for the lipid chain in the biological activity of tunicamycin.

**Figure 5:**
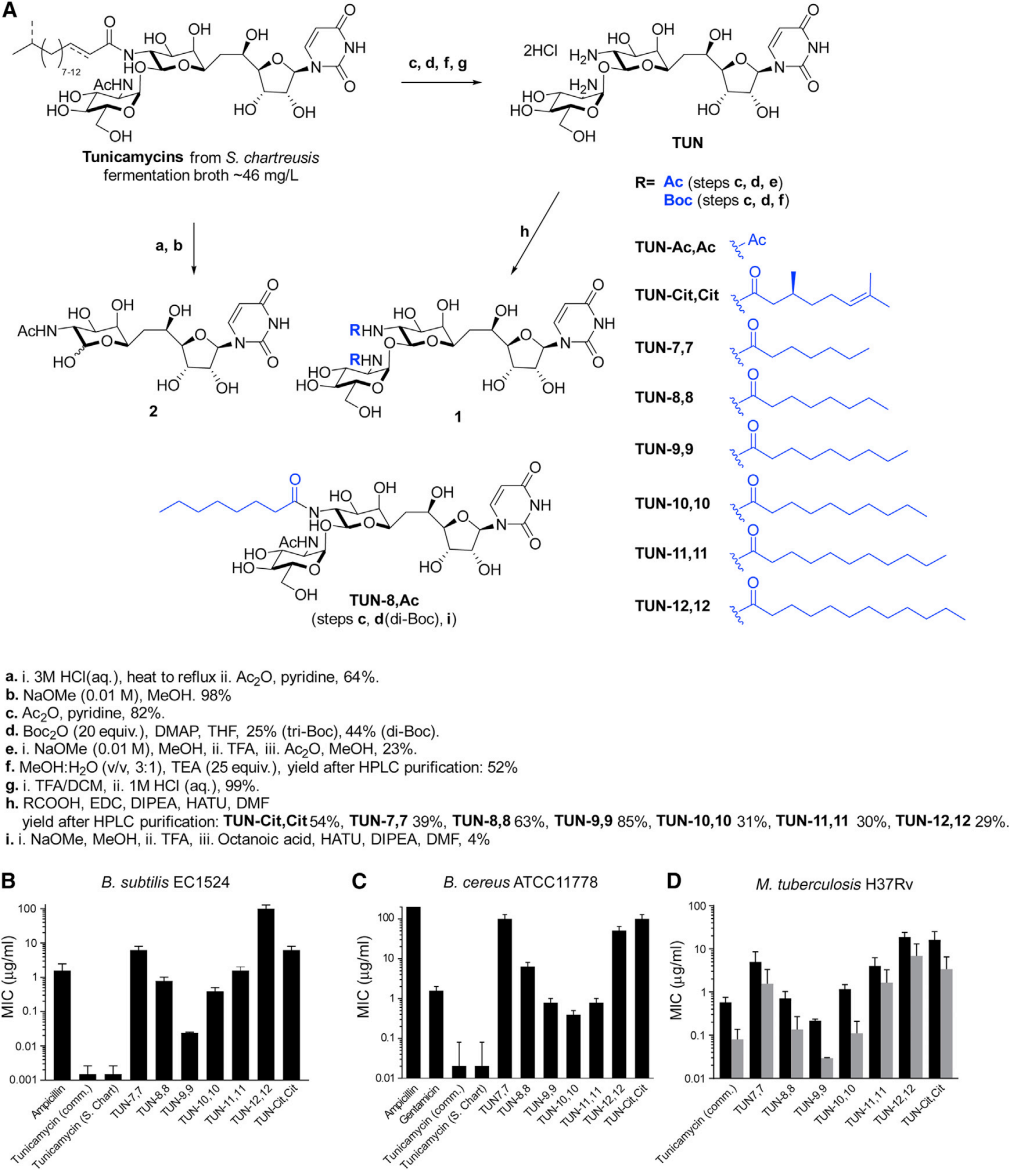
Semi-synthetic Synthesis and Antibacterial Effects of TUN-X,X Analogues (A) Semi-synthetic strategy for TUN mimics. (B–D) MIC obtained from micro-broth dilution antimicrobial susceptibility tests of (B) *B. subtilis* EC1524, (C) *B. cereus* ATCC11778, (D) *Mtb* H37Rv (ATCC27294) cultured in 7H9/ADC/Tw (black), or GAST/Fe (grey) media (n=3). For all panels data presented are means ± SD

We therefore designed a semi-synthetic strategy to access systematically “lipid-altered” variants of tunicamycin based on tunicamine scaffold **TUN** (Figure 5A). Large-scale fermentation of *S. chartreusis* NRRL 3882 (Doroghazi et al., 2011) (Methods S1) allowed access to crude tunicamycin on a multi-gram scale. Degradative conversion (Ito et al., 1979) of tunicamycin gave unfunctionalized core scaffold **TUN**. Critically, since the nucleobase of tunicamycin is hydrolytically sensitive, the creation of mixed Boc-imides at positions 10ʹ and 2ʹʹ allowed mild, selective deamidation on a gram-scale (see Supplemental Information SI 2). Chemoselective carbodiimide- or uronate-mediated acylation allowed direct lipid-tuning in a systematic, divergent manner through modification at 10ʹ-N and/or 2ʹʹ-N, yielding a library of novel analogues, **TUN-X,X** varying in chain length by one carbon, from C7 to C12 (**TUN-7,7** to **TUN-12,12**, Figure 5A) with a typical purity of >99% (see Methods S1) as judged by NMR and/or HPLC.

### TUN Analogues Show Potent Antimicrobial Activity against a Range of Bacteria

**Figure S4:**
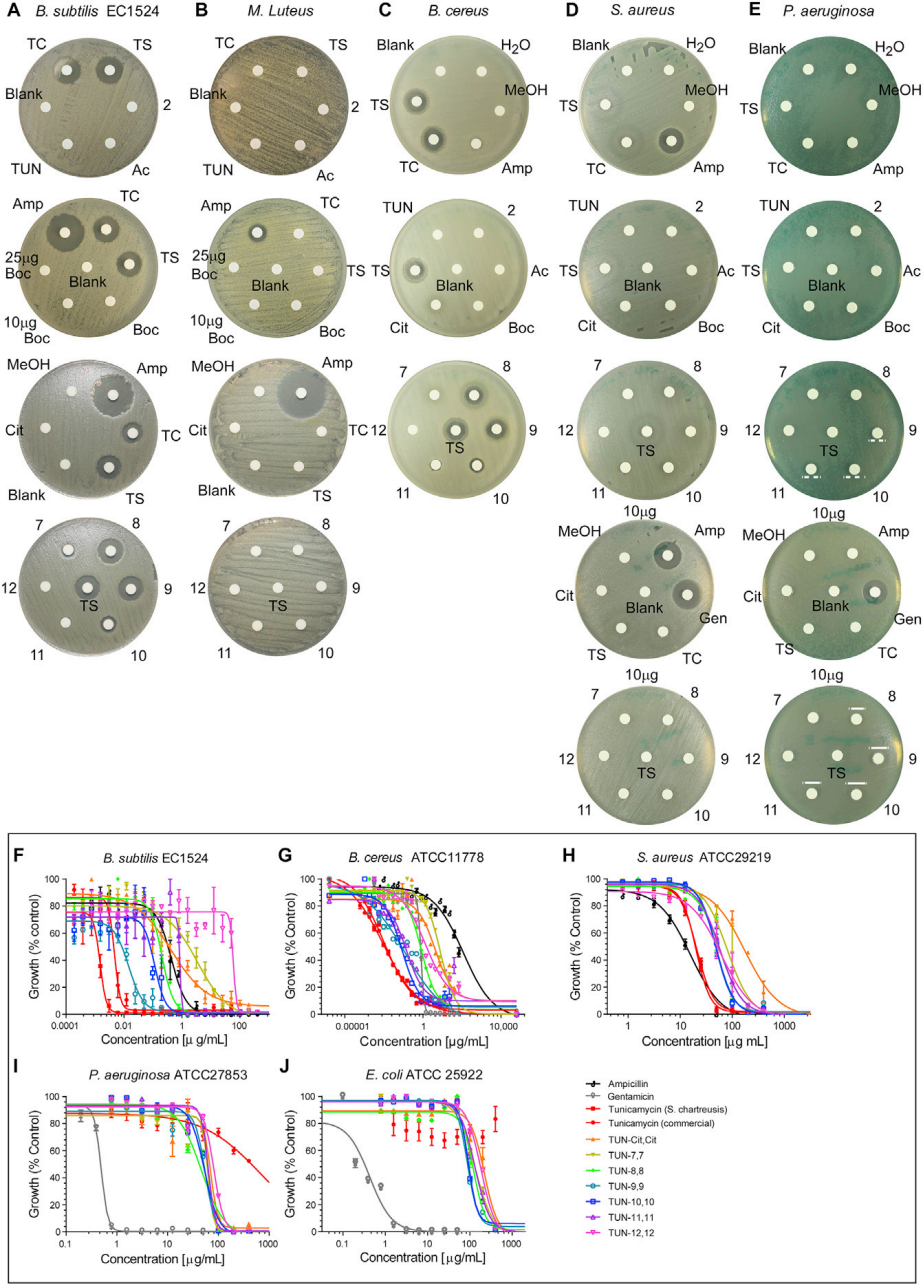
Kirby-Bauer Disc Diffusion Tests and Dose Response Curves Used for MIC, MBC, and IC_50_ Determination for Tunicamycin and the TUN-X,X Analogues against Several Bacterial Strains, Related to Figure 5 (A–E) Kirby-Bauer disc diffusion tests for the TUN analogues against bacterial strains (A) *B. subtilis* EC 1524, (B) *M. luteus*, (C) *B. cereus* ATCC 11778, (D) *S. aureus* ATCC 29219 and (E) *P. aerugino*sa ATCC 27853. Discs impregnated with 5 μg, unless otherwise indicated, of the compound were laid onto plates with lawns of bacteria. The compounds are labelled: TC: commercial tunicamycin, TS: tunicamycin from *S. chartreusis*, 2: (**2**) N-acetyl tunicamine, Ac: **TUN-Ac,Ac**, Boc: **TUN-Boc,Boc**, 7: **TUN-7,7**, 8: **TUN-8,8**, 9: **TUN-9,9**, 10: **TUN-10,10**, 11: **TUN-11,11**, 12: **TUN-12,12**, Cit: **TUN-Cit,Cit**, Amp: ampicillin, Gen: gentamycin. (F–J) Dose response curves for tunicamycin and the analogues with (F) *B. subtilis* EC1524. (G) *B. cereus* ATCC11778. (H) *S. aureus* ATCC29219. (I) *P. aerugino*sa ATCC27853 and (J) *E. coli* ATCC25922. These dose-response curves were generated by Prism 6.0 software by plotting percent growth (normalized OD_600_ values) vs. logarithmic scale of the concentrations. The data shown are mean ± SEM errors of three independent experiments. See Table S3 for the MIC, MBC and IC_50_ values.

We evaluated the analogues (**TUN-7,7, -8,8, -9,9, -10,10, -11,11, -12,12**) for potency against a range of Gram-negative and Gram-positive bacteria that cause infections in hospitals (*Staphylococcus*, *Pseudomonas*, and *Escherichia)*, as well as reference bacteria (*Bacillus*, *Staphylococcus*, and *Micrococcus*) used in previous tunicamycin bioactivity studies (Ward, 1977). Kirby-Bauer disc diffusion susceptibility tests (Figures S4A–S4E), revealed potent activity against *Bacillus subtilis* (EC1524) and opportunistic pathogen *Bacillus cereus* (ATCC 11778). There was a weaker but significant effect on the pathogenic bacteria *Staphylococcus aureus* (ATCC 29219) and *Pseudomonas aeruginosa* (ATCC 27853); the latter is a strain resistant to natural tunicamycin. No activity was seen against *Micrococcus luteus*, a bacterium noted to have some resistance to tunicamycin (Ward, 1977). Consistent with the critical role of the lipid, none of the non-lipidated analogues (e.g., **TUN** or **TUN-Ac,Ac**) or synthetic intermediates showed any activity. Lipid-length (**X** = 7, 8…12) in the **TUN-X,X** analogues systematically modulated activity; the most potent analogues **TUN-8,8** and **TUN-9,9** were those with C8 and C9 chain lengths.

**Figure S5:**
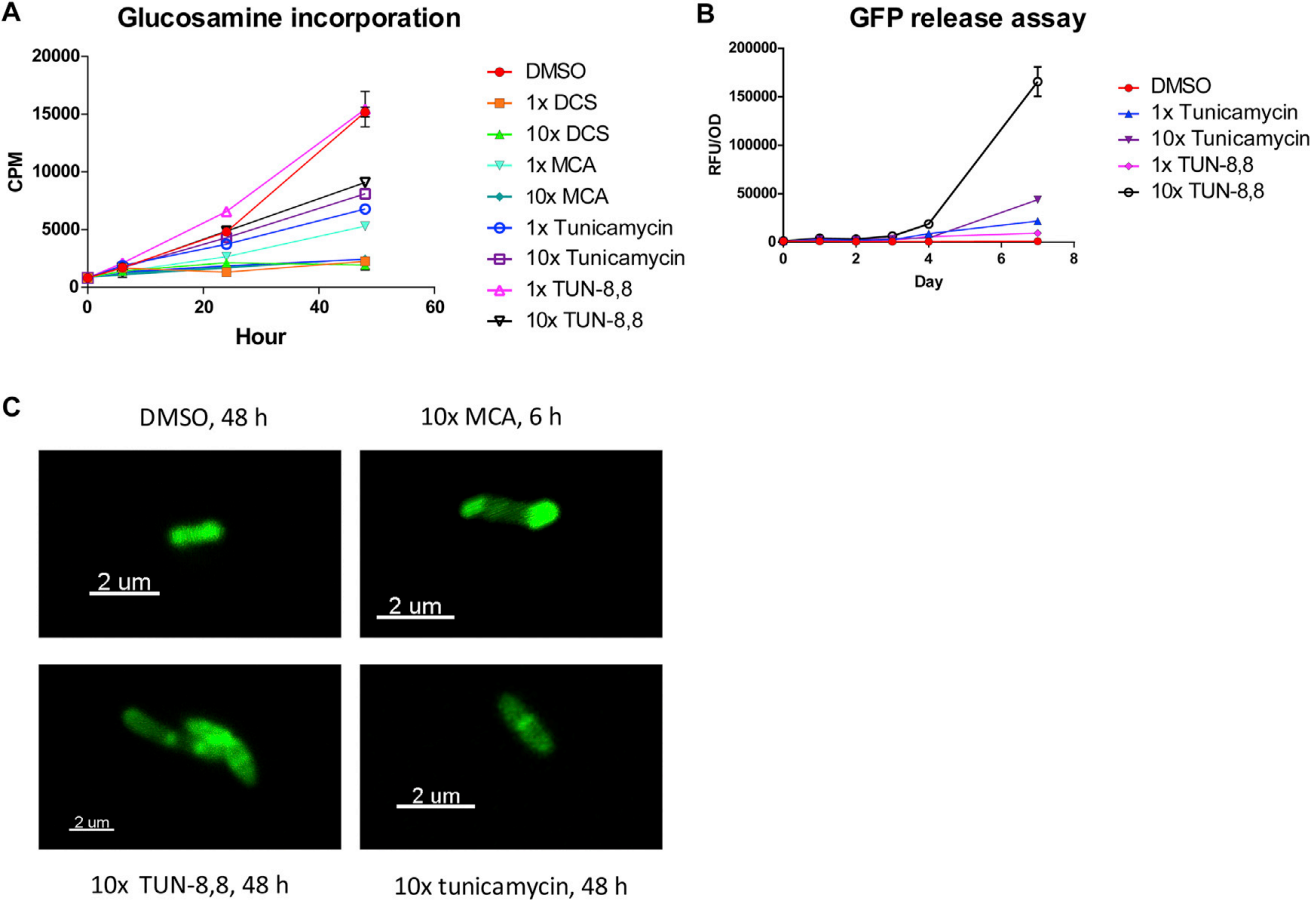
On-Target Effects Demonstrated for the TUN-8,8 Analogue, Related to Figure 5 (A) Glucosamine incorporation within 48 h was reduced with 10x **TUN-8,8**. Positive controls were tunicamycin, meropenem/clavulanate (MCA) and D-cycloserine (DCS). All compounds tested at 1- and 10-fold MIC concentrations (n=2). (B) GFP release assay. *Mtb* expressing GFP was treated with 1- and 10-fold MIC concentrations of **TUN-8,8** or tunicamycin. Lysis was monitored by measurement of fluorescence in cell-free supernatants daily over a week of exposure as a measure of release of cytosolic protein (GFP) (n=2). (C) Confocal microscopy of BODIPY-vancomycin used to track synthesis of PG, showing abnormal PG formation with tunicamycin and **TUN-8,8** treated *Mtb,* both differing from the effects of pentapeptide labelling by the BODIPY-vancomycin caused by meropenem/clavulanate treatment. Data presented in (A) and (B) are means ± SD.

The minimal and half maximal inhibitory concentrations (MIC and IC_50_) and minimal bactericidal concentrations (MBC) were determined by both a micro-broth dilution test and drop plate test, respectively (Figures 5B and 5C, Figures S5F–S5J, Table S3). Only lipidated variants (tunicamycin and **TUN-X,X**) displayed antibacterial activity, with MICs down to 0.02 ± 0.01 μg/ml for **TUN-9,9** against *B. subtilis* and 0.33 ± 0.11 μg/ml against *B. cereus*, with **TUN-10,10**.

TB, through its etiological agent *Mycobacterium tuberculosis* (*Mtb*) is a global concern. Testing of the lipid-altered analogues (**TUN-7,7** to **-12,12**) against pathogenic *Mtb* strain H37Rv revealed striking MIC values (0.03 ± 0.001 μg/ml in minimal growth medium and 0.22 ± 0.02 μg/ml in rich 7H9-based growth medium) for **TUN-9,9**: some 5-fold more potent than even tunicamycin itself (Figure 5D, Table S4).

The on-target effect of tunicamycin analogues was explored by multiple approaches. Firstly, **TUN-8,8**-resistant *Mtb* mutants (Methods S1) carried mutations in Rv0751c (*mmsB*, 3-hydroxyisobutyrate dehydrogenase), an enzyme in fatty acid metabolism, suggesting possible inactivation of **TUN-8,8** by destruction of fatty acid/lipid or an intergenic mutation between Rv2980 and Rv2918c (the central D-alanine-D-alanine ligase *ddlA* involved in peptidoglycan (PG) synthesis), suggesting possible regulation of this key PG biosynthetic enzyme (perhaps via small regulatory noncoding RNA). Secondly, macromolecular incorporation assay using ^14^C-glucosamine as radiolabeled precursor (Figure S5A) confirmed that **TUN-8,8** and tunicamycin have similar effects on PG biosynthesis, suggesting that they act on the same target. Thirdly, extracellular release of green fluorescent protein (GFP) from *Mtb* expressing GFP showed lytic effects of **TUN-8,8**, even outstripping those of tunicamycin (Figure S5B), consistent with on-target PG inhibition activity. Finally, fluorescently-labeled vancomycin (Daniel and Errington, 2003, Tiyanont et al., 2006) revealed similar phenotypes (Figure S5C) following treatment with **TUN-8,8** or tunicamycin with disrupted, non-uniform cell-wall, consistent with targeting of the PG pathway. Together these data suggested that **TUN-8,8** is inhibiting the same target as tunicamycin in *Mtb*.

### Lipid-altered TUN-X,X Analogues are Non-toxic to Eukaryotic Cells

**Figure 6:**
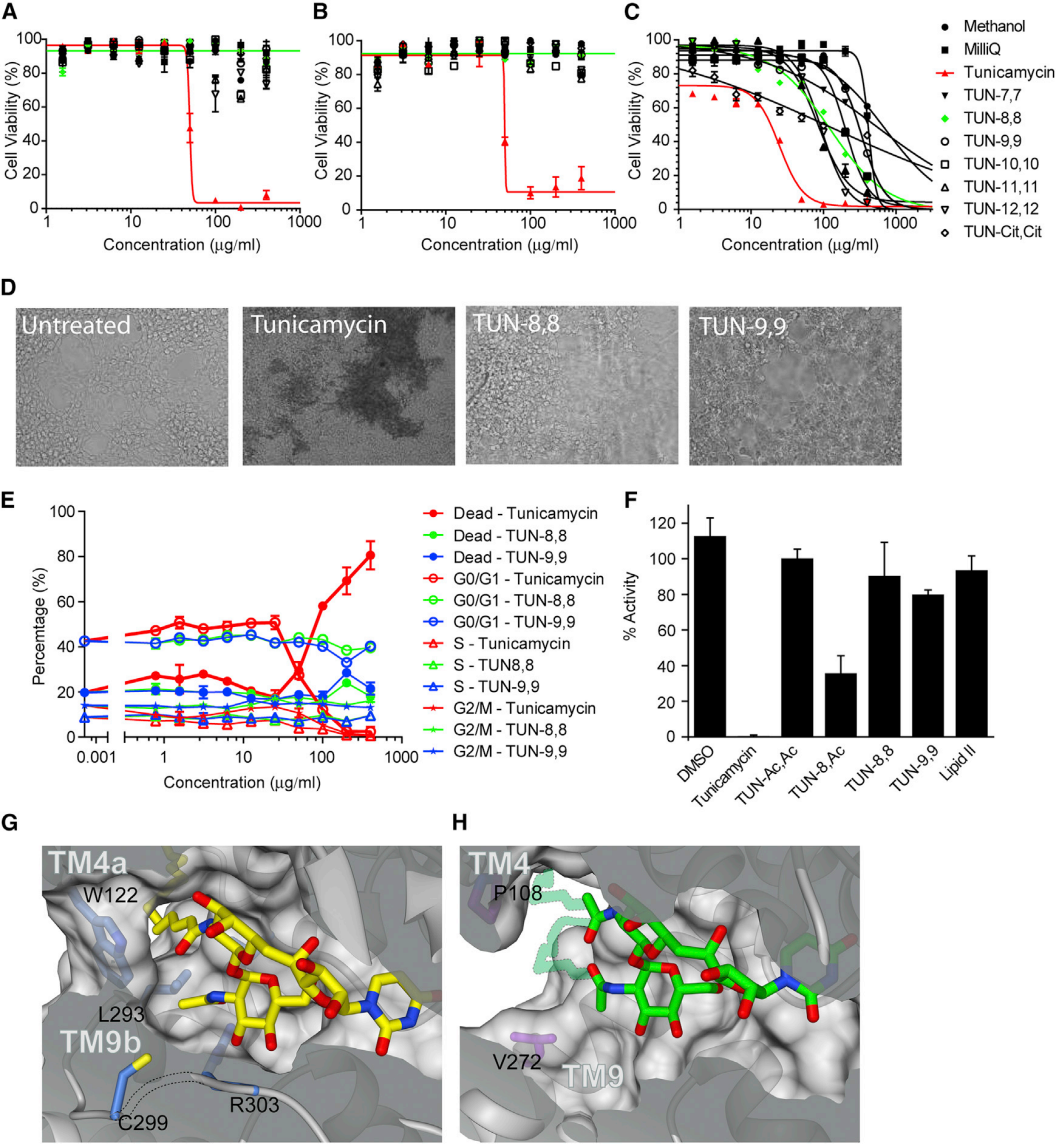
TUN-X,X Analogues are Not Toxic to Cultured Human Cells (A–C) Dose response curves from cell proliferation assays with (A) HEK293, (B) HepG2 and (C) Raji cells with tunicamycin and analogues (n=3 in each case). (D) Effect of 400 μg/ml (saturating) tunicamycin, **TUN-8,8** and **TUN-9,9** on HEK293 cell morphology. (E) Effects of tunicamycin, **TUN-8,8** and **TUN-9,9** on HEK293 cell cycle (n=3). (F) Effects of tunicamycin and **TUN-X,X** analogues on DPAGT1 catalytic activity (n=9). (G) DPAGT1 lipid-binding site, showing the restrictive tunnel with tunicamycin bound. (H) More open MraY lipid-binding site (PDB: 5JNQ), with modelled lipid chains shown with lower contrast. For panels (A–C) and (E), data presented are means ± SEM. For (F), data presented are means ± SD.

**Figure S6:**
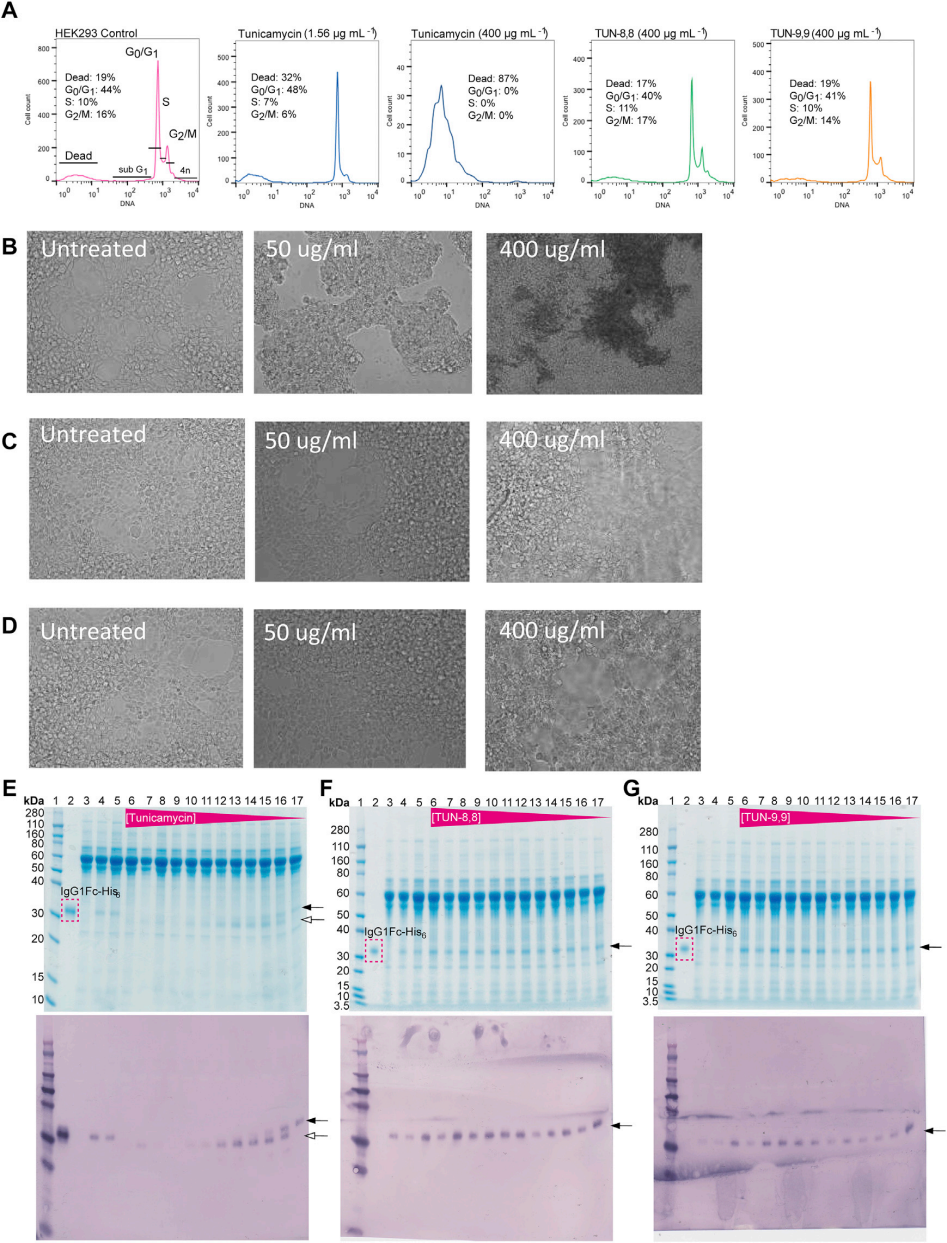
Effect of Tunicamycin and Analogues on HEK293 Cells, Related to Figure 6 (A) Cell cycle analysis at 24 hours. (B–D) Cell morphology with (B) tunicamycin, (C) **TUN-8,8** and (D) **TUN-9,9**. (E–G) Effects of (E) tunicamycin, (F) **TUN-8,8** and (G) **TUN-9,9** on glycosylation of a model protein – IgG1Fc-His^6^. Black arrow indicates glycosylated protein, white arrow indicates unglycosylated protein.

We evaluated the effect of **TUN-7,7** to **-12,12** and corresponding synthetic intermediates on representative human cell lines from liver (HepG2), kidney (HEK293), and blood (Raji) cells where 24 hour incubation with tunicamycin showed both clear cytotoxicity (Figures 6A–6C, Figure S6) and morphological changes (Figure 6D, Figures S6B–S6D) consistent with prior observations (Takatsuki et al., 1972). Cell-cycle analysis (Figures 6E and S6A) suggested cell death coincides with decline in G0/1 phase populations with an LC_50_ ∼100 μg/ml.

Consistent with a mode of action requiring lipidation for toxicity, all non-lipidated variants (**TUN** core and synthetic intermediates) displayed no significant adverse effects; these variants do not act upon *either* bacteria *or* mammalian cells in any potent manner.

In contrast to tunicamycin’s toxicity (LD_50_ = 51.25 ± 31.27, 44.74 ± 4.73, 26.82 ±11.46 μg/ml for HEK293, HepG2 and Raji cells, respectively, Figures 6A–6C and Table S5) the designed **TUN-X,X** variants **TUN-7,7** to **-12,12**, with *altered* lipids, showed mild or negligible toxicity (LD_50_ > 400 μg/ml, saturation) towards mammalian cells. A high level (>75%) of viable cells with no morphological changes were observed after 24 hours (Figure S6A) for HepG2 or HEK293 cells (400 μg/ml, saturation). Moreover, no variation in cell cycle was observed, with healthy G0/1 populations maintained at even highest concentrations (Figure 6E, Figure S6A).

The mechanistic origin of reduced toxicity was tested *in vitro* with purified DPAGT1 enzyme. Whilst native tunicamycin completely inhibited DPAGT1, **TUN-8,8** and **TUN-9,9** had a negligible effect (Figure 6F), consistent observations for glycosylation of model protein (Figures S6E–S6G). Notably, synthetic reinstallation of a *single* C8 lipid into analogue **TUN-8,Ac** restored inhibitory activity towards DPAGT1 (Figure 6F), consistent with our design and the critical role of the second lipid preventing binding to DPAGT1. Given that in patients with CMS, a loss of the activity of one allele is not sufficient to cause disease, it is likely that a reduction in activity by <10%, caused by the **TUN-X,X** analogues, is unlikely to cause significant toxicity during a short-term treatment. Together these results confirmed our hypothesis that systematic “lipid alteration” could create tunicamycin analogues in which mammalian cytotoxicity is separated from antibacterial effects.

#### A Molecular Explanation for Differences in TUN-X,X Analogue Binding to DPAGT1 and MraY

Comparison of the structures of the complexes of tunicamycin with DPAGT1 and MraY (Hakulinen et al., 2017, Yoo et al., 2018; this work) gave an explanation for selectivity of analogues on MraY over DPAGT1. The MraY tunicamycin binding site has a more open, shallow surface than in DPAGT1; in the latter the lipid tail is completely enclosed by Trp122 adjacent to the active site (Figure 6G). The MraY binding site has a disordered loop CL1, a longer TMH9 and a relatively short CL9 region, with only one short α-helix (Figure 1G). In contrast, DPAGT1 has an ordered CL1 which folds over the UDP-binding site. It has a shorter TMH9, followed by a loop and extended strand (residues Gln292 to Arg306) (Figure 1F), which fold over tunicamycin, forming numerous interactions, (e.g., with Arg301, His302, Arg303, Figures 2G and 2H). This extended structure is stabilized by its interactions with the rest of the CL9 domain, a feature found only in eukaryotes.

The N2′′ in GlcNAc is the attachment site of the second lipid chain in **TUN-X,X** analogues—it occupies distinct environments in the two proteins. In DPAGT1 it is enclosed by the loop at the end of TMH9, and by a tight “gating” cluster of side chains from Trp122, Ile186, Leu293, Cys299, and Arg303 (Figure 6G). By contrast, in MraY, there is a 10 Å gap between Pro108 on TMH4a and Val272 on TMH9, providing ample space for more than one lipid chain to be attached to the amines in **TUN-X,X** analogues (Figures 6G and 6H).

### Lipid-altered TUN-X,Xs Show Efficacy against *Mtb* in Mice

**Figure 7:**
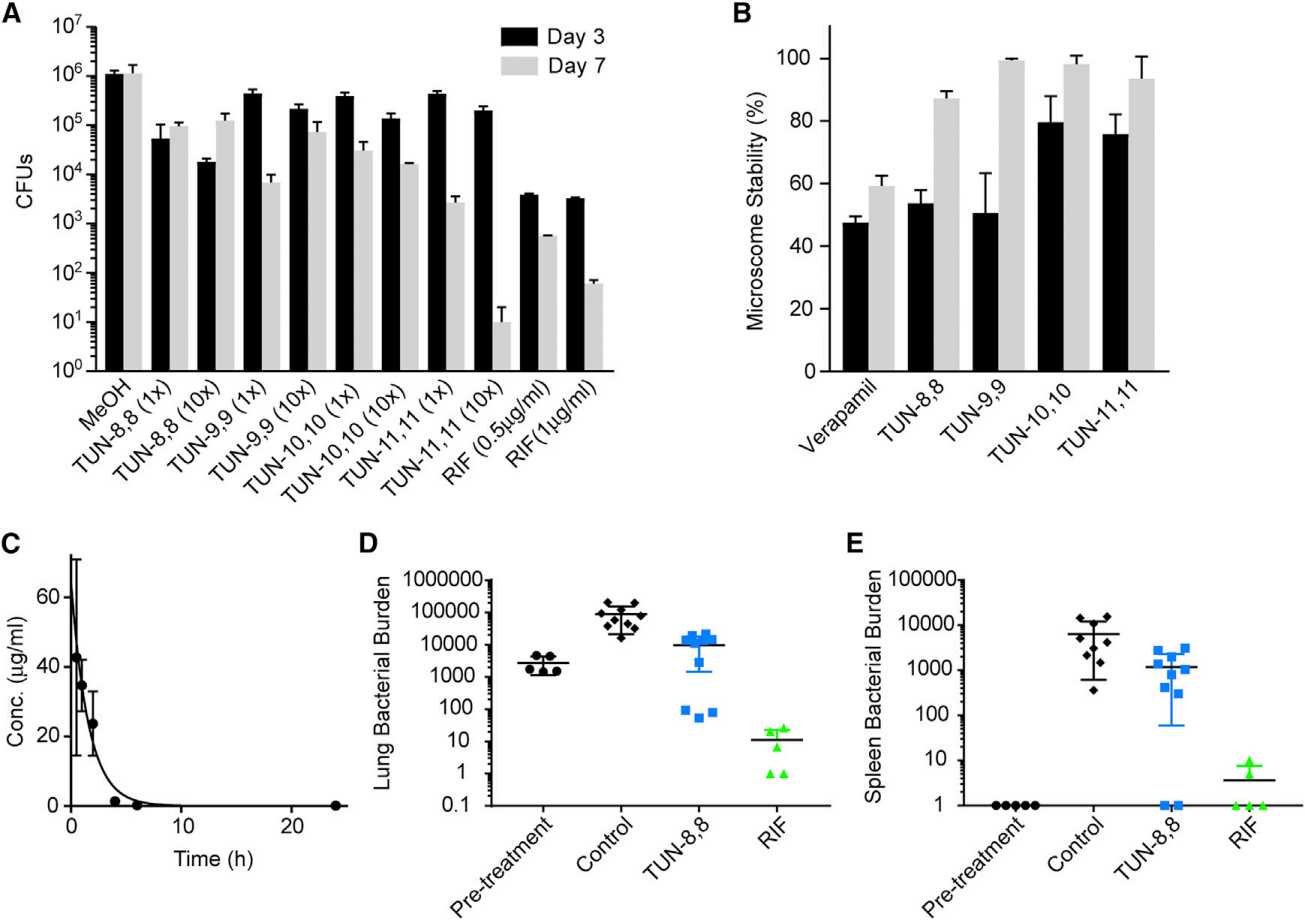
Tunicamycin Analogues Eradicate *Mtb* during Host Pathogenesis (A) Efficacy of tunicamycin analogues in macrophages. Infected J774A.1 macrophages were treated with compounds (1x and 10x MIC) for 3 or 7 days after which bacterial burdens were counted (n=3). (B) Microsomal stability of the tunicamycin analogues in human (black) and mice (grey) liver microsomes assessed over a period of 30 mins (n=3). (C) Mouse blood serum **TUN-8,8** concentrations after a single intra-peritoneal injection of 30 mg/kg (n=5). (D) Efficacy of **TUN-8,8** in reducing lung bacterial burdens in *Mtb*-infected mice after 2 weeks of treatment. (E) Efficacy of **TUN-8,8** in reducing spleen bacterial burdens in *Mtb*-infected mice following 2 weeks of treatment. Unpaired *t* test for the **TUN-8,8** and vehicle control group p value of 0.0017 in lungs and 0.01 in spleens. For all panels (A–C), data presented are means ± SD. For panels (D) and (E), the individual median calculated for each individual mouse organ with standard error of the mean calculated for each group is shown.

The enhanced therapeutic index of the **TUN-X,X** analogues in culture (Table S6) suggested strong potential in treating mammals. Toxicity and efficacy were probed in infection models of *Mtb* both *in cellulo* and *in vivo*. First, as a stringent *in cellulo* test of the ability to treat infection, **TUN-8,8**, **TUN-9,9**, **TUN-10,10,** and **TUN-11,11** were used to treat *Mtb*-infected macrophages (Figure 7A), which showed that these analogues reduced intracellular bacterial burdens by 1- and 2-logs at 1 × and 10 × MIC, respectively.

Second, microsomal (human and mouse) stability (Figure 7B) suggested good metabolic survival of **TUN-8,8** to -**11,11**, which was confirmed by *in vivo* pharmacokinetics in mice (Figure 7C). This revealed good bioavailability of **TUN-8,8** following intraperitoneal (*ip)* delivery with acceptable blood plasma exposures. In human plasma protein binding assays, 98.2% was bound. Next, tolerance testing of **TUN-8,8** in uninfected mice (n=5) over 10 days at daily doses of 30 mg.kg^-1^ (*ip*) showed no signs of toxicity, in striking contrast to tunicamycin. Finally, antitubercular activity was demonstrated in *Mtb*-infected mice. Treatment of *Mtb*-infected mice (n=10) over two weeks (10 mg.kg^-1^, *ip*) revealed an almost 10- and 5-fold reduction in bacterial burdens in lungs (Figure 7D) and spleens, respectively, (Figure 7E) compared to mice receiving vehicle control. Notably, despite the tolerability at 30 mg.kg^-1^ up to 10 days in uninfected animals, clinical signs of toxicity in infected mice precluded any longer-term testing beyond 2 weeks. The origins of this toxicity in diseased animals are unclear but suggest that further optimization of **TUN-X,X** analogues and/or their formulation, may be critical to disease-weakened animals. **TUN-X,X** analogues are therefore unoptimized but promising, proof-of-principle, leads rather than, as yet, optimized antibiotic drugs.

## Discussion

Structures of DPAGT1 allowed us to explain the mechanism of this key enzyme in protein N-glycosylation. We showed that missense variants in DPAGT1 associated with CMS and CDG-Ij alter DPAGT1 function via diverse mechanisms. For many cases of milder CMS disease, severely reduced activity from one allele is combined with an allele with partial activity. In two cases, Val264Gly and Gly160Ser, it appeared that errors in splicing reduced the levels of correct mRNA were partially compensated by 2-fold increases in enzymatic activity. In CDG-Ij, either one allele produces protein with 20% activity or, alternatively, two alleles producing 5%–10% activity, leading to much greater disease severity. In all cases some active protein is present, with a threshold of symptoms and increasing disease severity between no disease at 50% activity and severe disease with 5%–10% of activity. It is significant that DPAGT1 activity can be increased by point mutations at single sites, suggesting it may be possible to increase enzymatic activity and/or modulate stability with small molecules, e.g., pharmacological chaperones (Convertino et al., 2016, Sánchez-Fernández et al., 2016).

DPAGT1 is an “off-target” for the natural bactericidal tunicamycin. Comparison of the human PNPT DPAGT1 and bacterial PNPT MraY structures revealed a gating loop (residues Cys299-Arg303) in DPAGT1 next to where the N-2′′ atom of tunicamycin binds, that is absent in the more open structure of MraY. This difference allowed design of analogues **TUN-X,X** with two lipid chains targeted to bind MraY, but not DPAGT1. This circumvented the toxicity problem normally observed with tunicamycin. Additive modes of action against other carbohydrate-processing enzymes, such as *Mtb* WecA or TagO/TarO (Ishizaki et al., 2013); (Santa Maria et al., 2014), may also be important for the effects of the analogues.

*Mtb* is responsible for ∼1.3 million deaths per annum with increasing spread of drug resistant strains requiring new strategies (Young et al., 2008, Zumla et al., 2013). We have shown that the **TUN-X,X** lipid analogues have much lower toxicity than tunicamycin itself, are effective in killing *Mtb in vitro, in cellulo*, and *in vivo*. The analogues did show some toxicity in mice after more than 2 weeks in diseased (but not healthy) animals, yet they are still much less toxic than tunicamycin. While these lead versions of the **TUN-X,X** lipid analogues are not as effective as the frontline drugs rifampicin and isoniazid in macrophages and in mice *in vivo*, details of the effects in mice are often not recapitulated in humans. In addition, we do not yet have data on intracellular uptake in animals, which may be affecting the outcome. MICs show these compounds are excellent leads for the design of novel antibiotics with a new mechanism of action. **TUN-X,X** lipid analogues are effective antibacterials, with limited toxicity in human cells and in mice (at least with short term dosing), and these and other analogues (Price et al., 2017b) (Price et al., 2017a) suggest a novel approach to development of antibiotics against Gram-positive bacteria.

## STAR★Methods

### Key Resources Table

**Table:** 

REAGENT or RESOURCE	SOURCE	IDENTIFIER
**Bacterial and Virus Strains**		

MAX Efficiency DH10Bac™	Thermo Fisher	Cat# 10361012
Biological Samples		
Mouse liver microsomes	Sigma	Cat# M9441
Human liver microsomes	Sigma	Cat# M 0317

**Chemicals, Peptides, and Recombinant Proteins**		

Tunicamycin	Sigma-Aldrich	Cat# T7765; CAS: 11089-65-9
uridine diphosphate n-acetyl [1-14C] D-glucosamine	American Radiolabeled Chemicals	Cat# ARC 0151
Rifampicin	Sigma-Aldrich	Cat# R3501
Uridine 5′-diphospho-N-acetylglucosamine sodium salt	Sigma-Aldrich	Cat# U4375
C95-Dolichyl-MPDA	Larodan	Cat# 67-1095
*H. sapiens* DPAGT1 (1-408, N-terminal 6 x HIS tag, TEV cleavage site)	This work	N/A
*H. sapiens* DPAGT1 (1-408, Val264Gly, N-terminal 6 x HIS tag, TEV cleavage site)	This work	N/A

**Critical Commercial Assays**		

MemGold2 HT-96 screen	Molecular Dimensions	Cat# MD1-64

**Deposited Data**		

*H. sapiens* DPAGT1 (WT)	This work	PDB: 6FM9
*H. sapiens* DPAGT1 (Val264Gly)	This work	PDB: 5LEV
*H. sapiens* DPAGT1 (Val264Gly) + UDP-GlcNAc + Mg^2+^	This work	PDB: 6FWZ
*H. sapiens* DPAGT1 (Val264Gly) + Tunicamycin	This work	PDB: 5O5E

**Experimental Models: Cell Lines**		

*Spodoptera frugiperda* (Sf9) insect cells	Thermo Fisher	Cat# 11496015; RRID:CVCL_0549
HepG2	ATCC	Cat# ATCC HB-8065
HEK293	ATCC	Cat# ATCC CRL-1573; RRID:CVCL_0045
Raji	ATCC	Cat# ATCC CCL-86; RRID:CVCL_0511
J774A.1	ATCC	Cat# ATCC TIB-67; RRID:CVCL_0358

**Experimental Models: Organisms/Strains**		

*Streptomyces chartreusis*	DSMZ	Cat# 41447
*Bacillus subtilis EC1524*	John Innes Centre	N/A
*Micrococcus luteus*	John Innes Centre	N/A
*Bacillus cereus*	DSMZ	Cat# 345
*Escherichia coli*	Thermo Fisher	Cat# ATCC 25922
*Staphylococcus aureus*	Thermo Fisher	Cat# ATCC 29219
*Pseudomonas aeruginosa*	Thermo Fisher	Cat# ATCC 27853
*Mycobacterium tuberculosis* H37Rv	ATCC	Cat# ATCC27294
C57Bl/6 mice	Taconic	B6

**Oligonucleotides**		

Oligonucleotides	See table S8	B6

**Recombinant DNA**		

pFB-LIC-Bse	This paper	N/A
DPAGT1 gene	Source BioScience LifeSciences	IMAGE:2821845
pMSP12::GFP	Addgene	Cat# 30167

**Software and Algorithms**		

Prism 7	GraphPad	www.graphpad.com
X-ray Detector Software (XDS)	(Kabsch, 2010)	http://xds.mpimf-heidelberg.mpg.de/
CCP4 Suite	(Winn et al., 2011)	http://www.ccp4.ac.uk/
PHYRE2 webserver	(Kelley et al., 2015)	http://www.sbg.bio.ic.ac.uk/phyre2
MR_ROSETTA (phenix.mr_rosetta)	(Terwilliger et al., 2012)	https://www.phenix-online.org/documentation/reference/mr_rosetta.html
PHENIX	(Adams et al., 2010)	https://www.phenix-online.org/
BUSTER (v.2.10.2 & v2.10.3)	(Bricogne et al., 2017)	https://www.globalphasing.com/buster/
COOT	(Emsley et al., 2010)	https://www2.mrc-lmb.cam.ac.uk/personal/pemsley/coot/
PyMOL	Schrödinger, LLC	https://www.pymol.org/
MOLPROBITY	(Chen et al., 2010)	http://molprobity.biochem.duke.edu/
STARANISO	Global Phasing Ltd	http://staraniso.globalphasing.org
PDB2PQR	(Dolinsky et al., 2004)	http://nbcr-222.ucsd.edu/pdb2pqr_2.0.0/
UCSF Chimera	(Pettersen et al., 2004)	https://www.cgl.ucsf.edu/chimera/
Mass spectrometry software Qual Browser	Thermo Fisher	Xcalibur 2.2

### Contact for Reagent and Resource Sharing

Further information and requests for resources and reagents should be directed to and will be fulfilled by the Lead Contact (Liz Carpenter, liz.carpenter@sgc.ox.ac.uk).

### Experimental Model and Subject Details

#### Bacteria

The strain and source of all the bacteria used in this study are detailed in the key resources table.

#### Sf9 cell culture

Sf9 cells were cultured in Sf 900 II SFM medium in a 27 °C incubator, rotating at 100 rpm. Sf9 cells were derived from the epithelium of a pupal female *Spodoptera frugiperda*.

#### HepG2 and HEK293 cell culture

HepG2 and HEK293 cells were cultured in DMEM medium supplemented with 10% heat inactivated fetal bovine serum (FBS, v/v). The cultures were maintained in a humidified incubator at 37 °C in 5% CO_2_/95% air. FBS was reduced to 2% for the cell proliferation assay. HEK293 cells were female, and HepG2 cells were male.

#### Raji cell culture

Raji cells were cultured in RPMI-1640 medium supplemented with 10% heat inactivated fetal bovine serum (FBS, v/v). The cultures were maintained in a humidified incubator at 37 °C in 5% CO_2_/95% air. FBS was reduced to 2% for the cell proliferation assay. The Raji cells used were male.

#### J774A.1 cell culture

J774A.1 cells are female mouse monocyte/macrophage cell type grown in DMEM GlutaMAX (Gibco) supplemented with 10% (v/v) heat inactivated fetal bovine serum, 20 mM HEPES and 0.5 mM sodium pyruvate. Cells were maintained in 75 cm^2^ flasks and sub-cultured (1:5) by scraping. The cultures were maintained in a humidified incubator at 37 °C in 5% CO_2_/95% air.

#### Mice

Mouse studies were carried out in accordance with the Guide for the Care and Use of Laboratory Animals of the National Institutes of Health under Animal study protocol numbers LCID 4E. The NIAID Animal Care and Use Committee (NIAID ACUC), a federally mandated committee, approved and oversaw all animal studies done under LCID4E and ensured that all work complied with the U.S. Government Principles for the Utilization and Care of Vertebrate Animals, the Public Health Service (PHS) Policy on Humane Care and Use of Laboratory Animals, the Animal Welfare Act, and all applicable Animal Welfare Regulations. For all studies, murine-pathogen-free 8-week old naïve wild-type female mice were used. Mice were housed in a biosafety level 3 vivarium with 4-5 animals per cage. Animals were not subjected to water or food restrictions and monitored twice daily by veterinary staff for welfare and health with distressed animals as evidenced by signs of lethargy, weight loss or pain, euthanized according the ASP guidelines.

### Method Details

#### Cloning and expression

The WT DPAGT1 cDNA sequence was cloned into the pFB-LIC-Bse expression vector (available from the SGC) with an N-terminal purification tag with a tobacco etch virus (TEV) protease cleavage site, and a 6x His purification sequence. Baculoviruses were produced by transformation of DH10Bac cells. *Spodoptera frugiperda* (Sf9) insect cells in Sf-900 II SFM medium (Thermo Fisher) were infected with recombinant baculovirus and incubated for 65 h at 27 °C in shaker flasks.

#### Site Directed Mutagenesis

DPAGT1 point mutations were generated using three different methods. The CMS causing mutations were created using the QuickChange site-directed mutagenesis kit (Agilent) according to manufacturer’s instructions; the CDG-Ij causing mutations were generated using the two-step overlap extension PCR method, and the mechanistic mutations were created using the Megaprimer method of site-directed mutagenesis. In the first step of the two-step overlap extension PCR method, the 5’ and 3’ portions of DPAGT1 were amplified in separate reactions using LIC-adapted external primers with internal mutagenic primers. In the second step, the first round products were combined with the LIC-adapted primers to amplify the complete DPAGT1 gene, including the desired mutations. The Megaprimer method uses a single oligonucleotide primer containing each desired mutation was synthesized (Eurofins Genomics). A touchdown polymerase chain reaction, using the mutagenic internal primer and either a 5’ or 3’ external primer, was used to generate a “megaprimer”: a truncated gene fragment that contains the mutation. A second PCR reaction, using the megaprimer and the opposite external primer, was performed to generate the full-length mutated DPAGT1. The PCR products generated from the 3 different methods were then cloned into the pFB-LIC-Bse expression vector via ligase-independent cloning. A full list of the oligonucleotide primers and vectors used in this study can be viewed in the Table S8.

#### Purification of DPAGT1 protein for structural and functional studies

Cell pellets from 1 litre of insect cell culture were resuspended in 40ml in lysis buffer (50 mM HEPES, pH 7.5, 5 mM MgCl_2_, 200 mM NaCl, 5 mM imidazole, 2 mM TCEP (added fresh), 5% glycerol, Roche protease inhibitors (1 tablet per 40ml buffer, added on day of use) in warm water, mixing constantly to keep the sample cold. Cells were lysed by two passes through an EmulsiFlex-C3 homogenizer (Aventin). Protein was extracted from cell membranes by incubation of the crude cell lysate with 1% (w/v) OGNG and 0.1% (w/v) CHS for 1 h at 4°C on a rotator. Cell debris and unlysed cells were removed by centrifugation at 35,000 g for 45 mins. Immobilized metal affinity chromatography was then used to purify the detergent-solubilized His-tagged protein by batch binding to Co^2+^ charged TALON resin (Clontech) at 4 °C for 1 h. The resin was then washed with wash buffer (WB: 50 mM HEPES (pH 7.5), 5mM MgCl_2_, 10 mM imidazole (pH 8.0), 200 mM NaCl, 2 mM TCEP (added fresh), 5% Glycerol, 0.18% OGNG, 0.01 8% CHS, 0.0036% cardiolipin) and the protein was eluted with WB supplemented with 250 mM imidazole (pH 8.0). The eluted protein was desalted using PD-10 columns (GE Healthcare) pre-equilibrated with gel filtration buffer (GFB: 20 mM HEPES (pH 7.5), 5 mM MgCl2, 200 mM NaCl, 2 mM TCEP, 0.12% OGNG, 0.012% CHS, 0.0024% cardiolipin). Desalted protein was subsequently treated with 10:1 TEV protease (w:w, protein:enzyme) overnight at 4 °C. The TEV protease treated protein was separated from the 6-His-tagged enzymes and uncleaved DPAGT1 by incubation for 1 h with Talon resin (prepared as described above) at 4 °C for 1h. The resin was collected in a column, the flowthrough collected and the protein sample was centrifuged at 21,500 rpm in a Beckman TA25.5 rotor for 10 min at 4 °C. The supernatant was then concentrated to 0.5 ml using a 30 kDa cutoff PES concentrator (Corning), with mixing every 5 mins during concentration. The concentrated protein was then centrifugated at 20,000 g for 10 min, then further purified by size exclusion chromatography (SEC) on a Sepharose S200 column (GE Healthcare) in GFB. The peak fractions were pooled and concentrated using a Sartorius 2ml PES 50 kDa concentrator (pre-equilibrated with GFB without detergent), at 3220 g. The protein was centrifuged at 20,000g for 15 mins, then flash frozen in liquid nitrogen. The final concentration was 20-30 mg/ml. Denaturing LC-MS was performed on purified protein obtained from each purification as described below. A mass of 46177 Da was obtained for the WT protein, which matches the DPAGT1 monomer (with an additional N-terminal Serine residue from the TEV cleavage site). Similarly the Val264Gly mutated protein gave a mass of 46135 Da. Each mutated gene was sequenced and the purified protein was subjected to denaturing, intact mass spectrometry. In each case the predicted sequence and mass was observed for the mutated gene and protein. In all cases the monomer mass was observed, and there was no evidence for covalent, disulphide-linked dimers. When DPAGT1 was purified in the absence of reducing agents, the same monomer mass was observed. In no case did we observe a peak with the mass of a covalent, disulphide-bonded dimer.

#### Denaturing LC-MS

Intact mass of detergent solubilized DPAGT1 was determined as follows. Samples were diluted to a final concentration of 8-20μg/ml in 30% methanol, 0.1% formic acid. Fifty μl was injected on to a 1290 UPLC coupled to a 6530 QTOF mass spectrometer fitted with a ZORBAX StableBond 300 C3, 2.1 x 150mm, 5 μm HPLC column (Agilent Technologies, Santa Clara, USA). Solvent A was 0.1% formic acid in HPLC grade water; solvent B was 0.1% formic acid in LC-MS grade methanol (Fisher Scientific, Loughborough, UK). Initial conditions were 30% B at 0.5 ml/min. After 1 min a methanol gradient was applied from 30% to 95% over 7 min. Elution was then isocratic at 95% B for 2 min, followed by a further 2 min equilibration at 30% B. Complete separation was achieved between detergent (elution 6.2-8.0 min) and protein (elution 8.2-9.5 min). The mass spectrometer was operated in positive ion, 2 GHz detector mode. Source parameters were drying gas 350 °C, flow 12 l/min, nebulizer 60 psi, capillary 4000 V. Fragmentor was 250 V, collision energy 0 V and data acquired from 100-3200 m/z. Data analysis was performed using Masshunter Qualitative Analysis B0.7 proprietary software and deconvolution performed using the Maximum Entropy algorithm.

#### Radiolabeled substrate enzyme activity assay

2 μl of 2 μM DPAGT1 WT or mutant proteins in GFB buffer supplemented with 5mM extra MgCl_2_, 1% OGNG/CHS/cardiolipin and dolichyl monophosphate was combined with 2 μl of UDP-N-acetyl [1-14C] D-glucosamine in the same buffer and incubated at 37 °C on a heat block for 21 min. The reaction was terminated by the addition of 6 μl of 100% methanol and immediately transferred onto ice. 1 μl of sample was spotted onto a silica coated TLC plate in triplicate and run with a mobile phase consisting of chloroform, methanol, and water at a 65:25:4 ratio respectively. After the run, the TLC plate was dried thoroughly, wrapped in cling film, incubated with a phosphor imaging substrate for 4 days, then phosphor imaged using a Biorad. The pixel density of the spots corresponding to the hydrophobic product was divided by combined pixel density of the product and the substrate and multiplied by the known concentration of substrate added to ascertain the amount of product formed.

#### Thermostability assays for DPAGT1 WT and mutant proteins

Samples with a volume of 40 μl were prepared containing 0.5 mg/ml protein and 50 μM compound or 5% DMSO in GFB. A glass capillary was dipped into each sample, with the capillary held horizontally to ensure that the capillary was full of the sample. The capillaries were placed on the capillary holder on the Nanotemper Prometheus. Technical triplicates of each sample were prepared, and each experiment was conducted with biological triplicates of each protein. PR.ThermControl software was used to run the experiment and analyse the data. A melting curve from 20 °C – 95 °C at 5 °C/min was performed. The minimum of the first derivative of the 330/350nm ratio was used to determine the Tm_1/2_.

#### Crystallization of apo DPAGT1 protein

Protein was concentrated to ∼20 mg/ml, then diluted to 9–12 mg/ml using GFB without detergent. Initial crystals were grown at 4 °C with WT protein purified with DOPG using sitting drop (150 nl) crystallization set up in 96- well format using a Mosquito crystallization robot (TTP Labtech) with protein:reservoir ratios of 2:1, 1:1 and 1:2. Crystals of DPAGT1 were initially obtained in MemGold2 HT-96 screen (Molecular Dimensions) condition G10. Reproducibility for this condition was poor, and protein purification was further optimized. Adding cardiolipin instead of DOPG to purification buffers as well as using the Val264Gly mutant construct improved crystal reproducibility. A new crystallization condition was obtained at 20 °C which was optimized to 32%—38% (v/v) polyethylene glycol (PEG) 300, 50 mM sodium chloride, 0-5 mM sodium tungstate, 0.1 M bicine, pH 9.0. Seeding was used to further improve crystallization reproducibility.

#### Crystallization of the Val264Gly mutant DPAGT1 with UDP-GlcNAc and Tunicamycin

To obtain a co-structure of DPAGT1 with UDP-GlcNAc, Val264Gly DPAGT1 was incubated with 10 mM UDP-GlcNAc for 1 hour at 4 °C before setting up crystallization with seeds at 20 °C in reservoir solution containing 32.5%–38% (v/v) PEG 300, 50 mM NaCl, 0.1 M bicine, pH 9.0. To obtain a co-structure of DPAGT1 with tunicamycin, Val264Gly DPAGT1 harvested after SEC was supplemented with 0.1 mM tunicamycin and incubated for 1 hour at 4 °C before concentrating. The concentrated protein was crystallized at 4 °C in sitting drop crystallization trials with reservoir solution containing 36% (v/v) PEG 200, 50 mM sodium chloride, 0.1 M bicine, pH 8.5.

#### Data collection and structure determination for the DPAGT1 complexes

All data were collected at Diamond Light Source (beamlines I24, I04-1 and I04) to resolutions between 3.2-3.6 Å based on CC_1/2_=0.5 criteria. Data were processed, reduced and scaled using XDS (Kabsch, 2010) and AIMLESS (Evans, 2006) (Table S2). All crystals belong to space group *P*6_5_22 and contain a single DPAGT1 monomer in the asymmetric unit with 70% solvent. Initial phase estimates were obtained using molecular replacement (MR). Briefly, an initial search model was built automatically using the PHYRE2 web server (Kelley et al., 2015) based on the coordinates of the bacterial homolog MraY (19% sequence identity; PDB: 4J72). However, this simple homology model failed to produce any meaningful MR solutions when used in isolation in PHASER (McCoy et al., 2007). The PHYRE model was then subjected to model pre-refinement using the procedures implemented in MR-ROSETTA in PHENIX (Terwilliger et al., 2012) and the resultant five best-scoring output models were trimmed at their termini and in the TMH9/TMH10 cytoplasmic loop region and superposed for use as an ensemble search model in PHASER. A marginal but consistent solution was obtained that exhibited sensible crystal packing in space group *P*6_5_22 but both the initial maps and model refinement were inconclusive. The model positioned using PHASER was converted to a poly-alanine trace and recycled into MR-ROSETTA, using model_already_placed=True option. The resultant MR-ROSETTA output model had an R/Rfree of 42/46 and the electron density maps showed new features not present in the input coordinates that indicated that the structure had been successfully phased.

Using the MR-ROSETTA solution as a starting point, the remaining regions of the DPAGT1 structure could be built manually using COOT (Emsley et al., 2010) using the WT 3.6 Å native data. However, the novel 52 amino acid cytoplasmic insertion domain between TMH9 and TMH10 was poorly ordered and proved difficult to trace. This region was primarily traced using the electron density maps for the UDP-GlcNAc complex as substrate binding results in partial stabilization of the TMH9/10 insertion domain. All electron density maps were sharpened in COOT to aid model building using a *B*-factor of -100 Å^2^. Sequence assignment was aided by using both mercury labelling of cysteines (Figure S2A) and the sulphur anomalous signal from a dataset collected from UDP-GlcNAc complexes crystals at a wavelength of 1.7 Å (Figure S2B). Anomalous difference maps, combined with anomalous substructure completion using PHASER-EP, clearly revealed the location of 18 of the expected 22 sulphur positions and helped to confirm the sequence register (Figure S2B). Additional experimental phasing information was provided by a Pr^3+^ derivative. The resultant model for the entire chain was then refined against both the unbound Val264Gly (3.2 Å), Val264Gly UDP-GlcNAc (3.1 Å), Val264Gly tunicamycin (3.4 Å) as well as the WT unbound (3.6 Å) data using BUSTER v2.10.2 / v2.10.3 and REFMAC (UDP-GlcNAc complex – final cycle only). All data were mildly anisotropic but were used in BUSTER without truncation apart from the unbound Val264Gly dataset which was anisotropically truncated with STARANISO using default cutoffs. Reference model restraints improved the refinement behavior for the 3.6 Å unbound WT structure (using the unbound Val264Gly model as the reference model). Ligand restraints were generated using the GRADE webserver (http://grade.globalphasing.org) and a single TLS group encompassing the entire protein chain was used in refinement. The presence of a single magnesium ion in the UDP-GlcNAc complex was verified using the anomalous signal from a dataset collected at 1.82 Å from a crystal grown in the presence of MnCl_2_. A single peak (19σ) was observed in an anomalous difference Fourier map calculated at 4 Å adjacent to UDP-GlcNAc pyrophosphate; the next highest peak corresponded to various sulphur atoms (5σ). A second putative Mn^2+^/Mg^2+^ site was identified between E94 and H270 on the luminal face (peak height 4.3σ). Elongated lipid–like density on the TMH1/6/7/10 face of DPAGT1 was modelled as a dioleoylphosphoglycerol (DOPG) lipid. This lipid feature was present in both the electron density maps of the WT structure (purified with DOPG added) and the various Val264Gly mutant structures (purified with cardiolipin). The presence of DOPG in the purified protein samples used for crystallization was detected by mass spectrometry (Figure S1I). Lipid-like density was also present in the concave putative Dol-P groove adjacent to the EL4 luminal hairpin and has been modelled as unknown lipid/alkyl chains (UNL) in both the UDP-GlcNAc and TUN complexes. An additional persistent feature in all structures was electron density at the mouth of the active site adjacent to Trp122 (TMH4) and Leu293 (TMH9), presumably arising from a co-purified lipid that mimics the Dol-P substrate binding. However, the density was poorly resolved and no attempt was made to interpret this feature in the final models. The commercial preparation of TUN used for co-crystallization in this study is a natural product and contains a range of different aliphatic chain lengths (n=8-11). A chain length of n=9 was chosen for the modelled TUN as this appeared to most consistent with the observed electron density (Figure S2E).

The representative final model comprises the entire polypeptide chain between residues Leu7 and Gln400 apart from the flexible EL2 loop connecting TMH2 and TMH3 and part of the poorly-ordered EL4 luminal hairpin (residue 152-161).

#### Native mass spectrometry

For native mass spectrometry analysis purified DPAGT1 protein was diluted to 10 μM protein concentration using 200 mM ammonium acetate supplemented with 0.16% OGNG solution followed by buffer exchanged into 200 mM ammonium acetate, 0.16% OGNG using a micro biospin column (Micro Bio-Spin 6, Bio-Rad). Native MS experiments were conducted using a Q Exactive instrument (Thermo Fisher, Germany) with modifications for high-mass transmission optimization. Typically, 2 μl of buffer exchanged protein solution was electrosprayed from gold-plated borosilicate capillaries prepared in house. The instrument was operated under following parameters: 1.2 kV capillary voltage, 100V S-lens, 250 °C capillary temperature, 100 cone voltage. The activation voltage in HCD cell was raised from 100-200 V until a nicely resolve charge state pattern was found. Pressure in the HCD cell was raised to 1.2e^-9^ mbar for efficient transmission of protein. The instrument was operated in positive ion mode and was calibrated using caesium iodide solution.

For protein activity detection, 5 μM DPAGT1 protein was incubated separately with 50 μM dolichol-phosphate and UDP-GlcNAc or together in presence of both substrates at 37 °C for 21 min. Native MS buffer was used to spray the protein in presence of substrates on a modified Q Exactive Plus instrument. For detection of phosphate groups the instrument was operated in negative mode and minimal activation conditions were applied. The instrument was calibrated using cesium iodide. The experiments were repeated three times using three different protein preparations.

#### Lipid analysis by tandem mass spectrometry

Lipidomics analysis was performed on DPAGT1 to identify co-purified lipid using typical reversed-phase liquid chromatography coupled to tandem mass spectrometry method after modifications in liquid chromatography gradient. Protein was digested with trypsin (1:50 units) for overnight at 37 °C in a thermomixer (Eppendorf) under continuous shaking. The digest was dried in a SpeedVac until complete dryness and re-dissolved in 68% solution A (ACN:H2O 60:40, 10 mM ammonium formate and 0.1% formic acid) and 32% solution B (IPA:ACN 90:10, 10 mM ammonium formate and 0.1% formic acid). The tryptic digest mixture was loaded onto a pre-equilibrated C18 column (Acclaim PepMap 100, C18, 75 μm × 15 cm; Thermo Scientific) at a flow rate of 300 nl min^-1^. The lipids were separated under following gradient: In 10 min solvent B was ramped from 2% to 65% over 1 min, then 80% over 6 min, before being held at 80% for 10 min, then ramped to 99% over 6 min and held for 7 min. The nano-flow reversed-phase liquid chromatography (Dionex UltiMate 3000 RSLC nano System, Thermo Scientific) was directly coupled to an LTQ-Orbitrap XL hybrid mass spectrometer (Thermo Scientific) via a dynamic nanospray source. Typical MS conditions were spray voltage of 1.6 kV and capillary temperature of 275 °C.

The LTQ-Orbitrap XL was set up in negative ion mode and in data-dependent acquisition mode to perform five MS/MS scans per MS scan. Survey full-scan MS spectra were acquired in the Orbitrap (m/z 350–2,000) with a resolution of 60,000. The chromatogram was manually analysed for presence of different masses followed by lipids identification by manually comparing their experimental and theoretical fragmentation pattern.

#### Exon Trap Analysis

*DPAGT1* exons 2, 3 and 4 and flanking intronic sequences or exons 6, 7 and 8 and flanking sequences were cloned into the pET01 vector (MoBiTec). c.478G>A and c.791T>G were respectively introduced by site-directed mutagenesis using Quikchange kit from Stratagene and confirmed by Sanger sequencing. Control and mutant vector DNA were electroporated into the human rhabdomyosarcoma cell line TE671 using the NEON electroporator (Invitrogen). Total RNA was purified 48 hr after transfection, reverse transcribed into cDNA using Retroscript kit (Ambion). cDNA was amplified using primers specific to the vector exons. The amplicons were run on agarose/TBE gels, visualized under UV/ethidium bromide and then gel purified and sequenced.

#### Mueller-Hinton Agar Plate

Muller-Hinton agar plate was prepared according to CLSI standards. Oxoid Mueller-Hinton Agar was prepared according to the manufacture’s protocol. 25 ml of the warm agar solution was transferred to 90 mm x 16.2 mm plate via sterile pipette. The agar plate was cooled at room temperature for 15 minutes before use or storage at 4 °C up to two weeks.

#### Kirby-Bauer Disc Diffusion Test

Oxoid Blank Disc was impregnated with the desired test substance. A 0.5 McFarland standard inoculum was prepared by adding 3-5 single colonies to 10 mL MH broth in 15 mL-falcon tube and standardized to 0.5-McFarland standard. The inoculum was used within 10 minutes. A sterile cotton swab was dipped in the inoculum, gently pressed against the side of the tube to remove excess liquid, and generously streaked on MH agar plate to fully cover the plate. The impregnated disc was carefully placed on the agar (important: once the disc touches the agar, it should not be moved). The plate was incubated at 35 °C for 20 hrs overnight. A digital calliper was used to measure the zone diameter. The recorded zone diameter is an average of three zone diameters measured of one zone.

#### Micro-dilution culture to determine minimal inhibition and minimal bactericidal concentrations

In a sterile 96-well plate, serial dilutions were made with the test substance to final volume of 50 μl. Inoculum was then prepared in Mueller-Hinton broth to 0.5 McFarland and diluted before adding 50 μl to the well to make ∼1x10^5^ CFU/ml. The culture plate was incubated at 35 °C for 20-24 hrs. A positive growth control and sterility wells were also prepared along with the culture wells. Absorbance at OD_600_ was taken using BMG Labtech SPECTROstar Omega spectrophotometer. MIC is determined by the lowest concentration without growth. IC50 is determined from plotting a dose-response curve, see Data Analysis below. To determine the MBC, using a multi-channel pipette, 1 μl of culture broth was taken from the same 96-well microdilution growth plate and carefully inoculated on surface of MH agar plate. The plate is then incubated for additional 20 hrs. at 35 °C. The MBC value is the lowest concentration without observed growth on the agar.

#### Cell proliferation assay

In sterile 96-well plate, each well was seeded with ∼1x10^5^ cells. The cells are then grown confluent overnight in 100 μl DMEM with 10% FBS. The medium is replenished with DMEM with 2% FBS and added vehicle control or test substance the next day. For test substance in methanol, stock was added to 25 μl of DMEM with 2% FBS or PBS and placed in the laminar flow hood to let the methanol evaporate (about 1-3 hrs). Once the methanol has evaporated, DMEM with 2% FBS was added to final volume of 100 μl of desired test concentration. A blank methanol control was also made to ensure that any cytotoxicity did not result from methanol contamination. Cells are grown for additional 24 hrs. The cell viability was determined by using Promega CellTiter 96^©^ AQ_ueous_ One Solution Cell Proliferation Assay (3-(4,5-dimethylthiazol-2-yl)-5-(3-carboxymethoxyphenyl)-2-(4-sulfophenyl)-2H-tetrazolium, inner salt; MTS) System following the manufacture protocol.

#### 24 Hr Cell Cycle Test

The cell cycle tests were carried out in 96-well tissue culture plates. A T-75 tissue culture flask was first used to culture a stock of HEK293 cells. Once the cells were approximately 90% confluent, the old medium was decanted and the cells were carefully washed with PBS and resuspended in DMEM with 10% FBS. HEK293 cells are seeded into the 96-well plates (1x10^5^ cells/well). Before placing the culture in the incubator (37 °C with 5% CO_2_), the plate was set aside for 10 minutes for the cells to settle to the bottom of the plate. DMEM with 2% FBS containing the test substance is prepared beforehand. Once the cells were confluent the next day or when they were ready to be treated, the test compound in DMEM with 2% FBS was added and the cells were cultured for the required time.

Once the cell culture was ready for harvesting, the cells were then fixed with cold 70% ethanol after two washes with PBS (w/ 0.1% BSA). The cells were fixed overnight at 4 °C.

#### Propidium Iodide (PI)/RNase Staining Solution from NEB was used for DNA staining

For test substances in methanol, the compound in a methanol containing stock solution was added to 50 ul of DMEM or PBS in a round-well plate (8-well plate) and placed in the laminar hood for 1 – 2 hrs to remove the methanol by evaporation and then the required amount of DMEM with 2% FBS was added to give the desired volume.

#### Time-course Cell Cycle Test

The time-course cell cycle test was carried out in 96-well tissue culture plate. Three sets of plates were prepared as described above with samples prepared in triplicate. A 0 hr control cell culture was first collected before setting up the plates for treatment. Each set is used for 24 hr, 48 hr or 72 hr time-point data collection. Due to nature of the experiment, the DMEM used contained 10% Heat-inactivated FBS for the cells to have enough nutrients to the 72 hr cell cycle. Once the cell culture was ready for harvesting, the cells were then fixed with cold 70% ethanol after two washes with PBS (w/v 0.1% BSA). The cells were fixed overnight at 4 °C. Propidium Iodide (PI)/RNase Staining Solution from NEB was used for DNA staining.

#### IgG1Fc N-Glycosylation Assay

The test can be carried out in 6- or 8-well tissue culture plate. The transfected HEK293T/pHLsec:IgG1Fc cell culture harboring pHLsec:IgG1Fc plasmid with a His_6_-Tag IgG1Fc (Transfection protocol below) was grown for 2-3 days (37 °C with 5% CO_2_). Then the growth medium was collected and analyzed by SDS-PAGE and western blot analysis.

#### Transfection Protocol

Transfection reagents and medium were prepared fresh every time. A PEI (Polyethylimine) stock solution was first prepared (25 kDa, linear -100 μg/mL). PEI was added in MilliQ water and HCl (aq) was then added drop-wise with just enough to help solubilize the PEI. The PEI solution was filtered through 0.2 um membrane in laminar sterile hood. PEI solution was set aside. The DNA stock solution was added to serum-free DMEM medium at room temperature to make final DNA-DMEM concentration of 4 μg/mL. PEI solution was then added to DMEM-DNA solution to make a final concentration of PEI of 4.6 μg/mL. The PEI-DNA transfection medium was gently stirred at room temperature for 20 minutes before use. For the transfection step, the DNA-PEI transfection was added first and placed in the incubator (37 °C with 5% CO_2_) for 10 minutes and then DMEM with 2% FBS was added. The DNA-PEI medium and DMEM with 2% FBS medium were added in 1:3 ratio.

#### Statistical Analysis

Data were analysed using Graph Pad PRISM 5.01 software. Dose-response curves were plotted from three independent data sets with SEM error bars.

#### Minimum inhibitory concentrations testing against *Mtb*

*Mtb* H37Rv ATCC27294 was grown to OD_650nm_ of 0.2 in either Glycerol-Alanine salts medium (GAST/Fe) or 7H9/ADC/Tw. GAST/Fe consisted of per liter: 0.3 g Bacto Casitone (Difco), 4.0 g dibasic potassium phosphate, 2.0 g citric acid, 1.0 g L-alanine, 1.2 g magnesium chloride hexahydrate, 0.6 g potassium sulfate, 0.05 g ferric ammonium citrate, 2.0 g ammonium chloride, 1.80 ml of 10N NaOH, and 10.0 ml of glycerol, 0.05% Tween 80 with pH adjusted to 6.6 before sterile filtration. 7H9/ADC/Tw consisted of Middlebrook 7H9 broth base (Becton Dickinson) supplemented with 0.2% glycerol/0.5% BSA fraction V/ 0.2% glucose/ 0.08% NaCl/ 0.05% Tween 80. Cells were diluted 1000-fold in this medium and an equal volume (50 μl/well) added to 96-well U-bottom plates (Nunclon, Thermo Scientific) containing compound diluted in the respective growth medium (50 μL/well). Dilutions ranged from 0.024 - 50 μg/mL, performed in technical duplicates over two biological repeats. The positive control was isoniazid (tested from 0.024 - 50 μM) and negative control was the solvent. Isoniazid MIC was 0.2 ± 0.1 μM. MIC was determined over 2-3 biological replicates, each time as a technical duplicate.

#### Generation of resistant mutants and whole genome sequencing

*Mtb* H37Rv ATCC27294 was grown to OD_650nm_ of 0.5 in 7H9/ADC/Tw, harvested and resuspended at 10^10^, 10^9^ and 10^8^ CFU/ml. Aliquots (0.1 ml) were spread on solid medium consisting of Middlebrook 7H11 (Becton Dickinson) supplemented with OADC [final concentration of 0.5% bovine serum albumin fraction V, 0.08% NaCl, 0.2% glucose, 0.2% glycerol, 0.06% oleic acid] containing 10X MIC concentration of TUN-8,8 or TUN-10,10. Colonies (1 for Tun-8,8 and 1 for **TUN-10,10**) that grew after 4-5 weeks of incubation were picked, grown up in 7H9/ADC/Tw liquid medium and resistance confirmed by MIC determination as described above. The observed frequency of resistance was 10^-9^ with 16-fold level of resistance recorded to the TUN-analogues. Genomic DNA was purified by the CTAB method (van Soolingen et al., 1991) and whole genome sequencing by Illumina MiSeq and assembly of paired-end reads by SPAdes performed as previously described (Weingarten et al., 2018). A resistant mutant raised against **TUN-10,10** had a SNP at position 842361 (C to G) resulting in an Ala to Gly mutation in Rv0751c. A resistant mutant raised to **TUN-8,8** had a T to A SNP at position 3336597 which is in the intergenic region between Rv2980 and Rv2981c.

#### Macromolecular incorporation assay

*Mtb* H37Rv ATCC27294 was grown to OD_650nm_ of 0.6 in 7H9/ADC/Tw was treated at 37 °C under constant agitation with 20 μCi/mL of D[-6-^3^H]-glucosamine (American Radiolabeled Chemicals, Inc. 40 Ci/mmol) for 2 h followed by addition of compound (**TUN-8,8**, tunicamycin, meropenem/clavulanate or DMSO as vehicle control) at 1X or 10X MIC concentrations. MIC concentrations for controls were as follows: D-cycloserine 29 μM, meropenem 2 μM with clavulanate used at a fixed concentration of 100 μM, tunicamycin 1.1 μM. After 24 h and 48 h incubation, 100 μl of culture was precipitated with 100 μl of 20% TCA in a round bottom 96 well polystyrene (Nunclon) plate. Precipitates were aspirated with a cell harvester (Perkin Elmer) and transferred to 96 well filtermat. The precipitates on the GF/C filtermats (Perkin Elmer) were washed twice with 200 μl 10% TCA, and subsequently three times with 200 μl of 70% ethanol. The filtermat was removed, dried overnight and then soaked with 5 ml of Betaplate scintillation cocktail (Perkin Elmer) followed by mounting in a filtermat holder. It was counted by Microbeta^2^ microplate scintillation counter (Perkin Elmer).

#### GFP release assay

*Mtb*-GFP (*M. tuberculosis* H37Rv (ATCC 27294) transformed with the plasmid pMSP12::GFP [Addgene plasmid # 30167]) was grown to an OD_650nm_ of 0.2 in 7H9/ADC/Tw and split into 30 ml aliquots in 250 ml roller bottles. Drug or vehicle control was added to each aliquot and the culture returned to 37 °C in a rolling bottle incubator. At indicated time points, 1 ml culture was removed, cells harvested at 13,000 rpm for 10 minutes and 100 μl supernatant transferred in triplicates to black 96-well plates (Thermo Fisher Sientific, USA) and fluorescence recorded in a FLUOstar Optima plate reader (BMG Labtech) at 485 nm excitation/ 520 nm emission wavelengths.

#### BODIPY-Vancomycin staining of nascent peptidoglycan

*Mtb* H37Rv ATCC27294 was grown to OD_650nm_ of 0.2 in 7H9/ADC/Tw and 5 ml volumes treated for up to 48 h with tunicamycin, **TUN-8,8**, meropenem/clavulanate or DMSO after which BODIPY-Vancomycin was added to a final concentration of 1 μg/ml. After 18 h of incubation, 3 ml of the suspension was harvested by centrifugation and washed twice in 1 ml PBS. The cell pellet was resuspended in 100 μl of fixative (2.5% glutaraldehyde in 100mM sodium cacodylate buffer) and 10 μl spread on a poly-L- lysine coated microscope slide onto which was added 50 μl of mounting medium (ProLong Gold Antifade reagent; Thermo Fisher Scientific, USA) and a coverslip. The slides were visualized by confocal fluorescence microscopy (Excitation 485 nm, Emission 520 nm) after overnight incubation at 4 °C.

#### Microsomal stability assay protocol

NADPH-regenerating system was freshly prepared by combining (a) 37.2 mg glucose-6-phosphate into 1 ml of 100 mM potassium phosphate buffer, (b) 39.8 mg NADP into 1 ml of 100 mM potassium phosphate buffer, (c) 11.5 U glucose-6-phosphate dehydrogenase into 1 mL of 100 mM potassium phosphate buffer and (d) 26.8 mg MgCl_2_.6H_2_O into 1 ml of water. 271 μl phosphate buffer (100 mM, pH 7.4) and 18 μl of NADPH-regenerating system were added to each tube, which were kept on ice. Pooled mouse/human liver microsomes were removed from the -80 °C freezer and thawed. 8 μl of liver microsomes (protein content >20 mg/ml) were added to each tube. The tubes were removed from ice and placed in a 37 °C heating block. 3 μl of test compound (0.5 mM stock) was added (final concentration 5 μM). Control incubation was included for each compound where phosphate buffer was added instead of NADPH-regenerating system (minus NADPH). Verapamil was used as control in the assay. The reaction was quenched at selected time-points by the addition of 150 μl methanol containing 5 μM internal standard. The samples were removed from the heating block and centrifuged at 14,000 g at 4 °C for 10 min. The supernatants were analyzed on LC/MS under single ionization mode (SIM) and scan mode.

#### Testing of tunicamycin analogue efficacy against *Mtb* growing in infected macrophages

J774A.1 mouse macrophage cells were grown in J774 growth medium consisting of DMEM GlutaMAX (Gibco) supplemented with 10% fetal bovine serum, 20 mM HEPES + 0.5 mM sodium pyruvate, seeded in sterile tissue culture treated 24-well plates (Corning) at 2.5x10^5^ cells/well and allowed to attach for 24 h in J774 growth medium. *Mtb* H37Rv ATCC was grown to OD6_50nm_ of 0.2 in 7H9/ADC/Tw, harvested, resuspended in J774 growth medium, filtered through a 5 μm filter to ensure a single cell suspension and diluted to 2.5x10^7^ cells/ml. Cells were infected with 0.1 mL *Mtb* cell suspension at a multiplicity of infection (MOI) of 10:1 and allowed for 24 h. After infection, the medium was aspirated and the monolayer of cells washed twice with Dulbecco’s PBS and subsequently fed with 1 ml of J774 growth medium containing the compounds at the indicated concentrations in triplicate wells for each drug concentration and time point. Rifampicin was used as positive control and methanol and DMSO used for the negative controls. After 3 and 7 days of treatment, cells were lysed by 0.1% SDS, appropriate dilutions made in 7H9/ADC/Tw and plated in duplicate on solid medium consisting of Middlebrook 7H11 (Becton Dickinson) supplemented with OADC [final concentration of 0. 5% bovine serum albumin fraction V, 0.08% NaCl, 0.2% glucose, 0.2% glycerol, 0.06% oleic acid]. Colony counts were enumerated after 4 weeks of incubation.

#### Mouse pharmacokinetics

Test compound was dosed to sixteen female C57BL/6 mice by intraperitoneal injection in a volume of 0.6 ml. To reconstitute **TUN-8,8** for intraperitoneal injections, compound (1 mg) was dissolved in 0.1 ml ethanol and subsequently diluted with 0.9 ml 1% Tween 80 in Dulbecco’s PBS. The mixture was warmed to 37 °C, vortexed and sonicated followed by sterile filtration through a 0.2 μm filter. Blood samples were taken from the tail vein of the mice at pre-determined time intervals post-dose, and serum collected after 4 h on ice. 30 μl serum samples were prepared with 30 μl internal standard and 20 μl of water or spiked standard along with 240 μl of ACN/MeOH mix (3:1) to precipitate proteins. Samples were centrifuged 13 krpm for 5 minutes and 2 μl injected into LC-MS/MS system. Calibration standard was prepared 1 mg of **TUN-8,8** to 1 ml DMSO and successively diluted by a factor of 3. **TUN-9,9** IS solution was prepared to 200 μg/ml in DMSO.

LC-MS/MS was performed on an Agilent 1290 Infinity HPLC coupled to an Agilent 6460C triple quadrupole mass selective detector with electrospray ionization in positive mode. **TUN-8,8** and **TUN-9,9** (as an internal standard) were detected using their M+H precursors ions with 514.1 and 528.2 Da/z product ions produced in a collision cell using collision energy 12 V. Capillary voltage was 3000 V and ESI used jet stream technology with sheath voltage 2000 V. The column was an Agilent C18 Poroshell 120 2.7 μm with dimensions 2.1 x 50 mm at 40 °C. Mobile phase was water (A) and acetonitrile (B) each with 0.1% (v/v) formic acid. With flow rate 0.8 ml/min, a gradient of 25% B progressed to 95% B over 4 minutes, washed and re-equilibrated. Water for LC-MS/MS was purified by a Barnstead Diamond system to 18.2 MΩ-cm resistivity. Acetonitrile (ACN) and Methanol were HPLC grade from Fisher (Fairlawn NJ, USA) and formic acid was supplied from EMD Millipore Corp (Darmstadt, Germany). DMSO was ACS grade manufactured by Amresco LLC in Solon Ohio USA.

#### Mouse infection

Female C57Bl/6 mice were aerosol-infected with 100-200 colony forming units of *Mtb* H37Rv with implantation dose determined after 24 hours by plating of lung homogenates on Middlebrook 7H11/OADC agar. Nine days after infection, five mice were euthanized and lung and spleens harvested for bacterial burden enumeration by plating of organ homogenates on Middlebrook 7H11/OADC agar. The remaining mice were treated in groups of 10 as follows: (1) daily intraperitoneal injections of 0.2 ml 10% ethanol/0.9% Tween 80/Dulbecco’s PBS; (2) daily intraperitoneal injections of 10 mg/kg **TUN-8,8** in 0.2 ml 10% ethanol/0.9% Tween 80/Dulbecco’s PBS; (3) oral gavage of 10 mg/kg Rifampicin in in 0.1ml water. Mice were treated daily for 2 weeks after which mice were euthanized and organ homogenates in 7H9/ADC/Tw plated on Middlebrook 7H11/OADC agar for colony enumeration.

#### Preparation of figures

Figures were prepared using either PyMOL (Schrodinger, 2010) or UCSF-Chimera (Pettersen et al., 2004). Electrostatic surface potentials (Figure 2B) were calculated using the APBS plugin within PyMOL and the PDB2PQR server (Dolinsky et al., 2004). Hydrogens and missing sidechain atoms were added automatically to the refined X-ray structure using ICM-Pro (Molsoft LLC) prior to electrostatic surface calculations. All electrostatic surface potentials were visualized in UCSF-Chimera and colored between -10 (red) and +10 (blue) kT/e^-^.

### Quantification and Statistical Analysis

All values are the mean ± standard deviation. GraphPad Prism v7.02 was used to plot Michaelis-Menten curves using the least squares fitting method and calculate the v_max_ and K_m_ values.

### Data and Software Availability

The crystal structures presented in this paper have been deposited in the PDB with accession numbers: 5LEV, 5O5E, 6FM9 and 6FWZ.

#### Additional Resources

DPAGT1 target enabling package: http://www.thesgc.org/tep/DPAGT1

## Supplemental Information

Methods S1. PDF file containing methods for semi-synthetic synthesis of the TUN-X,X analogues and related compounds. Related to Figure 5

Document S1. Tables S1–S7, Related to Figures 1, 4, 5, 6, and 7
