# Supplementary material for: Structures of DPAGT1 Explain Glycosylation Disease Mechanisms and Advance TB Antibiotic Design
Source: Cell. 2018 Nov 1;175(4):1045–1058.e16. doi: 10.1016/j.cell.2018.10.037 (PMC6218659; doi:10.1016/j.cell.2018.10.037)

**SUPPLEMENTARY INFORMATION**

**SEMI SYNTHETIC SYNTHESIS**

**for**

**Structures of DPAGT1 explain glycosylation disease mechanisms and advance TB antibiotic design**

Index

**Chemical synthesis methods:**

Chemical Synthesis Methods 3

General considerations 3

Extraction of tunicamycin 5

Large scale fermentation of *Streptomyces chartreusis* cells 7

Octa-*O*-acetyl-tunicamycin (tunicamycin-8OAc) 7

Tri-*N*-(tert-butoxylcarbonyl)-octa-*O*-acetyl-tunicamycin (tunicamycin-8OAc-3Boc) 8

10',2"-Di-*N*-Boc-α-D-glucosamine-(1”-11')-tunicamyl uracil (TUN-Boc,Boc) 9

α-D-*N*-acetylglucosamine-(1”-11')-N-acetyl tunicamyl uracil (TUN-Ac,Ac) 10

α-D-glucosamine-(1”-11')-tunicamyl uracil dihydrochloride (TUN) 11

Di-*N*-citronoyl-tunicamycin (TUN-Cit,Cit) 11

Di-*N*-heptanoyl tunicamycin (TUN-7,7) 12

Di-*N*-octanoyl-tunicamycin (TUN-8,8*)* 13

Di-*N*-nonanoyl-tunicamycin (TUN-9,9) 13

Di-*N*-decanoyl-tunicamycin(TUN-10,10) 14

Di-*N*-undecanoyl-tunicamycin(TUN-11,11) 15

Di-*N*-dodecanoyl-tunicamycin (TUN-12,12) 15

Heptaacetyl-tunicamyl-uracil (3) 16

N-acetyl-tunicamyl-uracil (2) 17

*N*-Octanoyl-*N’*-acetyl tunicamycin (TUN-8,Ac) 18

Solubility study of TUN-8,8 19

Purity of TUN-8,8 for mice administration 20

1,3,4,6-Tetra-*O*-acetyl-*N*-acetyl-d-glucosamine (4) 21

*N*-acetyl-3,4,6-Tris-*O*-acetyl-1-(dibenzyl phosphate)-α-d-glucosamine (5) 22

Undecaprenol 22

Undecaprenyl phosphate bisammonium salt (Und-P) 23

*N*-Acetyl-3,4,6-tris-*O*-acetyl-1-[*P*’-(3Z,7Z,11Z,15Z,19Z,23Z,27Z,31E,35E,39E,43-undecamethyl-2,6,10,14,18,22,26,30,34,38,42-tetratetracontaundecaenyl) *P,P*'-dihydrogen diphosphate]-α-d-glucosamine diimidazolium salt (6) 24

*N*-Acetyl-1-[*P*’-(3Z,7Z,11Z,15Z,19Z,23Z,27Z,31E,35E,39E,43-undecamethyl-2,6,10,14,18,22,26,30,34,38,42-tetratetracontaundecaenyl) *P,P*'-dihydrogen diphosphate]-α-d-glucosamine diammonium salt (7) 25

2-Deoxy-2-[[(2,2,2-trichloroethoxy)carbonyl]amino]-3,4,6-triacetyl-1-(2,2,2-trichloroethanimidate)-α-d-glucopyranose (9) 25

l-Alanine-2-(phenylsulfonyl)ethyl ester (10) 26

Phenylmethyl-2-(acetylamino)-2-deoxy-4,6-*O*-(phenylmethylene)-α-d-glucopyranoside (11) 27

*N*-Acetyl-1-*O*-(phenylmethyl)-4,6-*O*-(phenylmethylene)-α-d-muramic acid (12) 28

Phenylmethyl-2-(acetylamino)-2-deoxy-3-*O*-[(1*R*)-1-methyl-2-[[(1*S*)-1-methyl-2-oxo-2-[2-(phenylsulfonyl)ethoxy]ethyl]amino]-2-oxoethyl]-4,6-*O*-[(*R*)-phenylmethylene]-α-d-glucopyranoside (13) 28

Phenylmethyl-2-(acetylamino)-2-deoxy-3-*O*-[(1*R*)-1-methyl-2-[[(1*S*)-1-methyl-2-oxo-2-[2-(phenylsulfonyl)ethoxy]ethyl]amino]-2-oxoethyl]-6-*O*-(phenylmethyl)-α-d-glucopyranoside (14) 29

Boc-d-Ala-d-Ala-OMe (15) 30

Boc-Lys-d-Ala-d-Ala-OMe (16) 30

H-γ-d-Glu(α-OMe)-Lys(TFA)-d-Ala-d-Ala-OMe trifluoroacetate salt (17) 31

Phenylmethyl-2-(acetylamino)-2-deoxy-3-*O*-[(1*R*)-1-methyl-2-[[(1*S*)-1-methyl-2-oxo-2-[2-(phenylsulfonyl)ethoxy]ethyl]amino]-2-oxoethyl]-6-*O*-(phenylmethyl)-4-*O*-[3,4,6-tri-*O*-acetyl-2-deoxy-2-[[(2,2,2-trichloroethoxy)carbonyl]amino]-β-d-glucopyranosyl]-α-d-glucopyranoside (18) 32

Phenylmethyl 2-(acetylamino)-2-deoxy-3-*O*-[(1*R*)-1-methyl-2-[[(1*S*)-1-methyl-2-oxo-2-[2-(phenylsulfonyl)ethoxy]ethyl]amino]-2-oxoethyl]-4-*O*-[3,4,6-tri-*O*-acetyl-2-(acetylamino)-2-deoxy-β-D-glucopyranosyl]-6-*O*-acetyl-α-d-glucopyranoside (19) 33

*N*-[*N*-Acetyl-6-*O*-acetyl-1-*O*-[bis(phenylmethoxy)phosphinyl]-4-*O*-[3,4,6-tri-*O*-acetyl-2-(acetylamino)-2-deoxy-β-d-glucopyranosyl]-α-muramoyl]-l-alanine-2-(phenylsulfonyl)ethyl ester (20) 34

*N*-[*N*-Acetyl-6-*O*-acetyl-1-*O*-[bis(phenylmethoxy)phosphinyl]-4-*O*-[3,4,6-tri-*O*-acetyl-2-(acetylamino)-2-deoxy-β-d-glucopyranosyl]-α-muramoyl]-l-alanyl-l-γ-glutamyl-*N*6-(2,2,2-trifluoroacetyl)-l-lysyl-d-alanyl-2,5-dimethyl ester (21) 35

Lipid II diammonium salt (22) 36

NMR spectra 38

#

# Chemical Methods

## General considerations

Proton nuclear magnetic resonance (δ_H_) spectra were recorded on a Bruker DPX 200 (200 MHz), Bruker DPX 400 (400 MHz), Bruker DQX 400 (400 MHz), or Bruker AVC 500 (500 MHz) or Bruker AV 700 (700 MHz) spectrometer. Carbon nuclear magnetic resonance spectra were recorded on a Bruker DQX 400 (100 MHz) or Bruker AVC 500 (125 MHz) with a ^13^C cryobprobe (125 MHz) AV 600 (151 MHz) with a ^13^C cryobprobe (151 MHz) or AV 700 (176 MHz) with a ^13^C cryobprobe (176 MHz). Spectra were assigned using a combination of ^1^H, ^13^C, HSQC, HMBC, COSY, and TOCSY. All chemical shifts were quoted on δ-scale in ppm, with residual solvent as internal standard. Coupling constants (*J*) are reported in hertz (Hz). Infrared spectra were recorded on a Bruker Tensor 27 Fourier Transform spectrophotometer recorded in wavenumbers (cm^-1^). Low-resolution mass spectra were recorded on a LCT Premier XE using electrospray ionization (ES). High-resolution mass spectra were recorded on a Bruker microTOF. Specific rotations were measured on Perkin Elmer 241 polarimeter with pathlength of 1.0 dm and concentration (*c*) in g/100 mL. Thin layer chromatography (TLC) was performed on Merck EMD Kieselgel 60F_254_ precoated aluminum backed plates. Reverse-phase thin layer chromatography (RF-TLC) was performed on Merck EMD Silica Gel RP-18 W F254s precoated glass backed plates. TLC and RF-TLC were visualized in combination of: 254/365 nm UV lamp; sulfuric acid (2 M in EtOH/Water 1:1); ninhydrin (2% ninhydrin in EtOH); aqueous KMnO_4_ (5% KMnO_4_ in 1 M NaOH); aqueous phosphomolybdinc acid/Ce(IV) (2.5% phosphomolybdic acid hydrate, 1% cerium(IV) sulfate hydrate, and 6% H_2_SO_4_); or ammonium molybdate3 (5% in 2M H_2_SO_4_). Flash chromatography was carried out with Fluka Kiegselgel 60 220-440 mesh silica gel. All solvents (analytical or HPLC) used were purchased from Sigma Aldrich, Fisher Scientific, or Rathburn. Anhydrous solvents were purchased from Sigma Aldrich and stored over molecular sieves (<0.005 % H_2_O). Petrol refers to the fraction of petroleum ether boiling point in the range of 40 – 60 °C. Analytical (Synergi™ 4 µm Hydro-RP 80A 100 x 4.60 mm) and preparative (Synergi™ 4 µm Hydro-RP 80A 100 x 21.20 mm) reversed phase C18 column for HPLC were obtained from Phenomenex. Brine refers to saturated solution of NaCl.

**Analytical and Preparative HPLC Method for tunicamycins-like compound:**

Analytical-scale HPLC analysis and preparative-scale HPLC purification were performed on an UltiMate 3000, and the resulting data was analysed using Chromeleon software.

*Analytical Scale Analysis*. Column: Phenomenex, Synergi 4u Hydro-RP 80Å 100 x 4.60 mm 4micron; Flow rate: 1mL/min; Solvent A: 5% ACN and 0.1% FA in H_2_O;

Solvent B: 0.1% FA in ACN; UV 260 nm.

Eluent gradient

Min. %B

1.000 0.0[%]

25.000 100.0 [%]

27.010 100.0 [%]

29.010 0.0 [%]

35.010 0.0 [%]

*Preparative Scale Purification*. Column: Phenomenex, Synergi 4u Hydro-RP 80Å 100 x 21.20 mm 4micron; Flow rate: 12mL/min; Solvent A: 5% ACN and 0.1% FA in H_2_O;

Solvent B: 0.1% FA in ACN; UV 260 nm.

Eluent gradient

Min. %B

1.000 0.0[%]

25.000 100.0 [%]

27.010 100.0 [%]

28.010 0.0 [%]

35.010 0.0 [%]

**Molecular weight for the extracted tunicamycin homologues used in this work:** An average molecular weight has been used in molar calculations involving the extracted tunicamycins. Naturally produced tunicamycin is a mixture of homologues (see above). An average molecular weight of 838 g mol^-1^ is used in calculations based on the common homologue carbon chain lengths of *n* = 8, 9, 10, 11 unless otherwise specified.

## Extraction of tunicamycins

Crude tunicamycin was isolated from a *S. chartreusis* NRRL3882 fermentation culture by methanol extraction . *S. chartreusis* spore stock (2 µL) was added to 50 mL of TYD media in a 250 mL spring coiled flask, and incubated at 28°C and 200 rpm in a New Brunswick Series 25 shaker. After 36 h, aliquots of this culture (12 × 2 mL) was added to 12 × 1 L of TYD media including 6 g of glucose and 0.3 g of MgCl_2_ in unbaffled 2 L conical flasks, which were subsequently incubated at 28°C and 200 rpm in a New Brunswick Series 25 shaker. After 7 days, cells and supernatant were separated via decantation and centrifugation at 8500 rpm (Beckman Coulter Avanti J-25). Tunicamycin was extracted from both the centrifuged cells and supernatant. Tunicamycin in the supernatant was isolated by hydrophobic interaction chromatography. Amberlite XAD-16 was first preconditioned by washing with MeOH (x 3) and then distilled water (x 2). This preconditioned resin (15 g/L) was then added to the resulting supernatant and stirred for 2 h. The magnetic stirrer was then turned off and the XAD-16 resin was allowed to settle to the bottom of the flask, after which the majority of the supernatant was decanted and the remaining supernatant was removed by filtration. The collected resin was washed with water (200 mL) for 15 min and filtered through filter paper, and then stirred sequentially in MeOH (600 mL, 15 min), iPrOH (600 mL, 15 min) and MeOH (600 mL, overnight). The organic fractions were combined and concentrated *in vacuo*. The concentrated tunicamycin solution was aliquoted into four Falcon tubes and the volume adjusted to 40 mL with 1 M HCl to precipitate tunicamycin. The insoluble precipitate was collected via centrifugation, re-dissolved in MeOH and then diluted with 400 mL of acetone. The acetone solution was kept at -20 ^o^C overnight and the precipitated crude tunicamycin collected by filtration. Tunicamycin was also extracted from the cell pellet. The pellet was stirred in 1 M aq. HCl (800 mL) for 30 min, after which the cells were collected by centrifugation at 9000 rpm (Beckman Coulter Avanti J-25). This process was repeated, after which the cell pellet was stirred in MeOH (400 mL) overnight. The cells were collected by filtration, resuspended in MeOH (400 mL) and stirred for a further 4 h. The MeOH fractions were combined, concentrated *in vacuo*, and tunicamycin precipitated with acetone (400 mL). Crude tunicamycin: TLC: R*_f_* 0.3 in water/isopropanol/ethyl acetate (W/*i*POH/EtOAc, 1:3:6); ^1^H NMR (400 MHz, CD_3_OD) δ ppm 0.89, 0.91 (2 x s, 2 x 3 H, -CH(*CH_3_*)_2_), 1.14 – 1.66 (m, n x CH_2_^fatty acid^), 1.95 (s, 3 H, -CH_3_^NHAc^), 3.36 – 4.05 (m, -CH_2_^sugar^, CH^sugar^), 4.10 (t, *J* = 9.30 Hz, 1 H, H-10’), 4.20 (t, *J*_2’,1’_ = 5.80 Hz, 1 H, H-2’), 4.59 (d, *J*_11’,10’_ = 8.9 Hz, 1 H, H-11’), 4.94 (d, *J* = 3.6 Hz, 1 H, H-1’’), 5.77 (d, *J*_5,6_ = 8.2 Hz, 1 H, H-5^uracil^), 5.95 (d, *J*_1’,2’_ = 5.5 Hz, 1 H, H-1’), 5.96 (d, *J*_HC=CH trans_ = 15.4 Hz, 1 H, = C*H*C(O)-), 6.84 (dt, *J*_HC=CH trans_ = 14.5 Hz, *J* = 7.85 Hz, 1 H, -CH_2_*H*C=), 7.94 (d, *J*_6,5_ = 8.2 Hz, 1 H, H-6^uracil^); LRMS *m/z* (ESI^+^): [(M + Na)^+^] = 839 (18%), 853 (100%), 867 (92%), 881 (30%); (ESI^-^): [(M + Cl)^-^] = 851 (20%), 865 (100%), 879 (94%), 893 (34%). Flanking peaks with mass ± 14 corresponded to 8 x CH_2_, 9 x CH_2_, 10 x CH_2_, and 11 x CH_2_. IR ν: 3325, 2925, 2360, 2342, 1665, 1376, 1234, 1093, 1025; LC/MS *m/z* (TOF MS ES^+^): 761, 775, 789, 803, 817, 831, 845, 859, 873, 887, 901.


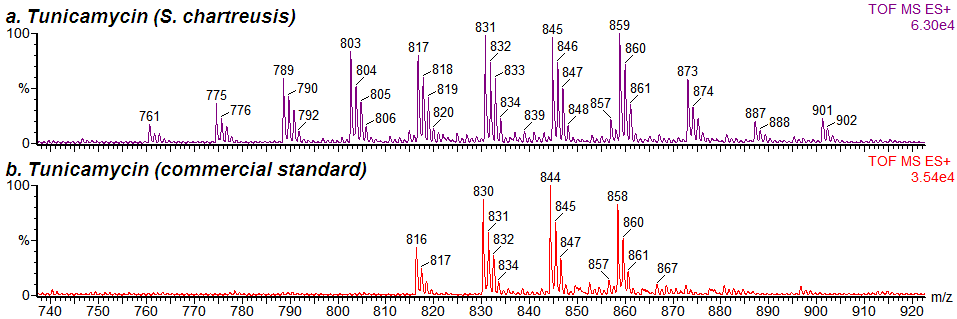


**SI, Figure 1. LC-MS Analysis of Tunicamycin Production by *S. chartreusis* NRRL 3882 by TOF-MS**: (a) crude tunicamycin extracted from the *S. chartreusis* culture. (b) commercial tunicamycin standard (Sigma Aldrich, retention time 14-19 min.).

**SI, Table 1. Tunicamycin extraction yield and purity.**

| ***S. chartreusis* strain** | **Culture Vol. (L)** | **tunicamycin isolated^a^ (mg)** | **Sample^a^**  **(mg/mL)** | **HPLC^b^**  **(mg/mL)** | **Purity^c^ (%)** | **tunicamycin**  **/Liter^d^ (mg/L)** |
| --- | --- | --- | --- | --- | --- | --- |
| NRRL3882 | 12 | 687.3 | 1.25 | 1.0296 | 82.4 | 47.2 |
| NRRL3882 | 24 | 1066.4 | 1.30 | 1.0201 | 78.5 | 34.9 |
| NRRL3882 | 12 | 1483.1 | 1.40 | 0.4718 | 33.7 | 41.7 |
| NRRL3882 | 11 | 891.1 | 1.40 | 0.7899 | 56.4 | 45.7 |

^a^Crude sample; ^b^Crude sample concentration injected into HPLC for analysis. tunicamycins dissolved in methanol;^c^Determined by HPLC, based on a standardised curve; ^d^Purity and Culture Vol. were taken in consideratin into the initial tunicamycins isolated. Average tunicamycins yield per liter of culture: 42 ± 5 mg

## Large scale fermentation of *Streptomyces chartreusis* cells

Sterile TYD media (2 g Tryptone, 2 g yeast extract, 6 g glucose and 30 mg MgCl_2_.6H_2_O per litre) was added to 4 x 500 mL conical spring flasks. Each flask was inoculated with 50μl of the *Streptomyces chartreusis* spore stock (~ 5x10^7^ spores) and incubated at 28 ^o^C with shaking on a rotary shaker (250 RPM) for 4 – 5 days. The flasks were then used to inoculate 90 L of TYD media in a Bioflow5000 fermenter at the University of East Anglia Fermentation Suite. Cells were fermented at 32 ^o^C with an air flow rate of 0.25 L/L/min for 5 – 7 days before being harvested. Tunicamycin was extracted from resulting mycelial cake as described above.

## Octa-*O*-acetyl-tunicamycin (tunicamycin-8OAc)

Crude tunicamycin (682 mg, 0.814 mmol) was dissolved in dry pyridine (5 mL) and Ac_2_O (3 mL). The reaction mixture was stirred for 18 h, concentrated *in vacuo* and purified by flash column chromatography (MeOH/DCM, 3:97) to afford the product as clear glass (782 mg, 0.667 mmol, 82 %); TLC: R*_f_* 0.4 in methanol/dichloromethane (MeOH/DCM, 3:97); ^1^H NMR (500 MHz, CD_3_OD) δ ppm 7.48 (d, *J*_6,5_ = 8.0 Hz, 1 H, H-6^uracil^), 6.83 (dt, *J*_HC=CH trans_ = 14.2 Hz, *J* = 7.3 Hz, 1 H, C=C*H*-CH_2_), 5.87 (d, *J*_HC=CH trans_ = 15.5 Hz, 1 H, C=C*H*-CO), 5.81 (d, *J*_1’,2’_ = 5.1 Hz, 1 H, H-1’), 5.75 (d, *J*_5,6_ = 8.0 Hz, 1 H, H-5^uracil^), 5.56 (dd, *J*_3’,2’_ = 6.1 Hz, *J*_3’,4’_ = 5.5 Hz, 1 H, H-3’), 5.51 (dd, *J*_2’,1’_ = *J*_2’,3’_ = 5.3 Hz, 1 H, H-2’), 5.26 (dd, *J*_3”,2”_ = 10.6 Hz, *J*_3”,4”_ = 9.9 Hz, 1 H, H-3”), 5.27 (ddd, *J*_5’,6’_ = 9.7 Hz, *J*_5’,4’_ = 6.8 Hz, *J* = 3.6 Hz, 1 H, H-5’), 5.11 (dd, *J*_8’,9’_ = 9.7 Hz, *J*_8’,7’_ = 7.3 Hz, 1 H, H-8’), 5.07 (app t, *J*_4”,5”_ = 11.3 Hz, *J*_4”,3”_ = 3.2 Hz, 1 H, H-4”), 5.03 (dd, *J*_9’,10’_ = 3.6 Hz, *J*_9’,8’_ = 3.2 Hz, 1 H, H-9’), 4.98 (d, *J*_1”,2”_ = 4.9 Hz, 1 H, H-1”), 4.75 (d, *J*_11’,10’_ = 8.4 Hz, 1 H, H-11’), 4.33 (dd, *J*_6a”,6b”_ =11.1 Hz, *J*_6”,5”_ = 3.9 Hz, 1 H, H-6”), 4.34 (dd, *J*_10’,9’_ = 4.6 Hz, *J*_10’,11’_ = 3.6 Hz, 1 H, H-10’), 4.32 (ddd, *J*_5”,4”_ = 10.4, *J*_5”,6”_ = 2.9 Hz, *J*_5”,6”_ = 2.2 Hz, 1 H, H-5”), 4.20 (dd, *J*_4’,3’_ = 7.7 Hz, *J*_4’,5’_ = 3.6 Hz, 1 H, H-4’), 4.19 (dd, *J*_2”,3”_ = 7.2 Hz, *J*_2”,1”_ = 3.1 Hz, 1 H, H-2”), 4.19 (dd, *J*_6a”,6b”_ = 14.2 Hz, *J*_6”,5”_ = 2.6 Hz, 1 H, H-6”), 3.92 (ddd, *J* = 9.4 Hz, *J*_7’,6’_ = 3.8 Hz, *J*_7’,8’_ = 3.1 Hz, 1 H, H-7’), 2.22 (s, 3 H, C*H*_3_^Ac^), 2.17 (m, 2 H, -C*H*_2_CH=C), 2.14 (s, 6 H, 2 x C*H*_3_^Ac^), 2.10 (s, 3 H, C*H*_3_^Ac^), 2.06 (m, 2 H, H-6’), 2.04, 2.03, 1.98, 1.95, 1.89 (5 x s, 5 x 3 H, 5 x C*H*_3_^Ac^), 1.78 (ddd, *J*_6b’,a’_ = 14.8 Hz, *J*_6’,5’_ = 8 Hz, *J*_6’,7’_ = 3.3 Hz, 1 H), 1.55 (spt, *J* = 6.7 Hz, 1 H, -C*H*(CH_3_)_2_), 1.46 (quin, *J* = 6.8 Hz, 2 H, -C*H*_2_CH_2_CH=C), 1.23 - 1.37 (m, 14 H, -C*H*_2_^acyl^), 1.18 (dt, *J* = 13.1, 7.0 Hz, 2 H, C*H*_2_CH(CH_3_)_2_), 0.91, 0.89 (2 x s, 2 x 3 H, -CH(C*H*_3_)_2_); ^13^C NMR (126 MHz, CD_3_OD) δ ppm 173.2, 172.4, 172.3, 172.3, 172.0, 171.7, 171.5, 171.3, 171.2 (C=O^Ac^, C=O^NHAc^), 169.5 (C=O^acyl^), 165.9 (C-4 C=O), 151.8 (C-2 C=O), 147.7 (C=*C*H-CH_2_), 143.4 (C-6^uracil^), 124.2 (C=*C*H-CO), 103.5 (C-5^uracil^), 101.6 (C-11’), 100.0 (C-1’’), 91.1 (C-1’), 84.1 (C-4’), 73.5 (C-2’), 72.2 (C-3”), 72.2 (C-9’), 71.7 (C-7’), 70.9 (C-3’), 70.8, 70.3 (C-5’, C-8’), 69.8 (C-5”), 69.7 (C-4”), 63.0 (C-6”), 52.6 (C-2”), 51.8 (C-10’), 40.3 (-*C*H_2_CH(CH_3_)_2_), 33.2 (-*C*H_2_CH=C), 33.1 (C-6’), 30.3 - 31.1 (5x-*C*H_2_^acyl^), 29.4 (-*C*H_2_CH_2_CH=C), 29.2 (-*C*H(CH_3_)_2_), 28.6 (-*C*H_2_^acyl^), 23.0, 23.1 (-CH(*C*H_3_)_2_), 22.9 (-*C*H_3_^NHAc^), 21.1 (-*C*H_3_^Ac^), 20.7 (2 x -*C*H_3_^Ac^), 20.6, 20.6, 20.6, 20.6, 20.3 (5 x -*C*H_3_^Ac^); IR ν: 2927, 2361, 2341, 1745, 1696, 1540, 1369, 1219, 1031; MS *m/z* (ESI^+^): 1203 [(M+Na)^+^, 100%]; (ESI^-^): 1179 [(M+Cl)^-^, 100%]. Flanking peaks with mass ± 14 corresponded to 8 x CH_2_, 9 x CH_2_, 10 x CH_2_, and 11 x CH_2_. Full assignment was not possible due to the presence homologues with mass ± 14.

## Tri-*N*-(tert-butoxylcarbonyl)-octa-*O*-acetyl-tunicamycin (tunicamycin-8OAc-3Boc)

In order to cleave the lipid chain, the *tert*-butoxylcarbonyl (Boc) protecting group was added to the secondary amides at positions 3, 10’, and 2” to afford the tri-N-Boc-octa-*O*-acetylated tunicamycins. Amide cleavage usually involves the use of a strong acid or base and high temperatures, but these harsh conditions would be unsuitable to use in the presence of the uridine moiety as they would degrade the tunicamycins. Several methodologies have been published on how to remove the highly stable and unreactive acetyl group. One of them is Kunieda’s mild *N*-bocylation methodology. Attachment of Boc group to the secondary amide increases the electrophillicity of carbonyl, allowing the acetyl group to be readily cleaved in the presence of a base.

**Tunicamycin-8OAc** (101 mg, 0.086 mmol) was dissolved in dry THF (1.5 mL) with the addition of 4-(dimethylamino)pyridine (10.5 mg, 0.086 mmol) and di-*tert*-butyl dicarbonate (187.7 mg, 0.86 mmol). The reaction mixture was heated to 60 °C with stirring for 4 h, and subsequently another portion of di-*tert*-butyl dicarbonate (187.7 mg, 0.86 mmol) was added to the reaction mixture, with stirring continued for an additional 2 h. After a total of 6 h, the reaction mixture was checked by TLC (EtOAc/Petrol, 6:4). This showed the formation of two products, **tunicamycin-8OAc-3Boc** (R*_f_* 0.3) and (**tunicamycin-8OAc-2Boc**) (R*_f_* 0.1). The reaction mixture was concentrated *in vacuo* and purified by flash column chromatography (EtOAc/Petrol, 6:4). **tunicamycin-8OAc-3Boc** (31.1 mg, 0.021 mmol, 25 %) was obtained as a yellow glass and **tunicamycin-8OAc-2Boc** (52.4 mg, 0.038 mmol, 44%) as a yellow oil; **tunicamycin-8OAc-3Boc**: TLC: R*_f_* 0.5 in ethyl acetate/petrol (EtOAc/Petrol, 6:4); ^1^H NMR (500 MHz, CDCl_3_) δ ppm 7.48 (d, *J*_6,5_ = 8.0 Hz, 1 H, H-6^uracil^), 6.89 (dt, *J*_HC=CH trans_ *=* 15.1 Hz, *J* = 6.9 Hz, 1 H, C=C*H*-CH_2_), 6.82 (dt, *J*_HC=CH trans_ = 14.5 Hz, *J* = 7.6 Hz, 1 H, C=C*H*-CH2), 6.39 (d, *J*_HC=CH trans_ = 15.1 Hz, 1 H, C=C*H*CO), 6.27 (d, *J*_HC=CH trans_ = 15.4 Hz, 1 H, C=C*H*-CO), 6.11 (m, 1 H, C-H^anomeric^), 5.85 (m, 1 H, CH^anomeric^, H-5^uracil^), 5.83 (d, *J* = 8.2 Hz, 1 H, H-5^uracil^), 5.61 (dd, *J* = 11.5 Hz, *J* = 3.3 Hz, 1 H), 5.53 (dd, *J* = 11.3 Hz, *J* = 3.5 Hz, 1 H), 5.49 (d, *J* = 8.2 Hz, 1 H), 5.43 (m, 1 H), 5.29 – 5.35 (m, 1 H), 5.09 – 5.25 (m, 4 H), 5.01 – 4.10 (m, 1 H), 4.99 (d, *J* = 9.1 Hz), 4.94 (dd, *J* = 11.5 Hz, *J* = 3.3 Hz, 1 H), 4.91 (s, 1 H), 4.52 – 4.60 (m, 1 H), 4.30 – 4.39 (m, 1 H), 4.17 (d, *J* = 10.4 Hz, *J* = 2.2 Hz, 1 H), 4.05 – 4.10 (m, 1 H), 3.76 (dd, *J* = 8.8 Hz, *J* = 3.5 Hz, 1 H), 3.68 (dd, *J* = 9.9 Hz, *J* = 2.0 Hz, 1 H), 2.34 (s, 1 H), 2.29 (s, 2 H, C*H*_3_^NHAc^), 2.27 (s, 1 H, C*H*_3_^NHAc^), 1.87 – 2.22 (m, 24H, 8 x CH_3_^Ac^), 1.59, 1.56, 1.55, 1.53, 1.52 (5 x s, 27 H, 9 x CH_3_^Boc^), 1.40 (m, 13 H), 1.08 – 1.18 (m, 2 H, CH_2_^acyl^), 0.86, 0.85 (2 x s, 2 x 3 H, C*H*_3_^acyl^); ^13^C NMR (126 MHz, CD_3_OD) δ ppm 177.4, 177.5, 170.8,170.7, 170.1, 169.9, 169.7, 169.6, 169.5, 169.4, 169.1 (C=O), 168.2 (C-4 C=O), 159.8 (C-2 C=O), 153.0, 152.9, 157.7, 152.7, 152.1, 148.3, 148.1, 147.3, 139.1, 139.0, 138.9 (C=*C*H-CH_2_, C-6^uracil^), 124.4, 123.9 (C=*C*H-CO), 103.5, 103.2 (C-1’’), 97.8, 87.7, 86.9, 87.0, 86.9, 86.3, 82.4, 82.3, 72.1, 70.4, 70.2, 70.1, 69.6, 69.5, 69.4, 69.2, 69.1, 68.8, 68.0, 67.9, 61.5, 61.4, 57.657.0, 54.8, 39.0, 38.5, 36.6, 34.3, 32.7, 32.5, 32.4, 31.9, 29.9, 29.6, 29.5, 29.4, 29.3, 29.2, 28.2, 28.0, 27.9, 27.8, 27.6, 27.4, 22.6, 20.9, 20.9, 20.7, 20.6, 20.5, 20.4 (C-1’), 84.1 (C-4’), 73.5 (C-2’), 72.2 (C-3”), 72.2 (C-9’), 71.7 (C-7’), 70.9 (C-3’), 70.8, 70.3 (C-5’, C-8’), 69.8 (C-5”), 69.7 (C-4”), 63.0 (C-6”), 52.6 (C-2”), 51.8 (C-10’), 40.3 (-*C*H_2_CH(CH_3_)_2_), 33.2 (-*C*H_2_CH=C), 33.1 (C-6’), 30.3 - 31.1 (5 x C, 5 x -*C*H_2_^acyl^), 29.4 (-*C*H_2_CH_2_CH=C), 29.2 (-*C*H(CH_3_)_2_), 28.6 (-*C*H_2_^acyl^), 23.0, 23.1 (2 x C, -CH(*C*H_3_)_2_), 22.9 (-*C*H_3_^NHAc^), 21.1 (-*C*H_3_^Ac^), 20.7 (2 x C, 2 x -*C*H_3_^Ac^), 20.6, 20.6, 20.6, 20.6, 20.3 (5 x C, 5 x -*C*H_3_^Ac^) IR ν: 2928, 2361, 2341, 1743, 1686, 1369, 1218, 1143, 1029; LRMS *m/z* (ESI^+^): 1503 [(M+Na)^+^, 100%]; (ESI^-^): 1515 [(M+Cl)^-^, 100%]. Flanking peaks with mass ± 14 corresponded to 8 x CH_2_, 9 x CH_2_, 10 x CH_2_, and 11 x CH_2_.

## 10',2"-Di-*N*-Boc-α-D-glucosamine-(1”-11')-tunicamyl uracil (TUN-Boc,Boc)

**Tunicamycin-8OAc-3Boc** (134 mg, 0.091 mmol) was dissolved in MeOH:H_2_O (v/v, 3:1) with the addition of TEA (25 equiv. 2.27 mmol, 317 µl). The reaction mixture was heated to 71 °C and reaction progress monitored by TLC (1:2:6, W/*i*PrOH/EtOAc). After 43 h the mixture was directly purified by preparative scale HPLC (retention time 9.5 min). Product containing fractions were pooled and lyophilized to afford **TUN-Boc,Boc** (36.4 mg, 0.047 mmol, 52%) as white amorphous powder; TLC: R*_f_* 0.3 in water/isopropanol/ethyl acetate (W/*i*POH/EtOAc, 1:2:6); R*_f_* = 0.3 (H_2_O/iPrOH/EtOAc, 1/2/7); [α]_D_^20^ = +54.9 ± 0.3 (c 1, MeOH); Mp (amorphous) 177.4−181.2 °C; ^1^H NMR (500 MHz, CD_3_OD) δ ppm 7.91 (d, *J* = 8.1 Hz, 1H, H-6), 5.93 (d, *J* = 5.9 Hz, 1H, H-1’), 5.75 (d, *J* = 8.1 Hz, 1H, H-5), 4.99 (s, 1H, H-1”), 4.70 (d, *J* = 7.9 Hz, 1H, H-11’), 4.24 – 4.16 (m, 2H, H-2’, H-3’), 4.05 – 3.97 (m, 2H, H-5’, H-5”), 3.86 (t, *J* = 3.3 Hz, 1H, H-4’), 3.81 (dd, *J* = 11.7, 1.8 Hz, 1H, H-6”), 3.77 – 3.65 (m, 3H, H-7’, H-9’, H-6”), 3.64 (d, *J* = 3.1 Hz, 1H, H-8’), 3.62 (d, *J* = 4.9 Hz, 2H, H-2”, H- 3”), 3.49 (t, *J* = 9.6 Hz, 1H, H-10’), 3.37 – 3.33 (m, 1H, H-4”), 2.12 – 2.05 (m, 1H, H-6’), 1.57 – 1.49 (m, 1H, H-6’), 1.47 (s, 9H, C*H*_3_), 1.45 (s, 9H, C*H*_3_); ^13^C NMR (126 MHz, CD_3_OD) δ ppm 166.2 (C-4), 158.7 (C=O^Boc^), 158.5 (C=O^Boc^), 152.6 (C-2), 142.8 (C-6), 103.1 (C-5), 101.4 (C-11’), 100.6 (C-1”), 89.7 (C-1’), 89.5 (C-4’), 80.7 (*C*-(CH_3_)_3_), 80.3 (*C*-(CH_3_)_3_), 75.5 (C-2’), 74.5 (C-5”), 73.6 (C-3”), 72.7, 72.6, 72.4 (C-7’, C-9’, C-4”), 72.3 (C-8’), 70.9 (C-3’), 68.4 (C-5’), 63.2 (C-6”), 56.6 (C-4”), 55.8 (C-10’), 35.9 (C-6’), 29.1((*C*H_3_)_3_), 28.8 ((*C*H_3_)_3_); IR (neat) ν: 3367 (N-H, O-H), 2979 (=C-H), 2930 (-C-H), 1684 (C=O); LRMS m/z (ESI^+^): 789 [(M+Na)^+^, 100%]; HRMS m/z (ESI^+^): calc. C_31_H_50_N_4_O_18_Na (M+Na)^+^ = 789.3012, found 789.3017.

## α-D-*N*-acetylglucosamine-(1”-11')-N-acetyl tunicamyl uracil (TUN-Ac,Ac)

**Tunicamycin-8OAc-3Boc** (127 mg, 0.086 mmol) was dissolved in dry MeOH (5 mL) and cooled to 0 °C. NaOMe was added to a final concentration of 0.01 M and reaction progress monitored by TLC (1:3:6, W/*i*POH/EtOAc). The reaction was neutralized after 4 h by addition of Dowex 50W X8 H^+^ resin in parts until pH 7. The mixture was then filtered, the resin washed with methanol and the combined organics concentrated *in vacuo*. The resulting solid was dissolved in TFA (1 mL) and stirred at RT for 1 h. The TFA was coevaporated with toluene and the crude material then redissolved in MeOH (5 mL) and Ac_2_O (1 mL). The reaction mixture was stirred at RT for 12 h, neutralized to pH 6-7 with Dowex Marathon A - OH resin and stirred for an additional 1 h. The reaction mixture was filtered, concentrated *in vacuo* and purified by flash column chromatography (W/*i*POH/EtOAc, 1:2:2) to afford **TUN-Ac,Ac** (13.1 mg, 0.020 mmol, 23%) as yellow glass; TLC**:** R*_f_* 0.3 (W/*i*POH/EtOAc, 1:2:2); [α]_D_^23^ = +50.7 (c = 0.7, H_2_O); ^1^H NMR (500 MHz, CD_3_OD) δ ppm 7.76 (d, *J*_6,5_ = 7.9 Hz, 1 H, H-6^uracil^), 5.84 (d, *J*_1’,2’_ = 7.9 Hz, 1 H, H-1’), 5.83 (d, *J*_5,6_ = 5.4 Hz, 1 H, H5^uracil^), 4.98 (d, *J*_1”,2”_ = 3.5 Hz, 1 H, H-1”), 4.58 (d, *J*_11’,10’_ = 8.5 Hz, 1 H, H-6^uracil^), 4.25 (dd, *J*_2’,1’_ = 5.4 Hz, *J*_2’,3’_ = 9.1 Hz, 1 H, H-2’), 4.22 (dd, *J*_3’,4’_ = 3.5 Hz, *J*_3’,2’_ = 5.7 Hz, 1 H, H-3’), 4.08 – 4.03 (m, 2 H, H-4’, H-5’), 3.87 (dd, *J*_10’,9’_ = 10.7 Hz, *J*_10’,11’_ = 8.5 Hz, 1 H, H-10’), 3.82 – 3.82 (m, 1 H, H-4”), 3.80 (dd, *J*_2”,1”_ = 3.8 Hz, *J*_2”,3”_ = 10.7, 1 H, H-2”), 3.77 (d, *J* = 10.1 Hz, 1 H, H-7’), 3.73 – 3.67 (m, 2 x 1 H, H-8’, H-6b”), 3.70 (dd, *J*_3”,2”_ = 10.7 Hz, *J*_3”,4”_ = 3.2 Hz, 1 H, H-3”), 3.68 – 3.41 (m, 2 H, H-6”a, H-9’), 3.44 (app t, *J*_5”,6a”_ = 9.8 Hz, 1 H, H-5”), 1.98, 1.94 (2 x s, 2 x 3H, 2 x -C*H*_3_^NHAc^), 1.94 (dd, *J*_6b’,6a’_ = 6.6 Hz, *J*_6b’,5’_ = 3.2 Hz, 1 H, H-6b’), 1.57 (app t, *J*_6a’,6b’_ = *J*_6a’,5’_ = 13.2 Hz, 1 H, H-6a’); ^13^C NMR (126 MHz, CD_3_OD) δ ppm 174.5, 174.1 (C=O^NHAc^), 166.2 (C-4, C=O)**,** 151.8 (C-2, C=O)**,** 141.9 (C-6^uracil^)**,** 102.5 (C-5^uracil^)**,** 99.8 (C-11’)**,** 98.3 (C-1”)**,** 88.5 (C-1’)**,** 87.2 (C-4’)**,** 73.4 (C-2’)**,** 72.6 (C-4”)**,** 71.3 (C-7’)**,** 71.1, 70.4, 69.8 (C-3”, C-8’, C-9’), 69.6 (C-5”), 68.9 (C-3’), 67.0 (C-5’), 60.4 (C-6”), 53.4 (C-2”), 52.8 (C-10’), 33.5 (C-6’), 22.2, 22.1 (2 x -C*H*_3_^NHAc^); IR (neat) ν: 3344, 2362, 2341, 2110, 1636, 1371, 1216; LRMS *m/z* (ESI^+^): 673.26 [(M+Na)^+^, 23%]; (ESI^-^): 649.23 [(M-H)^-^, 100%]; HRMS *m/z* (ESI+): calc. for C_25_H_38_N_4_NaO_16_ (M+Na)^+^ = 673.2175, found 673.2195.

## α-D-glucosamine-(1”-11')-tunicamyl uracil dihydrochloride (TUN)

To a solution of **TUN-Boc,Boc** (1.50 mg, 0.002 mmol) in DCM (120 μL) TFA (0.393 mmol 30 µL) was added. The reaction mixture was stirred at room temperature for 1 h, with reaction progress monitored by TLC (1:2:2, W/*i*POH/EtOAc). When the reaction was complete, the reaction mixture was concentrated *in vacuo*. The crude product was washed twice with H_2_O and DCM, the aqueous fraction collected and concentrated *in vacuo.* The dried crude product was then redissoved in 1 M HCl (1 mL), stirred for 1 h at room temperature and lyophilized to yield the product **TUN** (1.20 mg, 99%). [α]_D_^20^ = +60.1 ± 0.2 (c 1, H_2_O); ^1^H NMR (700 MHz, D_2_O) δ ppm 7.82 (d, *J* = 8.2 Hz, 1 H, H-6), 5.87 (d, *J* = 8.2 Hz, 1 H, H-5) 5.86 (d, *J* = 5.3 Hz, 1 H, H-1’), 5.53 (d, *J* = 3.4 Hz, 1 H, H-1”), 5.00 (d, *J* = 8.3 Hz, 1 H, H-11’), 4.31 - 4.26 (m, 2 H, H-2’, H-3’), 4.06 (td, *J* = 2.6, 11.1 Hz, 1 H, H-5’), 3.94 (dd, *J* = 3.3, 11.0 Hz, 1 H, H-9’), 3.92 - 3.87 (m, 3 H, H-7’, H-3”, H-5”), 3.84 (d, *J* = 3.2 Hz, 1 H, H-8’), 3.79 (dd, *J* = 3.8, 12.5 Hz, 1 H, H-6”), 3.70 (dd, *J* = 2.2, 12.4 Hz, 1 H, H6”), 3.57 (t, *J* = 9.6 Hz, 1 H, H-4”), 3.39 (dd, *J* = 3.5, 10.8 Hz, 1 H, H-2”), 3.31 (dd, *J* = 8.4, 11.0 Hz, 1 H, H-10’), 1.97 (ddd, *J* = 2.0, 10.4, 14.6 Hz, 1 H, H-6’), 1.70 - 1.64 (dtd, *J* = 2.8, 11.2 Hz, 1 H, H-6’); ^13^C NMR (176 MHz, CD_3_OD) δ ppm 166.17 (C-4), 151.8 (C-2), 142.0 (C-6), 102.4 (C-5), 99.4 (C-11’), 97.0 (C-1”), 88.7 (C-1’), 86.9 (C-4’), 73.4 (C-2’), 73.2 (C-5”), 71.8 (C-7”), 69.5 (C-8’), 69.3 (C-3”), 69.2 (C-9’), 68.9 (C-4’), 68.7 (C-3’), 66.9 (C-5’), 59.8 (C-6”), 53.8 (C-2”), 53.0 (C-10’), 33.3 (C-6’); IR (neat) ν: 3295 (N-H, O-H), 3057 (=C-H), 2922 (-C-H), 1673 (C=O), 1263 (C-N), 1109 (C-O), 1064 (C-O); LRMS m/z (ESI^+^): 567 [(M+H)^+^, 100%]; HRMS m/z (ESI^+^): calc. C_21_H_35_N_4_O_14_ (M+H)^+^ = 567.2144, found 567.2136.

**General protocol for preparing tunicamycin analogues**

HATU (2.5 equiv) was added to a solution of the appropriate carboxylic acid (2.5 equiv), EDC (2.5 equiv) and DIPEA (2.5 equiv) in dry DMF. The reaction mixture was stirred at RT for 10 min, followed by the addition of **TUN** (1 equiv) and DIPEA (2.5 equiv). The reaction mixture was stirred at RT for 2 ~ 4 h, diluted with a mixture of ACN/*i*POH/Water (1:1:1) and purified by preparative HPLC.

## Di-*N*-citronoyl-tunicamycin (TUN-Cit,Cit)

The product was purified by HPLC (0.1% FA and 5% - 100% acetonitrile gradient in 24 mins on C18 preparative column) and the desired product was eluted at 14 min. The lyophilised product was washed with DCM and MilliQ water and resulted in 5.1 mg of the final product, 54% yield. R*_f_* = 0.4 (1/3/6, H_2_O/*i*PrOH/EtOAc); [α]_D_^20^ = +45.2 ± 0.2 (c 0.4, MeOH); ^1^H NMR (500 MHz, CD_3_OD) δ ppm 7.91 (d, *J* = 8.1 Hz, 1H, H-6), 5.92 (d, *J* = 5.9 Hz, 1H, H-1’), 5.75 (d, *J* = 8.1 Hz, 1H, H-5), 5.11 (td, *J* = 7.0, 1.0 Hz, 2H, H-5”’), 4.96 (d, *J* = 3.4 Hz, 1H, H-1”), 4.58 (d, *J* = 8.5 Hz, 1H, H-11’), 4.24 – 4.15 (m, 2H, H-2’, H-3’), 4.06 – 3.95 (m, 3H, H-5’, H-10’, H-5”), 3.91 (dd, *J* = 10.6, 3.5 Hz, 1H, H-2”), 3.87 – 3.80 (m, 2H, H-4’, H-6”), 3.76 (appt dd, *J* = 9.5, 1.9 Hz, 1H, H-7’), 3.71 – 3.60 (m, 4H, H-8’, H-9’, H-3”, H-6”), 3.33 (appt d, *J* = 9.4 Hz, 1H, H-4”), 2.24 (m, 2H, H-1”’), 2.17 – 1.88 (m, 9H, H-6’, H-1”’, H-2”’, H-4”’), 1.67 (s, 6H, H-7”’), 1.61 (s, 6H, H-8”’), 1.52 (m, 1H, H-6’), 1.37 (m, 2H, H-3”’), 1.23 (m, 2H, H-3”’), 0.96 (d, *J* = 6.4 Hz, 6H, H-9”’); ^13^C NMR (126 MHz, CD_3_OD) δ ppm 176.6, 176.0 (N-C=O^aliphatic chain^), 166.2 (C-4), 152.6 (C-2), 142.8 (C-6), 132.3, 132.2 (C-6”’), 125.6, 125.5 (C-5”’), 103.0 (C-5), 101.6 (C-11’), 99.9 (C-1”), 89.8 (C-1’), 89.6 (C-4’), 75.5 (C-2’), 74.4 (C-5”), 73.1, 73.0 (C-8’, C-9’), 72.7 (C-4”), 72.5 (C-7’), 72.2 (C-3”), 70.8 (C-3’), 68.3 (C-5’), 63.2 (C-6”), 54.7 (C-2”), 54.3 (C-10’), 45.4, 44.9 (C-1”’), 38.6, 38.5 (C-3”’), 35.9 (C-6’), 31.8, 31.7 (C-2”’), 26.7, 26.6 (C-4”’), 25.9 (C-7”’), 19.6 (C-9”’), 17.8 (C-8”’); IR (neat) ν: 3291 (O-H), 2966 (C-H), 2928 (C-H), 1700 (C=O), 1638 (C=O),1541 (C=C), 1092 (C-N); LRMS m/z (ESI^-^): 915 [(M+FA-H)^-^, 100%]; HRMS m/z (ESI^-^): calc. C_41_H_65_N_4_O_16_ (M-H)^-^ = 869.4401, found 869.4407.

## Di-*N*-heptanoyl tunicamycin (TUN-7,7)

The product was purified by HPLC (0.1% FA and 5% - 100% acetonitrile gradient in 24 mins on C18 preparative column) and the desired product was eluted at 12 min. The lyophilised product was washed with DCM and MilliQ water and resulted in 4.8 mg of the final product, 39% yield. R*_f_* = 0.4 (1/3/6, H_2_O/*i*PrOH/EtOAc); [α]_D_^20^ = +26.8 ± 0.7 (c 0.2, MeOH); ^1^H NMR (500 MHz, CD_3_OD) δ ppm 7.92 (d, *J* = 8.1 Hz, 1H, H-6), 5.93 (d, *J* = 6.0 Hz, 1H, H-1’), 5.75 (d, *J* = 8.1 Hz, 1H, H-5), 4.94 (d, *J* = 3.4 Hz, 1H, H-1”), 4.61 (d, *J* = 8.5 Hz, 1H, H-11’), 4.24 – 4.16 (m, 2H, H-2’, H-3’), 4.06 – 3.99 (m, 2H, H-5’, H-5”), 3.95 (dd, *J* = 10.2, 8.6 Hz, 1H, H-10’), 3.90 (dd, *J* = 10.6, 3.5 Hz, 1H, H-2”), 3.87 – 3.81 (m, 2H, H-4’, H-6”), 3.77 (appt br d, *J* = 9.1 Hz, 1H, H-7’), 3.71 – 3.62 (m, 4H, H-8’, H9’, H-3”, H-6”), 3.34 (appt s, 1H, H-4”), 2.38 – 2.02 (m, 5H, 2 x CH_2_^fatty acyl^, H-6’), 1.69 – 1.49 (m, 5H, 2 x CH_2_^fatty acyl^, H-6’), 1.41 – 1.28 (m, 15H, C*H_2_*^fatty acyl^), 0.92 (t, *J* = 6.8 Hz, 6H, C*H_3_*^fatty acyl^); ^13^C NMR (126 MHz, CD_3_OD) δ ppm 177.2, 176.6 (N-C=O^fatty acyl^), 166.2, (C-4), 152.6, (C-2), 142.8, (C-6), 103.0, (C-5), 101.3, (C-11’), 99.9, (C-1”), 89.8, (C-1’), 89.6, (C-4’), 75.5 (C-2’), 74.4 (C-5”), 73.0 (C-8’, C-9’), 72.6 (C-4”), 72.5 (C-7’), 72.1 (C-3”), 70.9 (C-3’), 68.3 (C-5’), 63.3 (C-6”), 54.8 (C-2”), 54.5 (C-10’), 37.8, 37.2 (CO*C*H_2_-^fatty acyl^), 35.9 (C-6’), 32.9, 32.8, 30.2, 27.01, 26.8, 23.7 (*C*H_2_-^fatty acyl^), 14.4 (*C*H_3_^fatty acyl^); IR (neat) ν: 3305 (O-H), 2927 (C-H), 2856 (C-H), 1682 (C=O), 1645 (C=O),1552 (C=C), 1467 (CH2), 1376 (CH3), 1259 (C-O), 1094 (C-N); LRMS m/z (ESI^+^): 813 [(M+Na)^+^, 100%]; HRMS m/z (ESI^+^): calc. C_35_H_58_N_4_O_16_ (M+Na)^+^ = 813.3740, found 813.3708.

## Di-*N*-octanoyl-tunicamycin (TUN-8,8*)*

The product was purified by HPLC (0.1% FA and 5% - 100% acetonitrile gradient in 24 mins on C18 preparative column) and the desired product was eluted at 13.5 min. The lyophilised product was washed with DCM and MilliQ water and resulted in 2.5 mg of the final product, 63% yield. R*_f_* = 0.3 (1/3/6, H_2_O/*i*PrOH/EtOAc); [α]_D_^20^ = +57.4 ± 0.4 (c 0.2, MeOH); ^1^H NMR (500 MHz, CD_3_OD) δ ppm 7.91 (d, *J* = 8.1 Hz, 1H, H-6), 5.92 (d, *J* = 6.0 Hz, 1H, H-1’), 5.75 (d, *J* = 8.1 Hz, 1H, H-5), 4.94 (d, *J* = 3.4 Hz, 1H, H-1”), 4.60 (d, *J* = 8.5 Hz, 1H, H-11’), 4.24 – 4.15 (m, 2H, H-2’, H-3’), 4.06 – 3.98 (m, 2H, H-5’, H-5”), 3.95 (dd, *J* = 10.0, 8.6 Hz, 1H, H-10’), 3.90 (dd, *J* = 10.6, 3.4 Hz, 1H, H-2”), 3.87 – 3.80 (m, 2H, H-4’, H-6”), 3.76 (dd, *J* = 10.6, 1.6 Hz, 1H, H-7’), 3.70 – 3.61 (m, 4H, H-8’, H-9’, H-3”, H-6”), 3.34 (appt d, *J* = 4.0 Hz, 1H, H-4”), 2.38 – 2.14 (m, 4H, 2 x CH_2_^fatty acyl^), 2.10 (m, 1H, H-6’), 1.70 – 1.57 (m, 4H, 2 x CH_2_^fatty acyl^), 1.53 (ddd, *J* = 13.9, 11.4, 2.2 Hz, 1H, H-6’), 1.41 – 1.24 (appt br m, 16H, C*H_2_*^fatty acyl^), 0.91 (t, *J* = 6.9 Hz, 6H, C*H_3_*^fatty acyl^); ^13^C NMR (126 MHz, CD_3_OD) δ ppm 177.2, 176.6 (N-C=O^fatty acyl^), 166.1 (C-4), 152.6 (C-2), 142.8 (C-6), 103.1 (C-5), 101.3 (C-11’), 99.9 (C-1”), 89.8 (C-1’), 89.6 (C-4’), 75.5 (C-2’), 74.4 (C-5”), 73.0 (C-8’, C-9’), 72.6 (C-4”), 72.5 (C-7’), 72.1 (C-3”), 70.9 (C-3’), 68.3 (C-5’), 63.3 (C-6”), 54.8 (C-2”), 54.5 (C-10’), 37.8, 37.2 (CO*C*H_2_-^fatty acyl^), 35.9 (C-6’), 33.0, 33.0, 30.5, 30.3, 30.3, 27.0, 26.8, 23.7 (*C*H_2_-^fatty acyl^), 14.4 (*C*H_3_^fatty acyl^); IR (neat) ν: 3297 (O-H), 2957 (C-H), 2925 (C-H), 2853 (C-H), 1684 (C=O), 1644 (C=O), 1556 (C=C), 1469 (CH2), 1258 (C-O), 1091 (C-N), 1016 (=C-H); LRMS m/z (ESI^-^): 931 [(M+TFA-H)^-^, 100%]; HRMS m/z (ESI^+^): calc. C_37_H_62_N_4_O_16_Na (M+Na)^+^ = 841.4053, found 841.4045.

## Di-*N*-nonanoyl-tunicamycin (TUN-9,9)

The product was purified by HPLC (0.1% FA and 5% - 100% acetonitrile gradient in 24 mins on C18 preparative column) and the desired product was eluted at 15 min. The lyophilised product was washed with DCM and MilliQ water and resulted in 3.4 mg of the final product, 85% yield. R*_f_* = 0.4 (1/3/6, H_2_O/*i*PrOH/EtOAc); [α]_D_^20^ = +53.8 ± 1.2 (c 0.3, MeOH); ^1^H NMR (500 MHz, CD_3_OD) δ ppm 7.93 (d, *J* = 8.1 Hz, 1H, H-6), 5.95 (d, *J* = 5.9 Hz, 1H, H-1’), 5.77 (d, *J* = 8.1 Hz, 1H, H-5), 4.96 (d, *J* = 3.0 Hz, 1H, H-1”), 4.62 (d, *J* = 8.6 Hz, 1H, H-11’), 4.26 – 4.18 (m, 2H, H-2’, H-3’), 4.08 – 4.00 (m, 2H, H-5’, H-5”), 3.97 (t, *J* = 9.1 Hz, 1H, H-10’), 3.92 (dd, *J* = 10.7, 3.2 Hz, 1H, H-2”), 3.89 – 3.81 (m, 2H, H-4’, H-6”), 3.78 (appt br d, *J* = 9.8 Hz, 1H, H-7’), 3.73 – 3.63 (m, 4H, H-8’, H-9’, H-3”, H-6”), 3.36 (appt d, *J* = 4.2 Hz, 1H, H-4”), 2.41 – 2.16 (m, 4H, 2 x CH_2_^fatty acyl^), 2.12 (appt br t, *J* = 12.1 Hz, 1H, H-6’), 1.71 – 1.59 (m, *J* = 6.7 Hz, 4H, 2 x CH_2_^fatty acyl^), 1.55 (appt br t, *J* = 12.6 Hz, 1H, H-6’), 1.34 (s, 20H, C*H_2_*^fatty acyl^), 0.93 (t, *J* = 6.5 Hz, 6H, C*H_3_*^fatty acyl^); ^13^C NMR (126 MHz, CD_3_OD) δ ppm 177.2, 176.6 (N-C=O^fatty acyl^), 166.1 (C-4), 152.6 (C-2), 142.8 (C-6), 103.0 (C-5), 101.3 (C-11’), 99.9 (C-1”), 89.8 (C-1’), 89.6 (C-4’), 75.5 (C-2’), 74.4 (C-5”), 73.1, 73.0 (C-8’, C-9’), 72.6 (C-4”), 72.5 (C-7’), 72.1 (C-3”), 70.9 (C-3’), 68.3 (C-5’), 63.3 (C-6”), 54.7 (C-2”), 54.5 (C-10’), 37.8, 37.2 (CO*C*H_2_-^fatty acyl^), 35.9 (C-6’), 33.1, 33.0, 30.6, 30.5, 30.4, 30.3, 27.0, 26.8, 23.8 (*C*H_2_-^fatty acyl^), 14.5 (*C*H_3_^fatty acyl^); IR (neat) ν: 3301 (O-H), 2923 (C-H), 2852 (CH), 1738 (C=O), 1646 (C=O), 1544 (C=C), 1420 (CH_2_), 1366 (CH_3_), 1229 (C-O), 1092 (CN), 1015 (=C-H); LRMS m/z (ESI^-^): 891 [(M+FA-H)^-^, 100%]; HRMS m/z (ESI^-^): calc. C_39_H_65_N_4_O_16_ (M-H)^-^ = 845.4401, found 845.4412.

## Di-*N*-decanoyl-tunicamycin (TUN-10,10)

The product was purified by HPLC (0.1% FA and 5% - 100% acetonitrile gradient in 24 mins on C18 preparative column) and the desired product was eluted at 16.5 min. The lyophilised product was washed with DCM and MilliQ water and resulted in 3 mg of the final product, 31% yield. R*_f_* = 0.4 (1/3/6, H_2_O/*i*PrOH/EtOAc); [α]_D_^20^ = +38.0 ± 0.6 (c 0.3, MeOH); ^1^H NMR (500 MHz, CD_3_OD) δ ppm 7.91 (d, *J* = 8.1 Hz, 1H, H-6), 5.92 (d, *J* = 6.0 Hz, 1H, H-1’), 5.75 (d, *J* = 8.1 Hz, 1H, H-5), 4.93 (d, *J* = 3.4 Hz, 1H, H-1”), 4.59 (d, *J* = 8.5 Hz, 1H, H-11’), 4.23 – 4.16 (m, 2H, H-2’, H-3’), 3.97 – 3.92 (m, 2H, H-5’, H-5”), 3.90 (appt t, *J* = 8.5 Hz, 1H, H-10’), 3.84 (dd, *J* = 10.6, 3.4 Hz, 1H, H-2”), 3.87 – 3.80 (m, 2H, H-4’, H-6”), 3.76 (appt br dd, *J* = 10.7, 1.7 Hz, 1H, H-7’), 3.71 – 3.61 (m, 4H, H-8’, H-9’, H-3”, H-6”), 3.33 (appt d, *J* = 5.8 Hz, 1H, H-4”), 2.38 – 2.02 (m, 4H, 2 x CH_2_^fatty acyl^), 2.10 (m, 1H, H-6’), 1.69 – 1.49 (m, 4H, 2 x CH_2_^fatty acyl^), 1.55 (m, 1H, H-6’), 1.30 (s, 24H, C*H_2_*^fatty acyl^), 0.90 (t, *J* = 6.9 Hz, 6H, C*H_3_*^fatty acyl^); ^13^C NMR (126 MHz, CD_3_OD) δ ppm 177.17, 176.6 (NC=O^fatty acyl^), 166.2 (C-4), 152.6 (C-2), 142.8 (C-6), 103.1 (C-5), 101.3 (C-11’), 100.0 (C-1”), 89.8 (C-1’), 89.6 (C-4’), 75.5 (C-2’), 74.4 (C-5”), 73.1, 73.0 (C-8’, C-9’), 72.6 (C-4”), 72.5 (C-7’), 72.1 (C-3”), 70.9 (C-3’), 68.3 (C-5’), 63.3 (C-6”), 54.7 (C-2”), 54.5 (C-10’), 37.8, 37.2 (CO*C*H_2_-^fatty acyl^), 35.9 (C-6’), 33.1, 30.9, 30.8, 30.7, 30.6, 30.5, 27.0, 26.8, 23.8 (*C*H_2_-^fatty acyl^), 14.5 (*C*H_3_^fatty acyl^); IR (neat) ν: 3305 (O-H), 2922 (C-H), 2851 (C-H), 1683 (C=O), 1645 (C=O),1551 (C=C), 1468 (CH_2_), 1260 (C-O), 1094 (C-N), 1017 (=C-H); LRMS m/z (ESI^-^): 919 [(M+FA-H)^-^, 100%]; HRMS m/z (ESI^+^): calc. C_41_H_70_N_4_O_16_Na (M+Na)^+^ = 897.4679, found 897.4666.

## Di-*N*-undecanoyl-tunicamycin (TUN-11,11)

The product was purified by HPLC (0.1% FA and 5% - 100% acetonitrile gradient in 24 mins on C18 preparative column) and the desired product was eluted at 18 min. The lyophilised product was washed with DCM and MilliQ water and resulted in 3mg of the final product, 30% yield. R*_f_* = 0.4 (1/3/6, H_2_O/*i*PrOH/EtOAc); [α]_D_^20^ = +30.9 ± 0.4 (c 0.25, MeOH); ^1^H NMR (500 MHz, CD_3_OD) δ ppm 7.91 (d, *J* = 8.1 Hz, 1H, H-6), 5.92 (d, *J* = 6.0 Hz, 1H, H-1’), 5.75 (d, *J* = 8.1 Hz, 1H, H-5), 4.93 (d, *J* = 3.4 Hz, 1H, H-1”), 4.60 (d, *J* = 8.5 Hz, 1H, H-11’), 4.23 – 4.15 (m, 2H, H-2’, H-3’), 4.05 – 3.98 (m, 2H, H-5’, H-5”), 3.95 (m, 1H, H-10’), 3.90 (dd, *J* = 10.6, 3.4 Hz, 1H, H-2”), 3.86 – 3.80 (m, 2H, H-4’, H-6”), 3.76 (appt br dd, *J* = 10.7, 1.8 Hz, 1H, H-7’), 3.71 – 3.61 (m, 4H, H-8’, H-9’, H-3”, H-6”), 3.34 (m, 1H, H-4”), 2.37 – 2.14 (m, 4H, 2 x CH_2_^fatty acyl^), 2.13 – 2.05 (m, 1H, H-6’), 1.68 – 1.56 (m, 4H, 2 x CH_2_^fatty acyl^), 1.57 – 1.49 (m, 1H, H-6’), 1.30 (appt br s, 32H, C*H_2_*^fatty acyl^), 0.90 (t, *J* = 6.9 Hz, 6H, C*H_3_*^fatty acyl^); ^13^C NMR (126 MHz, CD_3_OD) δ ppm 177.2, 176.6, (NC=O^fatty acyl^), 166.2 (C-4), 152.7 (C-2), 142.8 (C-6), 103.0 (C-5), 101.3 (C-11’), 100.0 (C-1”), 89.8 (C-1’), 89.6 (C-4’), 75.5 (C-2’), 74.4 (C-5”), 73.1, 73.0 (C-8’, C-9’), 72.6 (C-4”), 72.5 (C-7’), 72.1 (C-3”), 70.9 (C-3’), 68.3 (C-5’), 63.3 (C-6”), 54.8 (C-2”), 54.5 (C-10’), 37.8, 37.2 (CO*C*H_2_-^fatty acyl^), 35.9 (C-6’), 33.1, 30.9, 30.8, 30.7, 30.6, 30.5, 30.4, 27.0, 26.9, 23.8 (*C*H_2_-^fatty acyl^), 14.5 (*C*H_3_^fatty acyl^); IR (neat) ν: 3297 (O-H), 2956 (C-H), 2921 (C-H), 2852 (C-H), 1738 (C=O), 1719 (C=O), 1680 (C=C), 1645 (C=O), 1550 (N-H), 1468 (CH_2_), 1366 (CH_3_), 1229 (C-O-C), 1217 (C-OH), 1260 (C-O), 1092 (C-N), 1017 (=C-H); LRMS m/z (ESI^-^): 947 [(M+FA-H)^-^, 100%]; HRMS m/z (ESI^-^): calc. C_43_H_73_N_4_O_16_ (M-H)^-^ = 901.5027, found 901.5015.

## Di-*N*-dodecanoyl-tunicamycin (TUN-12,12)

The product was purified by HPLC (0.1% FA and 5% - 100% acetonitrile gradient in 24 mins on C18 preparative column) and the desired product was eluted at 20.5 min. The lyophilised product was washed with DCM and MilliQ water and resulted in 3mg of the final product, 29% yield. R*_f_* = 0.4 (1/3/6, H_2_O/*i*PrOH/EtOAc); [α]_D_^20^ = +15.9 ± 0.4 (c 0.25, MeOH); ^1^H NMR (500 MHz, CD_3_OD) δ 7.92 (d, *J* = 8.1 Hz, 1H, H-6), 5.93 (d, *J* = 5.9 Hz, 1H, H-1’), 5.76 (d, *J* = 8.1 Hz, 1H, H-5), 4.94 (d, *J* = 3.5 Hz, 1H, H-1”), 4.60 (d, *J* = 8.5 Hz, 1H, H-11’), 4.24 – 4.17 (m, 2H, H-2’, H-3’), 4.08 – 3.99 (m, 2H, H-5’, H-5”), 3.96 (m, *J* = 8.6 Hz,1H, H-10’), 3.91 (dd, *J* = 10.6, 3.4 Hz, 1H, H-2”), 3.88 – 3.80 (m, 2H, H-4’, H-6”), 3.77 (appt br dd, *J* = 11.1, 1.8 Hz, 1H, H-7’), 3.72 – 3.61 (m, 4H, H-8’, H-9’, H-3”, H-6”), 2.39 – 2.15 (m, 4H, 2 x CH_2_^fatty acyl^), 2.11 (m, 1H, H-6’), 1.70 – 1.57 (m, 4H, 2 x CH_2_^fatty acyl^), 1.54 (m, 1H, H-6’), 1.40-1.28 (appt broad m, 32H, C*H_2_*^fatty acyl^), 0.91 (t, *J* = 6.9 Hz, 6H, C*H_3_*^fatty acyl^); ^13^C NMR (126 MHz, CD_3_OD) δ ppm 177.17, 176.57 (N-C=O^fatty acyl^), 166.16 (C-4), 152.63 (C-2), 142.76 (C-6), 103.06 (C-5), 101.35 (C-11’), 100.04 (C-1”), 89.77 (C-1’), 89.62 (C-4’), 75.52 (C-2’), 74.37 (C-5”), 73.08, 73.04 (C-8’, C-9’), 72.60 (C-4”), 72.49 (C-7’), 72.10 (C-3”), 70.90 (C-3’), 68.33 (C-5’), 63.27 (C-6”), 54.76 (C-2”), 54.45 (C-10’), 37.82, 37.19 (CO*C*H_2_-^fatty acyl^), 35.94 (C-6’), 30.87, 30.83, 30.79, 30.77, 30.68, 30.65, 30.63, 30.55, 27.05, 26.83, 23.79 (*C*H_2_-^fatty acyl^), 14.48 (*C*H_3_^fatty acyl^); IR (neat) ν: 3297 (O-H), 2956 (C-H), 2921 (C-H), 2851 (C-H), 1682 (C=O), 1646 (C=O), 1556 (C=C), 1468 (CH2), 1260 (C-O), 1092 (C-N), 1016 (=C-H); LRMS m/z (ESI^-^): 976 [(M+FA-H)^-^, 100%]; HRMS m/z (ESI^+^): calc. C_45_H_78_N_4_O_16_Na (M+Na)^+^ = 953.5305, found 953.5334.

## Heptaacetyl-tunicamyl-uracil (3)

Crude tunicamycin (183 mg, 0.218 mmol) was suspended in 3 M aq. HCl (2 mL) and stirred under reflux at 105 °C for 135 min. The solvent was then co-evaporated with toluene *in vacuo*. The resulting residue was re-dissolved in dry pyridine (3 mL) and Ac_2_O (2 mL) and stirred for 18 h at RT. The reaction mixture was then concentrated *in vacuo* and purified by flash column chromatography (MeOH/EtOAc, 1:19) to afford Heptaacetyl-tunicamyl-uracil **3** (98.4 mg, 0.141 mmol, 64 %); TLC: R*_f_* 0.3 in methanol/ethyl acetate (MeOH/EtOAc, 1:19); ^1^H NMR (500 MHz, CDCl_3_) δ ppm 8.53 (d, *J* = 1.00 Hz, 1 H, N-H^uracil,β^), 8.43 (d, *J* = 0.95 Hz, 1 H, N-H^uracil,α^), 7.22 (d, *J*_6,5_ = 8.2 Hz, 1 H, H-6^uracil,α^), 7.19 (d, *J*_6,5_ = 8.2 Hz, 1 H, H-6^uracil,β^), 6.13 (d, *J*_11’,10’_ = 3.5 Hz, 1 H, H-11’^α^), 5.90 (d, *J*_1’,2’_ = 5.4 Hz, 1 H, H-1’^α^), 5.83 (d, *J*_1’,2’_ = 3.8 Hz, 1 H, H-1’^β^), 5.80 (d, *J* = 2.2 Hz, 1 H, H-5^uracil,α^), 5.78 (dd, *J* = 2.1 Hz, *J*_5,6_ = 8.0, 1 H, H-5^uracil,β^), 5.64 (d, *J*_11’,10’_ = 8.8 Hz, 1 H, H-11’^β^), 5.55 (d, *J*_N-H,10’_ = 9.5 Hz, 1 H, N-H^Ac,β^), 5.44 (d, *J*_N-H,10’_ = 9.1 Hz, 1 H, N-H^Ac,α^), 5.42 (dd, *J*_3’,4’_ = 5.4 Hz, *J*_3’,2’_ = 10.4 Hz, 1 H, H-3’^β^), 5.36 (dd, *J*_3’,4’_ = 5.0 Hz, *J*_3’,2’_ = 5.9 Hz, 1 H, H-3’^α^), 5.33 (app t, *J*_2’,1’_ = *J_2’_*_,3’_ = 5.7 Hz, 1H, H-2’^α^), 5.30 (app t, *J*_2’,1’_ = *J*_2’,3’_ = 6.0 Hz, 1 H, H-2’^β^), 5.25 (d, *J*_9’,8’_ = *J*_9’,10’_ = 2.8 Hz, 1 H, H-9’^α^), 5.22 (dd, *J*_8’,7’_ = 3.2 Hz, *J*_8’,9’_ = 6.6 Hz, 1 H, H-8’^β^), 5.19 (dd, *J*_5’,4’_ = 1.9 Hz, *J*_5’,6’_ = 3.5 Hz, 1H, H-5’^β^), 5.19 (dd, *J*_8’,7’_ = 1.5 Hz, *J*_8’,9_ = 3.5 Hz, 1 H, H-8’^α^), 5.12 (ddd, *J* = 2.8 Hz, *J*_5’,4’_ = 5.0 Hz, *J*_5’,6’_ = 7.9 Hz, 1 H, H-5’^α^), 5.08 (dd, *J*_9’,8’_ = 3.50 Hz, *J*_9’,10’_ = 11.3 Hz, 1 H, H-9’^β^), 4.72 (ddd, *J*_10’,11’_ = 4.1 Hz, *J*_10’,N-H_ = 9.5 Hz, *J*_10’,9’_ = 11.4 Hz, 1 H, H-10’^α^), 4.42 (ddd, *J*_10’9’_ = 7.6 Hz, *J*_10’,NH’_ = 9.5 Hz, *J*_10’,9’_ = 11.3 Hz, 1 H, H-10’^β^), 4.13 (app t, *J*_4’,5’_ = *J*_4’,3’_ = 4.7 Hz, 1 H, H-4’^β^), 4.09 (app t, *J*_4’,5’_ = *J*_4’,3’_ = 4.7 Hz, 1 H, H-4’^α^), 4.05 - 4.07 (m, 1 H, H-7’^α^), 3.90 (dd, *J*_7’,8’_ = 2.5 Hz, *J*_7’,6’_ = 9.7 Hz, 1 H, H-7’^β^), 2.20 (s, 3 H, -CH_3_^Ac,α^), 2.20 (s, 3 H, -CH_3_^Ac,β^), 2.18, 2.13 (2 x s, 2 x 3H, 2 x -CH_3_^Ac,α^), 2.13, 2.12, 2.11, 2.11 (4 x s, 4 x 3 H, 4 x -CH_3_^Ac,β^), 2.09 (2 x s, 2 x 3 H, 2 x -CH_3_^Ac,α^), 2.04 (s, 3 H, -CH_3_^Ac,α^), 2.02 (s, 3 H, -CH_3_^Ac,β^), 1.99 - 2.01 (m, 1 H, H-6’^β^), 1.97 - 1.99 (m, 1 H, H-6’^α^), 1.96 (s, 3 H, -CH_3_^NHAc,α^), 1.94 (s, 3 H, -CH_3_^NHAc,β^), 1.74 (ddd, *J*_6’,7’_ = 3.5 Hz, *J*_6’,5’_ = 6.9 Hz, *J*_6’a,6b’_ = 14.9 Hz, 1 H, H-6’^β^), 1.57 (ddd, *J*_6’,7’_ = 1.9 Hz, *J*_6’,5’_ = 8.2 Hz, *J*_6’a,6’b_ = 16.7 Hz, 1 H, H-6’^α^); ^13^C NMR (126 MHz, CDCl_3_) δ ppm 173.6, 171.2, 170.7, 170.6, 170.6, 170.3, 170.1, 170.0, 169.7, 169.6, 169.4, 169.3, 169.3, 169.1 (C=O^NHAc,α,β^, C=O^Ac,α,β^), 162.5 (C-4 C=O^β^), 162.5 (C-4 C=O^α^), 149.8 (C-2 C=O^α^), 149.8 (C-2 C=O^β^), 140.1 (C-6^uracil,β^), 139.8 (C-6^uracil,α^), 103.4 (C-5^uracil,α^), 103.3 (C-5^uracil,β^), 93.0 (C-11’^β^), 91.1 (C-11’^α^), 88.7 (C-1’^β^), 88.1 (C-1’^α^), 82.5 (C-4’^β^), 82.5 (C-4’^α^), 72.4 (C-2’^α^), 72.4 (C-2’^β^), 71.0 (C-7’^β^), 70.5 (C-9’^β^), 69.6, 69.6, 69.3, 68.7, 68.1 (5 x s, 7 x C, C-3’^α^, C-3’^β^, C-5’^α^, C-5’^β^, C-8’^α^, C-8’^β^, C-9’^α^), 49.6 (C-10’^β^), 46.7 (C-10’^α^), 32.5 (C-6’^α^), 31.5 (C-6’^β^), 23.3 (-CH_3_^NHAc,β^), 23.2 (-CH_3_^NHAc,α^), 20.3 – 21.0 (CH_3_^Ac,α,β^); IR: 3370, 1736, 1710, 1697, 1651, 1635, 1540, 1520, 1370; LRMS *m/z* (ESI^+^): 722 [(M+Na)^+^, 100%]; (ESI^-^): 734 [(M+Cl)^-^, 100%]. HRMS *m/z* (ESI^+^): calc. for C_29_H_37_N_3_NaO_17_ (M+Na)^+^ = 722.2015, found 722.2023.

## N-acetyl-tunicamyl-uracil (2)

Heptaacetyl-tunicamyl-uracil **3** (41.4 mg, 0.059 mmol) was dissolved in dry MeOH (5 mL) and cooled to 0 °C. NaOMe was added to a final concentration of 0.01 M and the reaction mixture stirred for 3 h. The reaction mixture was then neutralized with Dowex 50W X8 H^+^ resin, filtered and concentrated *in vacuo*. Purification by flash column chromatography (W/*i*POH/EtOAc, 1:2:2) afforded the product **2** (25.9 mg, 0.058 mmol, 98 %); TLC: R*_f_* 0.5, 0.6 (W/*i*POH/EtOAc, 1:2:2); [α]_D_^23^ = +12 (c 1, H_2_O); ^1^H NMR (500 MHz, D_2_O) δ ppm 7.78 (d, *J*_6,5_ = 8.2 Hz, 1 Hα, H-6^uracil,α^), 7.76 (d, *J*_6,5_ = 8.2 Hz, 1 H^β^, H-6^uracil,β^), 5.87 (m, 1 H^α^ + 1 H^β^, H-1’^α^, H-1’^β^), 5.84 (d, *J*_5,6_ = 8.2 Hz, 1 H^α^, H-5^uracil,α^), 5.82 (d, *J*_5,6_ = 8.2 Hz, 1 H^β^, H-5^uracil,β^), 5.13 (d, *J*_11’,10’_ = 3.8 Hz, 1 H^α^, H-11’^α^), 4.58 (d, *J*_11’,10’_ = 8.5 Hz, 1 H^β^, H-11’^β^), 4.23 - 4.27 (m, 2 H^α^ + 2H^β^, H-2’^α^, H-2’^β^, H-3’^α^, H-3’^β^), 4.19 (d, *J* = 9.1 Hz, 1 H^α^, H-7’^α^), 4.04 (dd, *J*_10’,9’_ = 11.0 Hz, *J*_10’,11_ = 3.8 Hz, 1 H^α^, H-10’^α^), 4.00 (dt, *J* = 10.7 Hz, *J* = 3.2 Hz, 1 H^β^, H-5’^β^), 3.92 - 3.97 (m, 2 H^α^ + 1 H^β^, H-4’^α^, H-4’^β^, H-5’^α^), 3.88 (dd, *J*_9’,10’_ = 11.4 Hz, *J*_9’,8’_ = 3.2 Hz, 1 H^α^, H-9’^α^), 3.79 (d, *J*_8’,9’_ = 3.5 Hz, 1 H^α^, H-8’^α^), 3.78 (dd, *J*_10’,9’_ = 10.7 Hz, *J*_10’,9’_ = 8.2 Hz, 1 H^β^, H-10’^β^), 3.75 (dd, *J*_7’,6’_ = 8.5 Hz, *J*_7’,8_ = 1.0 Hz, 1 H^β^, H-7’^β^), 3.73 (d, *J*_8’,9’_ = 3.5 Hz, 1 H^β^, H-8’^β^), 3.69 (dd, *J*_9’,10’_ =10.7 Hz, *J*_9’.8’_ = 3.2 Hz, 1 H^β^, H-9’^β^), 1.98 (s, 3 H^α^ + 3 H^β^, -CH_3_^NHAc,β^, -CH_3_^NHAc,α^), 1.86 - 1.96 (m, 1 H^α^ + 1 H^β^, H-6a’^α^, H-6a’^β^), 1.54 - 1.63 (m, 1 H^α^ + 1 H^β^, H-6b’^α^, H-6b’^β^); ^13^C NMR (126 MHz, D_2_O) δ ppm 175.0, 174.7 (C=O^NHAc,β,α^), 166.22 (C-4 C=O^α+β^), 151.9 (C-2 C=O^α+β^), 141.8 (C-6^uracil,α+β^), 102.5 (C-5^uracil,α+β^), 95.3 (C-11’^β^), 90.9 (C-11’^α^), 88.1 (C-1’^α+β^), 87.1, 87.1 (C-4’^α^, C-4’^β^), 73.4, 73.4 (C-2’^α^, C-2’^β^), 71.2, 71.1, 70.8, 70.4 (C-7’^β^, C-8’^β^, C-9’^β^, C-8’^α^), 68.9, 68.9 (C-3’^α^, C-3’^β^), 67.5 (C-9’^α^), 67.1, 67.0 (C-5’^α^, C-5’^β^), 66.3 (C-7’^β^), 53.6 (C-10’^β^), 50.2 (C-10’^α^), 33.7, 33.6 (C-6’^α^, C-6’^β^), 23.3, 22.2 (-CH_3_^NHAc,α^, -CH_3_^NHAc,β^); IR ν: 3362, 1638, 1410, 1264, 1072; LRMS *m/z* (ESI^+^): 470 [(M+Na)^+^, 100%]; (ESI^-^): 482 [(M+Cl)^-^, 100%]; HRMS *m/z* (ESI^+^): calc. for C_17_H_25_N_3_NaO_11_ (M+Na)^+^ = 470.1381, found 470.1367.

## *N*-Octanoyl-*N’*-acetyl tunicamycin (TUN-8,Ac)

A 25 % solution of NaOMe in MeOH (100 µL) was added to anhydrous MeOH (1 mL). An aliquot of the resulting NaOMe solution (100 µL) was then added the mixture of **tunicamycin-8OAc-2Boc** (130 mg, 0.095 mmol) in a MeOH (4 mL) under argon and the resulting orange solution stirred for 4 h. The reaction mixture was carefully quenched with DOWEX 50WX8 H^+^ form resin and the resin was then removed by filtration and the filtrate concentrated *in vacuo*. The resulting yellow solid was dissolved in TFA (2 mL) and stirred at ambient temperature for 2 h. The reaction mixture was then concentrated *in vacuo* and azeotroped with toluene (2 x 1 mL) and MeOH (2 x 1 mL), followed by drying under high vacuum overnight. In a separate flask, octanoic acid (0.204 mmol) and HATU (70.8 mg, 0.186 mmol) were dissolved in dry DMF (1 mL) and cooled to 0 ^o^C. DIPEA (62 µL, 0.354 mmol) was added and the resulting yellow solution stirred at 0 ^o^C for 10 min. A solution of the crude diamine in DMF (1 mL) was added and the resulting yellow solution stirred at ambient temperature for 18 h. The reaction mixture was concentrated *in vacuo* and purified by column chromatography (SiO_2_, 1:3:6 H_2_O:IPA:EtOAc). This was further purified by HPLC and product containing fractions were lyophilized to yield pure **TUN-8,Ac** (3 mg, 0.004 mmol, 4%). ^1^H NMR (400 MHz, CD_3_OD) δ ppm 7.94 (d, 1H, *J* = 8.1 Hz, H6), 5.95 (d, 1H, *J* = 6.0 Hz, H1’), 5.78 (d, 1H, *J* = 8.1 Hz, H5), 4.96 (d, 1H, *J* = 3.4 Hz, H1”), 4.63 (d, 1H, *J* = 8.5 Hz, H11’), 4.26-4.21 (m, 2H, H2’ + H3’), 4.04-3.97 (m, 2H, H5’ + H5”), 3.96-3.86 (m, 4H, H10’ + H2” + H4’ + H6”), 3.73-3.64 (m, 5H, H7’ + H8’ + H9’ + H-3” + H6”), 3.34 (obscured by solvent, 1H, H4”), 2.47-2.17 (m, 2H, Oct-Hα), 2.13-2.05 (m, 1H, H6’), 2.03 (s, 1H, NHAc), 1.63-1.50 (m, 3H, Lipid-Hβ + H6’), 1.40-1.20 (m, 8H, Lipid-Hγ + Hδ + Hε + Hζ + Hη), 0.92-0.89 (m, 3H, Lipid Hθ); ^13^C NMR (151 MHz, CD_3_OD) δ 177.22, 173.57 (N-C=O^fatty acyl^), 166.12 (C-4), 152.62 (C-2), 142.74 (C-6), 103.05 (C-5), 101.21 (C-11’), 99.78 (C-1”), 89.82 (C-1’), 89.60 (C-4’), 75.49 (C-2’), 74.30 (C-5”), 73.06, 72.96 (C-8’, C-9’), 72.51 (C-4”), 72.49 (C-7’), 72.10 (C-3”), 70.90 (C-3’), 68.34 (C-5’), 63.27 (C-6”), 54.95 (C-2”), 54.49 (C-10’), 37.75, 35.92 (CO*C*H_2_-^fatty acyl^), 32.90 (C-6’), 30.43, 30.18, 27.00, 23.68, 23.10 (*C*H_2_-^fatty acyl^), 14.40 (*C*H_3_^fatty acyl^); ); IR ν: 3367, 3192, 1667, 1588, 1368, 1318, 1098, 1019; HRMS (ESI^+^) Calcd for C_31_H_50_O_16_N_4_Na [M+Na]^+^ 757.31140, found 757.31085.

**Solubility study of TUN-8,8**

For the calibration curve, a stock solution of TUN-8,8 (100 mM in DMSO) was dissolved in MeOH to make 100 μl of 1, 0.75, 0.5, 0.25, 0.1 and 0.05 mM solution. 40 μl of each solultion was diluted in 360 μl of H_2_O and injected in RP-HPLC (the retention time was 6.5 min). The area under each peak was plotted.

For the solubility study, a stock solution of TUN-8,8 (1 μl, 100 mM in DMSO) was added to the corresponding matrices (99 μl) to make a 1 mM mixture. The mixture was vortexed, sonicated for 5 min, then warmed for 10 min at 37 °C. The mixture was centrifuged (13,000 rpm, 5 min) and the supernatant was collected. 40 μl of supernatant was diluted in 360 μl of H_2_O and injected in RP-HPLC to determine the concentration. For fetal bovine serum solubility, to avoid crushing the column, precipitate was used to determine the solubility. i.e. the precipitate was dissolved in MeOH (100 μl) and 40 μl of this solution was diluted in 360 μl of H_2_O and injected in RP-HPLC. The fetal bovine serum solubility was calculated by subtracting this concentration.

HPLC condition:

Column: Phenomenex, Synergi 4u Hydro-RP 80Å 100 x 4.60 mm 4micron; Flow rate: 4mL/min; Solvent A: 0.1% FA in H_2_O; Solvent B: 0.1% FA in ACN; UV 254 nm.

Eluent gradient

Min. %B

0.000 30.0[%]

10.000 100.0 [%]


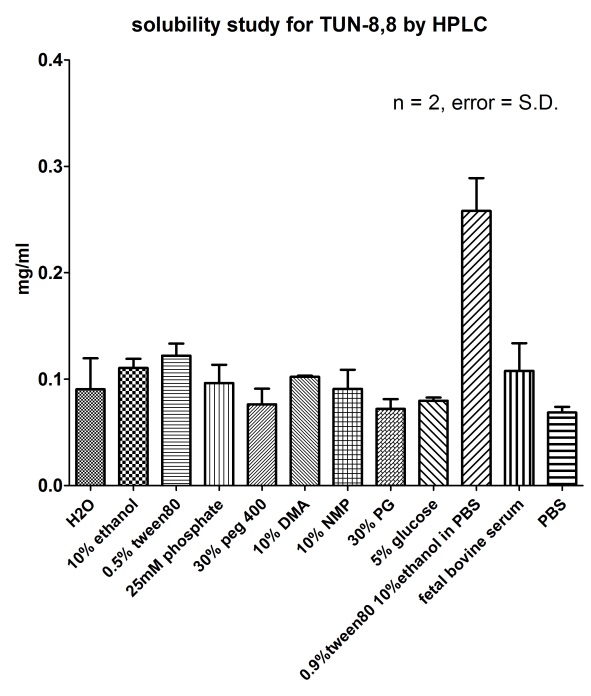


**Purity of TUN-8,8 for mice administration**


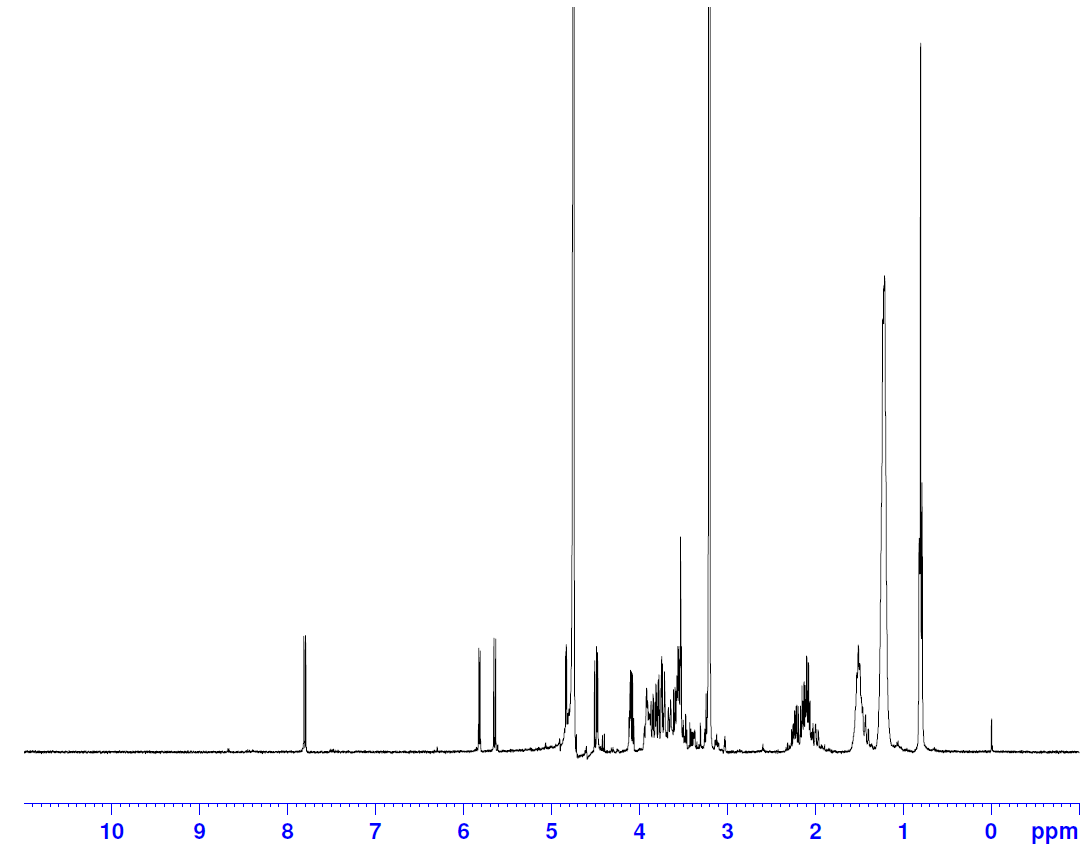


1H-NMR spectrum of the **TUN-8,8** administered to mice. All the peaks were identical with the assigned spectra and no impurity was observed.


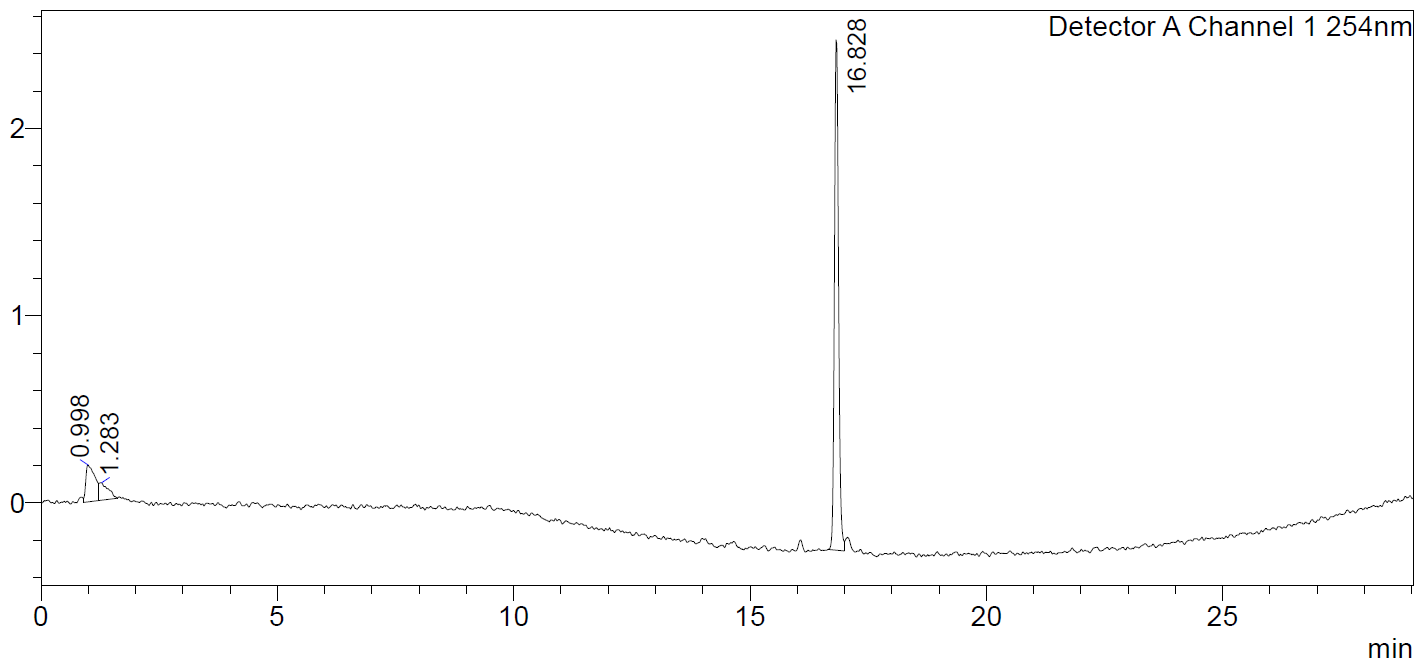


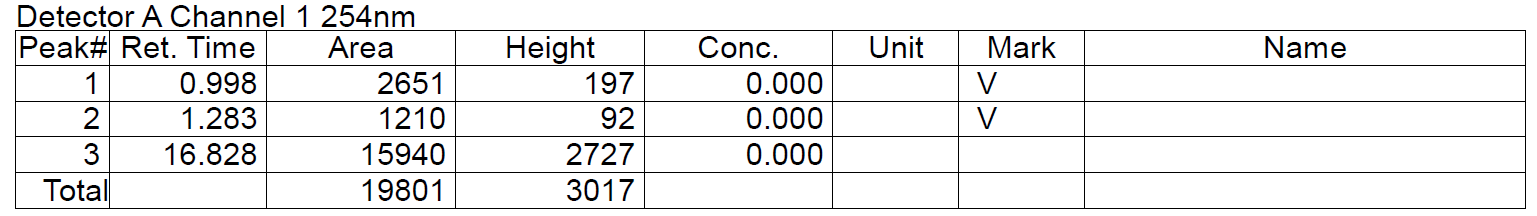


HPLC spectrum of TUN-8,8 for mice administration. The spectrum suggested that the compound was pure and no inpurity was observed. (The initial peak around 1 min is due to the DMSO in the stock solution).

**SI, Scheme 1. The Synthesis of GlcNAc-PP-Und (7).**

## 1,3,4,6-Tetra-*O*-acetyl-*N*-acetyl-d-glucosamine (4)

*N*-Acetyl-d-glucosamine (5.0 g, 22.6 mmol) was suspended in pyridine (50 mL) and Ac_2_O (25 mL) and stirred for 6 h at ambient temperature. The reaction mixture was then concentrated *in vacuo* and azeotroped with toluene (3 x 20 mL). The resulting oil was dissolved in CH_2_Cl_2_ (100 mL), washed with 1 M HCl (50 mL) and brine (50 mL), dried over anhydrous Na_2_SO_4_ and concentrated *in vacuo* to yield product **4** as a white foam (8.47 g, 96%). ^1^H NMR (CDCl_3_, 400 MHz) δ 6.15 (d, 1H, *J* = 3.7 Hz, H1), 5.65 (d, 1H, *J* = 9.1 Hz, NH), 5.25-5.16 (m, 2H, H3 + H4), 4.50-4.44 (m, 1H, H2), 4.23 (dd, 1H, *J* = 12.5, 4.1 Hz, H6), 4.05 (dd, 1H, *J* = 12.5, 2.4 Hz, H6’), 3.98 (ddd, 1H, *J* = 9.7, 4.0, 2.3, H5), 2.18 (s, 3H), 2.07 (s, 3H), 2.04 (s, 3H), 2.03 (s, 3H), 1.92 (s, 3H); ^13^C NMR (125 MHz, CDCl_3_) δ 171.9, 170.9, 170.2, 169.3, 168.8, 90.6, 70.9, 69.9, 67.7, 61.7, 51.2, 23.2, 21.1, 20.9, 20.7; LRMS (ES) Calcd for C_16_H_23_NNaO_10_ [M+Na]^+^ 412.12, found 412.12.

## *N*-acetyl-3,4,6-Tris-*O*-acetyl-1-(dibenzyl phosphate)-α-d-glucosamine (5)

Acetate **4** (3.0 g, 7.71 mmol) was dissolved in dry DMF (50 mL). Hydrazine acetate (1.05 g, 11.4 mmol) was added and the resulting solution stirred at ambient temperature for 2 h. The reaction mixture was diluted with EtOAc (100 mL) and washed with H_2_O (100 mL) and saturated aqueous NaHCO_3_ (100 mL). The combined aqueous washings were back extracted with EtOAc (2 x 50 mL) and the combined organic extracts dried over anhydrous Na_2_SO_4_ and concentrated *in vacuo* to yield the anomeric lactol as a colorless oil (1.25 g, 47 %). The lactol (1.25 g, 3.6 mmol) was then dissolved in anhydrous CH_2_Cl_2_ (30 mL) and added rapidly via syringe to a vigorously stirred suspension of 5-ethylthio-1*H*-tetrazole (2.20 g, 16.9 mmol) and dibenzyl-*N*,*N*’-diisopropylphosphoramidite (3.73 g, 10.8 mmol) in anhydrous CH_2_Cl_2_ (30 mL) under argon at ambient temperature. The reaction mixture became homogeneous within a few min. After 2 h, the mixture was diluted with CH_2_Cl_2_ (40 mL) and washed with saturated sodium bicarbonate (50 mL), water (50mL) and brine (50 mL). The organic solution was dried over anhydrous sodium sulfate and concentrated *in vacuo* to yield the phosphite as a colorless oil. The product was dissolved in THF (60 mL) and cooled to -78 °C. Hydrogen peroxide (30%, 6 mL) was added dropwise via syringe to the vigorously stirred solution. After the addition was complete, the ice bath was removed and the mixture was allowed to warm to ambient temperature over 1.5 h. The reaction mixture was then diluted with ice-cold saturated sodium sulfite (15 mL), followed by EtOAc (30 mL), and stirred for 5 min. The organic layer was concentrated *in vacuo* and the crude redissolved in EtOAc (100 mL). This was washed with saturated NaHCO_3_ (50 mL), H_2_O (50 mL) and brine (50 mL), dried over anhydrous sodium sulfate and concentrated *in vacuo*. The crude product was purified by column chromatography (SiO_2_, 2:98 to 5:95 MeOH:CH_2_Cl_2_) to yield phosphate **5** as a clear oil (1.14 g, 52 %). ^1^H NMR (CDCl_3_, 400 MHz) δ 7.38-7.32 (m, 10H, ArH), 5.84 (d, 1H, *J* = 9.2 Hz, NH), 5.66 (dd, 1H, *J* = 6.1, 3.4 Hz, H1), 5.18-5.00 (m, 6H, 2 x PhCH_2_ + H3 + H4), 4.37 (app. ddt, 1H, *J* = 10.7, 9.3, 3.2 Hz, H2), 4.12 (dd, 1H, *J* = 12.5, 3.9 Hz, H6), 4.00 (ddd, 1H, *J* = 9.6, 3.8, 2.2 Hz, H5), 3.91 (dd, 1H, *J* = 12.5, 2.4 Hz, H6’), 2.01 (s, 3H), 2.00 (s, 6H), 1.70 (s, 3H); ^13^C NMR (125 MHz, CDCl_3_) δ 171.2, 170.6, 170.3, 169.2, 129.0, 128.9, 128.9, 128.8, 128.2, 128.2, 128.1, 96.3, 96.2, 70.1, 70.1, 70.0, 70.0, 69.7, 67.4, 61.3, 51.9, 51.8, 22.8, 20.7, 20.7; LRMS (ES) Calcd for C_28_H_34_NNaO_12_P [M+Na]^+^ 630.2, found 630.2.

## Undecaprenol

Ground bay leaves (100 g, *Laurus nobilis*) were extracted with a refluxing mixture of 9:1 acetone:*n-*hexanes (1500 mL) by soxhlet extraction for 3 days. The resulting green solution was concentrated *in vacuo* and resuspended (not all solids dissolve) in a mixture of *n*-hexanes (150 mL), EtOH (750 mL) and 15% KOH(aq) (100 mL) and refluxed for 1 h. The resulting mixture was cooled to ambient temperature, followed by addition of H_2_O (500 mL) and Et­_2_O (500 mL). The ether extract was separated, dried over anhydrous Na_2_SO_4_ and concentrated *in vacuo*. The resulting orange solid was purified by column chromatography (SiO_2_ (900 g), 100:0 to 95:5 petrol:EtOAc) using authentic undecaprenol (from American Radiolabelled Chemicals) as a TLC standard, to yield undecaprenol as a yellow oil (950 mg). ^1^H NMR (CDCl_3_, 400 MHz) δ 5.46-5.43 (m, 1H, CHCH_2_OH), 5.15-5.08 (m, 10H); 4.09 (dd, 2H, *J* = 7.2, 0.9 Hz, CH_2_OH), 2.09-1.05 (m, 40H), 1.75-1.74 (m, 3H), 1.69-1.67 (m, 21H), 1.61-1.59 (m, 12H); LRMS (ES) Calcd for C_55_H_90_NaO [M+Na]^+^ 789.7, found 789.6.

## Undecaprenyl phosphate bisammonium salt (Und-P)

Undecaprenol (710 mg, 1.0 mmol) was dissolved in anhydrous CH_2_Cl_2_ (10 mL) and added rapidly via syringe to a vigorously stirred suspension of 5-ethylthio-1*H*-tetrazole (570 mg, 4.41 mmol) and bis(2-cyanoethyl)-*N*,*N*’-diisopropylphosphoramidite (0.73 mL, 2.85 mmol) in anhydrous CH_2_Cl_2_ (10 mL) under argon at ambient temperature. The reaction mixture became homogeneous within a few min. After 3 h, the mixture was diluted with CH_2_Cl_2_ (80 mL) and washed with saturated sodium bicarbonate (50 mL), water (50mL) and brine (50 mL). The organic solution was dried over anhydrous sodium sulfate and concentrated *in vacuo* to yield the phosphite as a yellow oil. The product was dissolved in THF (20 mL) and cooled to -78 °C. Hydrogen peroxide (30%, 1.9 mL) was added dropwise via syringe to the vigorously stirred solution. After the addition was complete, the ice bath was removed and the mixture was warmed to ambient temperature over 2 h. The reaction mixture was then diluted with ice-cold saturated sodium sulfite (5 mL) and stirred at 0 ^o^C for 5 min. The reaction mixture was then extracted with EtOAc (80 mL) and the organic layer was washed with saturated NaHCO_3_ (50 mL), water (50 mL) and brine (50 mL), dried over anhydrous sodium sulfate and concentrated *in vacuo* to yield the phosphate as a yellow oil. The crude phosphate was suspended in anhydrous MeOH (17 mL) and a 25 % NaOMe in MeOH solution (0.7 mL) was added. The resulting suspension was stirred at ambient temperature for 16 h. The reaction mixture was diluted with MeOH (30 mL) and CHCl_3_ (30 mL) and carefully neutralized with DOWEX 50WX8 H^+^ form resin. The resin was removed by filtration and the filtrate concentrated *in vacuo*. The resulting yellow oil was purified by column chromatography (SiO_2_, 90:10:0:0.1 to 65:25:5:0.1 CHCl_3_:MeOH:H_2_O:NH_4_OH) to yield **Und-P** as an off-white foam. Aggregation of this compound prevented acquisition of good NMR spectra. LRMS (ES) Calcd for C_55_H_90_O_4_P [M-H]^-^ 845.6, found 845.6.

## *N*-Acetyl-3,4,6-tris-*O*-acetyl-1-[*P*’-(3Z,7Z,11Z,15Z,19Z,23Z,27Z,31E,35E,39E,43-undecamethyl-2,6,10,14,18,22,26,30,34,38,42-tetratetracontaundecaenyl) *P,P*'-dihydrogen diphosphate]-α-d-glucosamine diimidazolium salt (6)

Phosphate **5** (1.00 g, 1.65 mmol) was dissolved in MeOH and the resulting solution degassed with an Ar balloon. A 10 % dispersion of palladium on carbon (1.21 g, 1.14 mmol) was added and a H_2_ balloon bubbled through the resulting suspension. The reaction mixture was then stirred under a H_2_ atmosphere for 18 h at ambient temperature. The reaction mixture was then filtered through celite, which was washed with MeOH. Pyridine (1 mL) was added to the filtrate and the resulting solution concentrated *in vacuo* to yield the phosphate dipyridine salt as a white solid (822 mg, 85%). A portion of this product (59 mg, 0.101 mmol) was dissolved in dry THF (1 mL) and DMF (1 mL) under an Ar atmosphere. CDI (76 mg, 0.468 mmol) was added and the resulting solution stirred at RT for 2 h. Reaction monitoring by LRMS (ESI) indicated complete product formation ([M-H]^-^ = 476.1) at this point. Anhydrous MeOH (14 uL, 0.368 mmol) was added and the reaction mixture stirred for a further 45 min. The reaction mixture was concentrated to remove MeOH and THF. To the resulting DMF solution of CDI activated phosphate was added a solution of **Und-P** (89 mg, 0.101 mmol) in THF (2 mL). 5-ethylthio-1*H*-tetrazole (13 mg, 0.101 mmol) was added to the resulting solution and the reaction mixture stirred for 4 days at ambient temperature. The solution was then concentrated *in vacuo* and purified by column chromatography (SiO_2_, 1:2:4 water:*^i^*PrOH:EtOAc) to yield the product **6** as a white solid (120 mg, 86%). ^1^H NMR (400 MHz, 1:1 CDCl_3_:CD_3_OD) δ 7.80 (s, Imidazole), 7.10 (s, Imidazole), 5.64 (dd, 1H, *J* = 6.9, 3.1 Hz, H1), 5.45-5.41 (m, 1H, =CHCH_2_OP), 5.32 (dd, 1H, *J* = 10.7, 9.5 Hz, H3), 5.15-5.07 (m, 12H, H4 + NH + 10 x sp^2^ C-H), 4.53 (t, 2H, 7.0 Hz, CH_2_OP), 4.36-4.30 (m, 3H, H2 + H5 + H6), 4.41-4.11 (m, 1H, H6’), 2.10-1.95 (m, 52H, 20 x CH_2_, 4 x Ac), 1.73 (s, 3H), 1.68-1.66 (m, 21H), 1.61 (s, 3H), 1.59 (s, 9H); ^32^P NMR (162 MHz, 1:1 CDCl_3_:CD_3_OD) δ -9.7, -12.7; LRMS (ES) Calcd for C_69_H_110_NO_15_P_2_ [M-H]^-^ 1254.7, found 1254.7.

## *N*-Acetyl-1-[*P*’-(3Z,7Z,11Z,15Z,19Z,23Z,27Z,31E,35E,39E,43-undecamethyl-2,6,10,14, 18,22,26,30,34,38,42-tetratetracontaundecaenyl)*P,P*'-dihydrogen diphosphate]-α-d-gluc -osamine diammonium salt (7)

Acetate **6** (120 mg, 0.086 mmol) was suspended in dry MeOH (5 mL) and stirred under an Ar atmosphere. A 25 % solution of NaOMe in MeOH (20 uL, 0.093 mmol) was added and the mixture stirred at ambient temperature for 1 h. The reaction mixture was then quenched with excess DOWEX 50WX8 ammonium form and the resin removed by filtration and concentrated *in vacuo* to yield the product **7** as a white solid (100 mg, quant.). LRMS (ES) Calcd for C_69_H_110_NO_15_P_2_ [M-H]^-^ 1254.7, found 1254.7. NMR acquisition proved difficult due to the challenging solubility parameters of this product.

**SI, Scheme 2. Synthesis of glycosyl donor (9) for lipid II synthesis.**

## 2-Deoxy-2-[[(2,2,2-trichloroethoxy)carbonyl]amino]-3,4,6-triacetyl-1-(2,2,2-trichloroethanimidate)-α-d-glucopyranose (9)

d-Glucosamine (20.00 g, 92.8 mmol) and sodium bicarbonate (15.6 g, 185.6 mmol) were dissolved in water (240 mL) and stirred vigorously for 5 min. 2,2,2-Trichloroethoxycarbonyl chloride (15.3 mL, 111.2 mmol) was added dropwise and the solution stirred at ambient temperature for 2 h, over which time a white precipitate formed. The suspension was filtered, washed with water and coevaporated with toluene (3 x 50 mL). The resulting white powder was dissolved in dry pyridine (200 mL) and acetic anhydride (100 mL) and stirred at ambient temperature for 18 h. The reaction mixture was concentrated *in vacuo* and co-evaporated with toluene (3 x 50 mL). The resulting oily residue was dissolved in CHCl_3_ (200 mL) and washed with 1 M HCl (150 mL). The aqueous phase was back extracted with CHCl_3_ (200 mL) and the combined organic extracts washed with brine (100 mL). The organic phase was then dried over anhydrous sodium sulfate and concentrated *in vacuo* to yield 1,3,4,6-tetra-*O*-acetyl-2-troc-d-glucosamine **8** (31.8 g, 65 %) as a white solid. **8** (31.8 g, 60.8 mmol) was dissolved in dry DMF (300 mL) and hydrazine acetate (6.72 g, 23.0 mmol) was added. The reaction mixture was stirred at ambient temperature for 40 min, diluted with EtOAc (300 mL) and washed with water (300 mL), saturated sodium bicarbonate (200 mL) and water (200 mL). The combined aqueous phases were then back extracted with EtOAc (300 mL) and the combined organic extracts washed with brine (200 mL), dried over anhydrous sodium sulfate and concentrated *in vacuo*. The resulting red oil was dissolved in CH_2_Cl_2_ (300 mL) and trichloroacetonitrile (61 mL, 608 mmol). 1,8-Diazabicycloundec-7-ene (1.81 mL, 12.16 mmol) was added and the reaction mixture stirred at ambient temperature for 90 min and concentrated *in vacuo*. The crude reaction mixture was purified by flash column chromatography (silica, 2:1 hexanes:EtOAc + 0.1% triethylamine) to yield the product **9** as a white foam (31 g, 82%). [α]_D_^25^ = 76.1 (*c* = 1.1 g/100mL, CH_2_Cl_2_); ^1^H NMR (CDCl_3_, 400 MHz) δ 8.80 (s, 1H, acetimidate-NH), 6.43 (d, *J* = 3.6 Hz, 1H, H1), 5.35 (dd, *J* = 10.8, 9.5 Hz, H3), 5.25 (t, *J* = 9.9 Hz, H4), 5.19 (d, *J* = 9.3 Hz, Troc-NH), 4.75 – 4.67 (m, 2H, Troc-CH_2_), 4.29 (m, 2H, H2 + H6), 4.16 – 4.09 (m, 2H, H5 + H6), 2.07 (m, 9H, 3 x OCH_3_); ^13^C NMR (CDCl_3_, 125 MHz) δ 171.0, 170.4, 169.1, 160.3, 154.0, 95.1, 94.4, 90.5, 74.6, 70.2, 67.3, 61.3, 53.8, 20.6, 20.5.

**SI, Scheme 3. Synthesis of alanine ester (10) for lipid II synthesis.**

## l-Alanine-2-(phenylsulfonyl)ethyl ester (10)

Boc-l-alanine (5.08 g, 26.8 mmol), 2-phenylsulfonylethanol (5.00 g, 26.8 mmol), 1-(3-Dimethylaminopropyl)-3-ethylcarbodiimide hydrochloride (5.20 g, 26.8 mmol) and 4-dimethylaminopyridine (0.33 g, 2.68 mmol) were dissolved in dry CH_2_Cl_2_ (150 mL) and stirred at ambient temperature under an argon atmosphere for 18 h. The reaction mixture was then washed with 0.5 M HCl (100 mL) and saturated NaHCO_3_ (100 mL). Each aqueous phase was back extracted with CH­_2_Cl_2_ (50 mL) and the combined organic extracts washed with brine (100 mL). The organic extracts were then dried over anhydrous Na_2_SO_4_ and concentrated *in vacuo*. The resulting orange oil was dissolved in CHCl_3_ (30 mL) and passed through a silica plug, with CHCl_3_ washing until no further product fractions eluted. The solution was concentrated *in vacuo*, redissolved in CH_2_Cl_2_ (40 mL) and cooled to 0 ^o^C. Trifluoroacetic acid (40 mL) was added slowly and the solution warmed to ambient temperature and stirred for 3 hours. The resulting orange solution was then concentrated *in vacuo*, azeotroped with toluene (2 x 10 mL) and purified by column chromatography (SiO_2_, 1:9 MeOH:CH_2_Cl_2_) to yield the product **10** as a colourless oil (quant.). ^1^H NMR (CDCl_3_, 400 MHz) δ 7.89 – 7.86 (m, 2H, *o*-ArH), 7.69 – 7.65 (m, 1H, *p*-ArH), 7.59 – 7.55 (m, 2H, *m*-ArH), 4.53 (t, 2H, *J* = 5.8 Hz, O-CH_2_), 3.91 (app. q, 1H, *J* = 7.2 Hz, Ha); 3.49 – 3.46 (m, 2H, S-CH_2_), 1.30 (d, *J* = 7.3 Hz, 3H, Hβ); ^13^C NMR (CDCl_3_, 125 MHz) δ 175.7, 139.5, 134.2, 129.6, 128.3, 58.1, 55.2, 49.9, 20.2.

**SI, Scheme 4. Synthesis of glycosyl acceptor (14) for lipid II synthesis.**

## Phenylmethyl-2-(acetylamino)-2-deoxy-4,6-*O*-(phenylmethylene)-α-d-glucopyranoside (11)

*N*-Acetyl-d-glucosamine (30 g, 136 mmol) was suspended in benzyl alcohol (300 mL) and 37 % HCl (aq) (7.5 mL) and heated at 95 ^o^C for 4 h. During this time the white suspension became a purple/brown solution. The reaction mixture was concentrated to ~50 mL and precipitated by adding to ice-cold Et_2_O (600 mL) and stirring for 1 h. The off-white precipitate was filtered, was with Et_2_O (3 x 100 mL) and dried under high vacuum overnight. The resulting white solid (38 g) was suspended in benzaldehyde (140 mL) and finely ground anhydrous ZnCl_2_ (38 g) was added. The resulting suspension was stirred at 60 ^o^C until for 1 h, with most solids dissolving after 5 min. The mixture was then poured into a stirring ice-water mixture (400 mL) and the resulting precipitate collected by filtration. The pinkish solid was washed with H_2_O (2 x 200 mL), ice-cold EtOH (100 mL) and Et_2_O (3 x 200 mL). The resulting off-white solid (20 g) was dissolved in boiling pyridine (100 mL) and boiling H_2_O was added until to solution turned cloudy. The solution was then cooled to ambient temperature, followed by ice-bath for 1 h. The resulting precipitate was collected by filtration, washed with H_2_O (2 x 100 mL), azeotroped with toluene (500 mL) and dried by high vacuum overnight to yield the product **11** as a white fluffy solid (11.4 g, 21% over 2 steps). [α]_D_^25^ = +124 (*c* = 1.1 g/100mL, pyridine); ^1^H NMR (DMSO-*d_6_*, 400 MHz) δ 8.00 (d, 1H, *J* = 8.3 Hz, NHAc), 7.46-7.28 (m, 10H, Ar-H), 5.62 (s, 1H, PhCH), 5.19 (d, 1H, *J* = 5.8 Hz, 3-OH), 4.80 (d, 1H, *J* = 3.5 Hz, H1), 4.70 (d, 1H, *J* = 12.6 Hz, PhCHH), 4.49 (d, 1H, *J* = 12.6 Hz, PhCHH), 4.16-4.14 (m, 1H, H6), 3.88-3.82 (m, 1H, H6’), 3.77-3.67 (m, 3H, H2 + H3 + H5), 3.53-3.49 (m, 1H, H4), 1.85 (s, 3H, NHAc).

## *N*-Acetyl-1-*O*-(phenylmethyl)-4,6-*O*-(phenylmethylene)-α-d-muramic acid (12)

Glycol **11** (15.0 g, 37.6 mmol) was suspended in dry dioxane (700 mL) under an argon atmosphere and stirred at 60 ^o^C. A 60% dispersion of NaH in mineral oil (3.0 g, 75 mmol) was added and the mixture stirred for 5 min. (2*S*)-chloropropionic acid (13 mL, 75 mmol) was added and the mixture stirred at 60 ^o^C for a further 10 min. Another portion of a 60% dispersion of NaH in mineral oil (7.5 g g, 188 mmol) was added and the mixture stirred for at 60 ^o^C for 16 h. The reaction mixture was cooled to ambient temperature and H_2_O (400 mL) added slowly with stirring. The dioxane was then removed from the resulting yellow solution by concentration with rotary evaporator. The resulting yellow solution was extracted with CHCl_3_ (200 mL), cooled on ice, acidified to pH 1 with 6M HCl and extracted with CHCl_3_ (3 x 200 mL). The combined organic extracts were washed with H_2_O (200 mL), dried over anhydrous Na_2_SO_4_ and concentrated *in vacuo*. The resulting yellow solid was recrystallized from boiling CHCl_3_ (~250 mL) to yield the product **12** as a white crystalline solid (11.0 g, 54 %). ^1^H NMR (DMSO-*d_6_*, 400 MHz) δ 7.97 (d, 1H, *J* = 8.3 Hz, NHAc), 7.44-7.28 (m, 10H, Ar-H), 5.70 (s, 1H, PhCH), 5.04 (d, 1H, *J* = 3.5 Hz, H1), 4.70 (d, 1H, *J* = 12.4 Hz, PhCHH), 4.49 (d, 1H, *J* = 12.4 Hz, PhCHH), 4.29 (q, 1H, *J* = 6.9 Hz, Ala1-Hα), 4.17-4.13 (m, 1H, H6), 3.83-3.68 (m, 4H, H6’ + H2 + H3 + H5), 1.85 (s, 3H, NHAc), 1.28 (d, *J* = 7.2 Hz, 3H, Ala1-Hβ).

## Phenylmethyl-2-(acetylamino)-2-deoxy-3-*O*-[(1*R*)-1-methyl-2-[[(1*S*)-1-methyl-2-oxo-2-[2-(phenylsulfonyl)ethoxy]ethyl]amino]-2-oxoethyl]-4,6-*O*-[(*R*)-phenylmethylene]-α-d-glucopyranoside (13)

Acid **12** (7.85 g, 16.7 mmol) was suspended in dry CH_2_Cl_2_ (120 mL) and cooled to 0 ^o^C. NMM (1.83 mL, 16.7 mmol) and CDMT (3.51 g, 20.0 mmol) were added and the resulting cloudy suspension stirred at 0 ^o^C for 45 min. A solution of amine **10** (20.0 mmol) and NMM (1.83 mL, 16.7 mmol) in CH_2_Cl_2_ (120 mL) were then added and the resulting solution stirred at ambient temperature overnight. The reaction mixture was then filtered and the filtrate was washed with 1M HCl (100 mL) and brine (100 mL), dried over anhydrous Na_2_SO_4_ and concentrated *in vacuo*. The resulting solid was azeotroped with toluene (2 x 50 mL) and CHCl_3_ (2 x 50 mL) to yield the product **13** as a white solid (11.8 g, 99%). ^1^H NMR (CDCl_3_, 400 MHz) δ 7.91-7.90 (m, 2H, ArH), 7.68-7.64 (m, 1H, ArH), 7.58-7.54 (m, 2H, ArH), 7.50-7.46 (m, 2H, ArH), 7.38-7.27 (m, 8H, ArH), 6.93 (d, *J* = 7.1 Hz, 1H, Ala1-NH), 6.22 (d, *J* = 8.9 Hz, 1H, AcNH), 5.57 (s, 1H, O_2_CH), 4.96 (d, *J* = 3.8 Hz, 1H, H1), 4.71 (d, *J* = 11.8 Hz, 1H, PhCHH), 4.51 – 4.39 (m, 3H, PhCHH + OCH_2_), 4.32-4.23 (m, 2H, H2 + H6), 4.17 (t, *J* = 7.2 Hz, 1H, Ala1-Hα), 4.07 (q, *J* = 6.7 Hz, 1H, OCH), 3.91-3.85 (m, 1H, H5), 3.80-3.64 (m, 3H, H6’ + H3 + H4), 3.48-3.53 (m, 2H, SCH_2_), 1.95 (s, 3H, Ac), 1.38 (d, *J* = 6.8 Hz, 3H, MurNAc-CH_3_), 1.30 (d, *J* = 7.2 Hz, 3H, Ala1-Hβ); ^13^C NMR (CDCl_3_, 125 MHz) δ 173.2, 170.9, 170.65, 137.20, 136.8, 134.2, 129.6, 128.8, 128.4, 128.3, 128.2, 126.1, 101.60, 97.6, 81.7, 78.5, 78.2, 70.3, 69.0, 63.3, 58.2, 55.0, 53.2, 48.10, 23.5, 19.5, 17.3.

## Phenylmethyl-2-(acetylamino)-2-deoxy-3-*O*-[(1*R*)-1-methyl-2-[[(1*S*)-1-methyl-2-oxo-2-[2-(phenylsulfonyl)ethoxy]ethyl]amino]-2-oxoethyl]-6-*O*-(phenylmethyl)-α-d-glucopyranoside (14)

Benzylidene **13** (6.00 g, 8.44 mmol) was dissolved in dry CH_2_Cl_2_ (75 mL) and cooled to 0 ^o^C. Triethylsilane (6.72 mL, 42.2 mmol) was added and the solution stirred for 5 min. TFA (3.23 mL, 42.2 mmol) was then added over 5 min and the reaction mixture stirred for 6 h at 0 ^o^C. Another portion of TFA (1.94 mL, 25.3 mmol) was added at once and the reaction mixture stirred for a further 18 h at 0 ^o^C. The reaction mixture was then diluted with CH_2_Cl_2_ (100 mL) and washed with saturated aqueous NaHCO_3_ (100 mL). The aqueous phase was back extracted with CH_2_Cl_2_ (2 x 100 mL) and the combined organic extracts washed with brine (100 mL), dried over anhydrous Na_2_SO_4_ and concentrated *in vacuo*. The resulting crude product was purified by column chromatography (SiO_2_, 8:2 to 10:0 EtOAc:petrol) to yield glycosyl acceptor **14** was a white foam (3.65 g, 61%). ^1^H NMR (CDCl_3_, 400 MHz) δ 7.94-7.85 (m, 2H, ArH), 7.69-7.63 (m, 1H, ArH), 7.57 (t, *J* = 7.7 Hz, 2H, ArH), 7.41-7.22 (m, 9H, ArH), 6.92 (d, *J* = 7.2 Hz, 1H, Ala1-NH), 6.10 (d, *J* = 9.0 Hz, 1H, MurNAc-NH), 4.92 (d, *J* = 3.6 Hz, 1H, H1), 4.71 (d, *J* = 11.8 Hz, 1H, OC*H*H), 4.65-4.54 (m, 2H, OC*H*H + OCH*H*), 4.49-4.34 (m, 3H, OCH*H* + OCH_2_), 4.29-4.17 (m, 2H, H2 + Ala1-Hα), 4.13 (t, *J* = 6.7 Hz, 1H, MurNAc-OCH), 3.80 (dd, *J* = 9.5, 4.6 Hz, 1H, H5), 3.74 (dd, *J* = 10.3, 4.5 Hz, 1H, H6), 3.72-3.65 (m, 2H, H3 + H4), 3.53 (dd, *J* = 10.5, 8.7 Hz, 1H, H6), 3.39 (ddd, *J* = 9.2, 6.6, 5.5 Hz, 2H, S-CH_2_), 3.01 (s, 1H, OH), 1.90 (s, 3H, NHAc), 1.40 (d, *J* = 6.7 Hz, 3H, MurNAc-CH_3_), 1.30 (d, *J* = 7.2 Hz, 3H, Ala1-Hβ); ^13^C NMR (CDCl_3_, 125 MHz) δ 173.1, 171.91, 170.4, 139.2, 137.9, 137.1, 134.2, 129.6, 128.7, 128.6, 128.3, 128.3, 128.2, 127.9, 127.8, 97.2, 80.6, 77.9, 73.8, 71.7, 70.5, 70.3, 69.9, 58.1, 55.0, 52.6, 48.0, 23.5, 19.1, 17.2.

**SI, Scheme 5. Synthesis of tetrapeptide (17) for lipid II synthesis.**

## Boc-d-Ala-d-Ala-OMe (15)

H-d-Ala-OMe.HCl (5.00 g, 35.8 mmol), Boc-d-Ala-OH (6.78 g, 35.8 mmol) and HATU (13.60 g, 35.8 mmol) were dissolved in dry DMF (175 mL) and cooled to 0 ^o^C. DIPEA (6.25 mL, 107.4 mmol) was added and the reaction stirred at ambient temperature for 18 h. The solution was then concentrated *in vacuo*, re-dissolved in EtOAc (200 mL) and washed with 0.5 M HCl (100 mL), saturated sodium bicarbonate (100 mL) and brine (100 mL). The organic phase was then dried over anhydrous sodium sulfate and concentrated *in vacuo*. The crude material was dissolved in CHCl_3_ (100 mL), filtered through celite and concentrated *in vacuo* to yield Boc-dipeptide **15** as a white foam (7.75 g, 79%). ^1^H NMR (CDCl_3_, 400 MHz) δ 6.67 (m, 1H, d-Ala5NH), 5.03 (m, 1H, d-Ala4NH), 4.56 (app. pentet, *J* = 7.2 Hz, 1H, d-Ala5Hα), 4.17 (m, 1H, d-Ala4Hα), 3.74 (s, 3H, d-Ala5-OMe), 1.44 (m, 9H, Boc), 1.39 (d, *J* = 7.1 Hz, 3H, d-Ala5Hβ), 1.35 (d, *J* = 7.1 Hz, 3H, d-Ala4Hβ). ^13^C NMR (CDCl_3_, 125 MHz) δ 173.3, 172.3, 52.6, 48.1, 28.4, 18.5, 18.4.

## Boc-Lys-d-Ala-d-Ala-OMe (16)

Boc-dipeptide **15** (3.00 g, 10.9 mmol) was dissolved in CH_2_Cl_2_ (30 mL) and cooled to 0 ^o^C. TFA (30 mL) was added and the resulting solution stirred at ambient temperature for 3 h. The reaction mixture was then concentrated *in vacuo*, azeotroped with toluene and dried under high vacuum for 1 h. During this time, in a separate flask, Boc-Lys(TFA)-OH (3.73 g, 10.9 mmol) and HATU (4.14 g, 10.9 mmol) were dissolved in dry DMF (50 mL) and cooled to 0 ^o^C. DIPEA (5.70 mL, 32.7 mmol) was added and the resulting yellow solution stirred at 0 ^o^C for 15 min. The deprotected dipeptide (10.9 mmol) was dissolved in DMF (10 mL) and added to the activated acid solution. The resulting reaction mixture was stirred at ambient temperature overnight and concentrated *in vacuo*. The resulting oil was redissolved in EtOAc (100 mL), washed with 1M HCl (100 mL), saturated aqueous NaHCO_3_ (100 mL) and brine (100 mL), dried over anhydrous Na_2_SO_4_ and concentrated *in vacuo* to yield the product **16** as a white foam (5.4 g, 99%). ^1^H NMR (DMSO-*d_6_*, 400 MHz) δ 9.34 (t, 1H, *J* = 5.5 Hz, Lys3-NHTFA), 8.20 (d, 1H, *J* = 7.1 Hz, d-Ala4-NH), 8.01 (d, 1H, *J* = 7.9 Hz, d-Ala5-NH), 6.93 (d, 1H, *J* = 7.5 Hz, Lys3-NH), 4.35-4.23 (m, 2H, Lys3-Hα + d-Ala4-Hα), 3.89-3.84 (m, 1H, d-Ala5-Hα), 3.60 (s, 3H, OMe), 3.15 (app. q, 2H, *J* = 6.5 Hz, Lys3-Hε), 1.60-1.40 (m, 4H, Lys3-Hβ + Lys3-Hδ), 1.36 (s, 9H, *t*Bu), 1.31-1.17 (m, 8H, Lys3-Hγ + d-Ala4-Hβ + d-Ala5-Hβ); ^13^C NMR (CDCl_3_, 125 MHz) δ 172.8, 172.0, 171.7, 78.2 51.9, 47.6, 47.5, 28.1, 27.9, 22.7, 18.2, 16.8; LRMS (ESI) Calcd for C_20_H_33_F_3_N_4_NaO_7_ [M+Na]^+^ 521.2, found 521.2.

## H-γ-d-Glu(α-OMe)-Lys(TFA)-d-Ala-d-Ala-OMe trifluoroacetate salt (17)

Boc-Tripeptide **16** (5.4 g, 10.8 mmol) was dissolved in CH_2_Cl_2_ (30 mL) and cooled to 0 ^o^C. TFA (30 mL) was added and the resulting solution stirred at ambient temperature for 2 h. The reaction mixture was then concentrated *in vacuo*, azeotroped with toluene and dried under high vacuum for 1 h. During this time, in a separate flask, Boc-γ-d-Glu(α-OMe)-OH (2.92 g, 10.8 mmol) and HATU (4.10 g, 10.8 mmol) were dissolved in dry DMF (50 mL) and cooled to 0 ^o^C. DIPEA (5.70 mL, 32.4 mmol) was added and the resulting yellow solution stirred at 0 ^o^C for 15 min. The deprotected tripeptide (10.8 mmol) was dissolved in DMF (15 mL) and added to the activated acid solution. The resulting reaction mixture was stirred at ambient temperature overnight and concentrated *in vacuo*. The resulting oil was redissolved in EtOAc (100 mL) and DMF (5 mL), washed with 1M HCl (100 mL), saturated aqueous NaHCO_3_ (100 mL) and brine (100 mL), dried over anhydrous Na_2_SO_4_ and concentrated *in vacuo* to yield the Boc-tetrapeptide as a white powder (5.85 g, 84%). A portion of the crude Boc-tetrapeptide (2.00 g, 3.11 mmol) was suspended in CH_2_Cl_2_ (10 mL). TFA (10 mL) was added, at which point all solids dissolved. The reaction mixture was stirred for 2 h at ambient temperature, concentrated *in vacuo* and azeotroped with MeOH (2 x 8 mL), CH_2_Cl_2_ (2 x 10 mL) to yield the product **17** was an off-white foam (1.91 g, 96%). ^1^H NMR (DMSO-*d_6_*, 400 MHz) δ 9.42 (t, 1H, *J* = 5.5 Hz, Lys3-NHTFA), 8.41 (br. s, 3H, d-Glu2-H_3_N^+^), 8.27 (d, 1H, *J* = 7.0 Hz, d-Ala4-NH), 8.22 (d, 1H, *J* = 7.9 Hz, d-Ala5-NH), 8.13 (d, 1H, *J* = 7.8 Hz, Lys3-NH), 4.34-4.20 (m, 4H, d-Glu2-Hα + Lys3-Hα + d-Ala4-Hα + d-Ala5-Hα), 3.73 (s, 3H, d-Glu2-OMe), 3.60 (s, 3H, d-Ala5-OMe), 3.15 (app. q, 2H, *J* = 6.4 Hz, Lys3-Hε), 2.38-2.24 (m, 2H, d-Glu-Hγ), 2.04-1.92 (m, 2H d-Glu-Hβ), 1.64-1.43 (m, 4H, Lys3-Hβ + Lys3-Hδ), 1.30-1.18 (m, 8H, Lys3-Hγ + Ala4-Hβ + Ala5-Hβ); ^13^C NMR (DMSO-*d_6_*, 400 MHz) δ 172.9, 172.1, 171.2, 170.7, 169.8, 52.8, 52.5, 51.9, 51.6, 47.6, 31.7, 30.2, 28.0, 25.9, 22.5, 18.2, 16.8; LRMS (ESI) Calcd for C_20_H_33_F_3_N_4_NaO_7_ [M+Na]^+^ 521.2, found 521.2.

**SI, Scheme 6. Synthesis of lipid II.**

## Phenylmethyl-2-(acetylamino)-2-deoxy-3-*O*-[(1*R*)-1-methyl-2-[[(1*S*)-1-methyl-2-oxo-2-[2-(phenylsulfonyl)ethoxy]ethyl]amino]-2-oxoethyl]-6-*O*-(phenylmethyl)-4-*O*-[3,4,6-tri-*O*-acetyl-2-deoxy-2-[[(2,2,2-trichloroethoxy)carbonyl]amino]-β-d-glucopyranosyl]-α-d-glucopyranoside (18)

4 Å molecular sieves (25 g) in a round-bottomed flask were heated under vacuum with a heat gun for approximately 5 min and left to cool at ambient temperature. The flask was depressurized with argon and directly used in the reaction. A solution of glycol **14** (3.2g, 4.49 mmol) in alcohol-free CH_2_Cl_2_ (50 mL) was added to this flask under argon and the suspension gently stirred. TMSOTf (0.81 mL, 4.49 mmol) was added, followed by a solution of acetimidate **9** (8.42 g, 13.47 mmol) in dry CH_2_Cl_2_ (50 mL). The resulting suspension was stirred at ambient temperature overnight. Another portion of acetimidate **9** (5.61 g, 8.98 mmol) and TMSOTf (0.4 mL, 2.25 mmol) were added and the reaction stirred for a further 24 h. The reaction mixture was decanted and the solution diluted with CH_2_Cl_2_ (100 mL). The organic solution was washed with saturated sodium bicarbonate (100 mL) and brine (100 mL) and dried over anhydrous sodium sulfate. The solution was concentrated *in vacuo* and purified by column chromatography (SiO_2_, gradient: 1:1 EtOAc:petrol to EtOAc) to yield the product **18** as a white foam (3.2 g, 61%). ^1^H NMR (CDCl_3_, 400 MHz) δ 7.91-7.90 (m, 2H, ArH), 7.67-7.63 (m, 1H, ArH), 7.58-7.43 (m, 6H, ArH), 7.35-7.25 (m, 6H, ArH), 6.85 (d, *J* = 7.5 Hz, 1H, Ala1NH), 6.53 (d, *J* = 7.5 Hz, 1H, MurNAc-NH), 5.08 (d, *J* = 3.6 Hz, 1H, MurNAc-H1), 4.97 (t, *J* = 9.6 Hz, 1H, GlcNAc-H4), 4.87 (d, *J* = 12.0 Hz, 1H, MurNAc-1-CHHPh), 4.79-4.73 (m, 2H, GlcNAc-H3 + Troc-CHH), 4.62-4.56 (m, 2H, Troc-CHH + MurNAc-6-CHHPh), 4.47-4.32 (m, 4H, OCHH + MurNAc-6-CHHPh + OCHH + MurNAc-1-CHHPh), 4.25 – 4.07 (m, 5H, MurNAc-H2 + MurNAc-CHO + GlcNAc-H1 + GlcNAc-H6 + Ala1Hα), 3.98 (dd, *J* = 12.3, 1.9 Hz, 1H, GlcNAc-H6), 3.91 (t, *J* = 9.5 Hz, 1H, MurNAc-H3), 3.69-3.51 (m, 3H, MurNAc-H6 + MurNAc-H4 + MurNAc-H5), 3.44-3.38 (m, 4H, CH_2_S + GlcNAc-H2 + GlcNAc-H5), 2.05 – 1.98 (m, 9H, 3 x Ac), 1.90 (s, 3H, Ac), 1.34 (d, *J* = 6.7 Hz, 3H, Ala1Hβ), 1.23 (dd, *J* = 9.7, 7.2 Hz, 3H, MurNAc-CH_3_). ^13^C NMR (CDCl_3_, 125 MHz) δ 173.4, 171.9, 170.7, 170.5, 170.4, 169.5, 154.2, 139.3, 137.4, 137.2, 134.1, 129.5, 129.2, 128.6, 128.2, 128.2, 100.1, 97.2, 95.7, 77.7, 75.7, 74.6, 73.8, 72.2, 71.3, 70.5, 70.4, 68.4, 67.2, 61.5, 58.2, 56.3, 55.0, 53.7, 47.8, 23.3, 20.7, 18.4, 17.6; LRMS (ESI) Calcd for C_51_H_62_Cl_3_N_3_NaO_20_S [M+Na]^+^ 1196.2, found 1196.2.

## Phenylmethyl 2-(acetylamino)-2-deoxy-3-*O*-[(1*R*)-1-methyl-2-[[(1*S*)-1-methyl-2-oxo-2-[2-(phenylsulfonyl)ethoxy]ethyl]amino]-2-oxoethyl]-4-*O*-[3,4,6-tri-*O*-acetyl-2-(acetylamino)-2-deoxy-β-D-glucopyranosyl]-6-*O*-acetyl-α-d-glucopyranoside (19)

The Troc-disaccharide **18** (3.0 g, 2.55 mmol) was dissolved in Ac_2_O (12 mL) and AcOH (6 mL) and to this solution was added a solution of anhydrous ZnCl_2_ (3.48 g, 25.5 mmol) in Ac_2_O (5.5 mL) and AcOH (2.5 mL). The reaction mixture was stirred for 24 h at ambient temperature, at which point zinc dust (6.67 g, 102.0 mmol) and a mixture of THF (20 mL), Ac_2_O (13 mL) and AcOH (7 mL) were added. The reaction was stirred for a further 24 h at ambient temperature and filtered through celite, washed with EtOAc (300 mL), and concentrated *in vacuo*. The resulting residue was co-evaporated with toluene (2 x 50 mL) and re-dissolved in EtOAc (200 mL). The organic layer was washed with saturated sodium bicarbonate (2 x 10 mL), which was then back-extracted with EtOAc (100 mL). The combined organics were washed with water (100 mL) and brine (100 mL) and dried over anhydrous Na_2_SO_4_ and concentrated *in vacuo*. The crude product was purified by column chromotography (SiO_2_, EtOAc) to yield the product **19** as a white foam (1.8 g, 71%). ^1^H NMR (CDCl_3_, 400 MHz) δ 7.90-7.88 (m, 2H, *ortho*-ArH), 7.69-7.65 (m, 1H, *para*-ArH), 7.59-7.55 (m, 2H, *meta*-ArH), 7.34-7.25 (m, 5H, ArH), 7.14 (d, *J* = 7.6 Hz, 1H, MurNAc-NH), 6.81 (d, *J* = 6.9 Hz, 1H, Ala1NH), 6.04 (d, *J* = 9.5 Hz, 1H, GlcNAc-NH), 5.12-5.10 (m, 2H, MurNAc-H1 + GlcNAc-H3), 4.63 (d, *J* = 12.1 Hz, 1H, MurNAc-1-CHHPh), 4.49 (d, *J* = 12.1 Hz, 1H, MurNAc-1-CHHPh), 4.40-4.22 (m, 6H, OCH_2_ + MurNAc-CHO + GlcNAc-H1 + GlcNAc-H6 + MurNAc-H6), 4.16-4.08 (m, 2H, MurNAc-H61H + GlcNAc-H6), 4.06-3.98 (m, 3H, GlcNAc-H2 + MurNAc-H2 + MurNAc-H3), 3.78 (d, *J* = 5.2 Hz, MurNAc-H5), 3.62-3.50 (m, 2H, GlcNAc-H5 + MurNAc-H4), 3.40-3.30 (m, 2H, CH_2_S), 2.14 (s, 3H), 2.02 (s, 3H), 2.01 (s, 3H), 2.00 (s, 3H), 1.95 (m, 3H), 1.92 (s, 3H), 1.37 (d, *J* = 6.7 Hz, 3H, MurNAc-CH_3_), 1.29 (d, *J* = 7.2 Hz, 3H, Ala1Hβ); ^13^C NMR (CDCl_3_, 400 MHz) δ 173.8, 172.0, 171.3, 171.0, 170.9, 170.7, 170.7, 169.4, 139.2, 137.4, 134.2, 129.5, 128.6, 128.2, 128.1, 128.0, 100.4, 97.0, 76.1, 75.7, 72.6, 71.9, 70.4, 69.6, 68.2, 62.4, 61.7, 60.5, 58.1, 55.0, 54.7, 53.7, 47.9, 23.3, 23.3, 21.1, 20.7, 20.7, 20.7, 18.4, 17.4. LRMS (ES) Calcd for C_45_H_59_N_3_NaO_20_S [M+Na]^+^ 1016.3, found 1016.3.

## *N*-[*N*-Acetyl-6-*O*-acetyl-1-*O*-[bis(phenylmethoxy)phosphinyl]-4-*O*-[3,4,6-tri-*O*-acetyl-2-(acetylamino)-2-deoxy-β-d-glucopyranosyl]-α-muramoyl]-l-alanine-2-(phenylsulfonyl)ethyl ester (20)

Benzyl ether **19** (1.0 g, 1.0 mmol) was dissolved in THF and MeOH (4:1, 40 mL) and degassed with an Ar balloon. A suspension of 10% palladium on charcoal (1.8 g, 1.7 mmol) was added to this solution and a H_2_ balloon bubbled through the resulting mixture. The reaction mixture was then stirred under hydrogen pressure (10 psi) for 6 h and filtered through a thin layer of celite. The celite was washed with MeOH (2 x 50 mL), the filtrate concentrated *in vacuo* and the resulting oil precipitated from ether and hexanes. The precipitate was filtered and dried to yield the lactol as a white solid (850 mg, 94%). The lactol was then dissolved in anhydrous CH_2_Cl_2_ (10 mL) and added rapidly via syringe to a vigorously stirred suspension of 5-ethylthio-1*H*-tetrazole (575 mg, 4.41 mmol) and dibenzyl-*N*,*N*’-diisopropylphosphoramidite (0.96 mL, 2.85 mmol) in anhydrous CH_2_Cl_2_ (10 mL) under argon at ambient temperature. The reaction mixture became homogeneous within a few min. After 2 h, the mixture was diluted with CH_2_Cl_2_ (80 mL) and washed with saturated sodium bicarbonate (50 mL), water (50mL) and brine (50 mL). The organic solution was dried over anhydrous sodium sulfate and concentrated *in vacuo* to yield a colourless oil, which was precipitated from ether and hexanes (1:1) to yield the phosphite as a white solid. The product was dissolved in THF (20 mL) and cooled to -78 °C. Hydrogen peroxide (30%, 1.9 mL) was added dropwise via syringe to the vigorously stirred solution. After the addition was complete, the ice bath was removed and the mixture was allowed to warm to ambient temperature over 2 h. The reaction mixture was then diluted with ice-cold saturated sodium sulfite (5 mL), followed by EtOAc (50 mL), and stirred for 5 min. The organic layer was washed with saturated NaHCO_3_ (20 mL) and brine (20 mL), dried over anhydrous sodium sulfate and concentrated *in vacuo* to yield phosphate **20** as a white solid (1.02 g, 93%). ^1^H NMR (DMSO-*d*_6_, 400 MHz) δ 8.70 (d, *J* = 4.6 Hz, 1H, NHAc), 8.43 (d, *J* = 6.9 Hz, 1H, NHAc), 8.09 (d, *J* = 9.0 Hz, 1H, NHAc), 7.87-7.85 (m, 2H, *ortho*-ArH), 7.76-7.72 (m, 1H, *para*-ArH), 7.65-7.62 (m, 2H, *meta*-ArH), 7.39-7.30 (m, 10H, 2 x Bn-ArH), 5.81 (dd, *J* = 6.3, 3.1 Hz, 1H, MurNAc-H1), 5.24 (t, *J* = 9.9 Hz, 1H, GlcNAc-H3), 5.08-4.96 (m, 4H, 2 x C*H_2_*Ph), 4.91 (t, *J* = 9.8 Hz, 1H, GlcNAc-H4), 4.73 (d, *J* = 8.3 Hz, 1H, GlcNAc-H1), 4.60 (d, *J* = 6.7 Hz, 1H, MurNAc-C*H*O), 4.33-4.18 (m, 3H, MurNAc-H6 + GlcNAc-H6 + OC*H*H), 4.08-3.95 (m, 4H, MurNAc-H6 + GlcNAc-H6 + OC*H*H + Ala1Hα), 3.87-3.73 (m, 4H, GlcNAc-H2 + GlcNAc-H5 + MurNAc-H3 + MurNAc-H5), 3.64-3.55 (m, 3H, MurNAc-H2 + SC*H_2_*), 3.42 (dd, *J* = 10.9, 8.7 Hz, 1H, MurNAc-H4), 1.97 (s, 3H), 1.96 (s, 3H), 1.95 (s, 3H), 1.92 (s, 3H), 1.75 (s, 3H), 1.69 (s, 3H), 1.29 (d, *J* = 6.7 Hz, 3H, MurNAc-C*H_3_*), 1.11 (d, *J* = 7.3 Hz, 3H, AlaHβ); ^13^C NMR (DMSO-*d*_6_, 125 MHz) δ 174.5, 171.3, 169.9, 169.8, 169.5, 169.3, 139.2, 135.7, 133.9, 129.3, 128.4, 128.3, 128.3, 128.3, 127.9, 127.8, 127.7, 127.6, 127.6, 99.6, 75.9, 75.7, 73.8, 72.3, 70.7, 70.4, 68.6, 68.4, 68.4, 68.3, 66.3, 61.6, 57.9, 53.6, 47.3, 40.0, 39.9, 39.8, 39.7, 39.6, 39.6, 39.5, 39.4, 39.3, 39.1, 39.0, 22.6, 22.3, 20.5, 20.3, 20.2, 18.9, 16.5; RMS (ES) Calcd for C_52_H_66_N_3_NaO_23_PS [M+Na]^+^ 1186.3, found 1186.3.

## *N*-[*N*-Acetyl-6-*O*-acetyl-1-*O*-[bis(phenylmethoxy)phosphinyl]-4-*O*-[3,4,6-tri-*O*-acetyl-2-(acetylamino)-2-deoxy-β-d-glucopyranosyl]-α-muramoyl]-l-alanyl-l-γ-glutamyl-*N*6-(2,2,2-trifluoroacetyl)-l-lysyl-d-alanyl-2,5-dimethyl ester (21)

Disaccharidyl ester **20** (350 mg, 0.3 mmol) was dissolved in dry CH_2_Cl_2_ (3 mL) and stirred at ambient temperature under argon. A solution of Diazabicycloundec-7-ene (45 μL, 0.3 mmol) was added and the resulting solution stirred for 1 h. The reaction mixture was diluted with CH_2_Cl_2_ (15 mL), washed with 1 M HCl (5 mL) and brine (5 mL), dried over anhydrous Na_2_SO_4_ and concentrated *in vacuo*. The oil was precipitated with Et_2_O dried under high vacuum for 2 h to yield the acid as a white solid. The acid was dissolved in dry DMF (5 mL) and cooled to 0 ^o^C with an ice-bath. HATU (114 mg, 0.3 mmol), followed by DIPEA (157 μL, 0.9 mmol) were added and the resulting yellow solution stirred for 15 min. Tetrapeptide **13** (191 mg, 0.3 mmol) was added and the resulting solution stirred at ambient temperature for 24 h. The reaction mixture was then concentrated *in vacuo* and re-dissolved in CHCl_3_ and IPA (9:1, 10 mL) and washed with 1 M HCl (5 mL) and saturated sodium bicarbonate (5 mL). Both aqueous washes were back-extracted with CHCl_3_ (5 mL) and the combined organic extracts washed with brine (2 x 5 mL), dried over anhydrous sodium sulfate and concentrated *in vacuo*. The crude product with precipitated from Et_2_O to yield the pentapeptidyl disaccharide **21** as an off-white solid (410 mg, 90%), which was used directly in the next step without further purification.

## Lipid II diammonium salt (22)

Dibenzyl phosphate **21** (50 mg, 33 μmol) was dissolved in anhydrous MeOH (6 mL) and the flask flushed with an argon baloon. Pd/C (10 % w/w, 106 mg, 99 μmol) was added and the resulting suspension stirred under a H_2_ atmosphere for 3 h. The suspension was then filtered through celite, which was washed with MeOH (2 x 3 mL). Pyridine (1 mL) was added to the filtrate, which was then concentrated *in vacuo* and dried by high vacuum for 1 h to yield the sugar phosphate salt as a white solid. This salt was dissolved in dry DMF (1 mL) and dry THF (1 mL) and carbonyl diimidazole (26.8 mg, 165 μmol) was added. The resulting clear solution was stirred at ambient temperature for 2 h, at which point analysis by ESI showed complete product formation ([M-H]^-^ = 1388.4). Excess carbonyl diimidazole was destroyed by the addition of dry MeOH (5.34 μL, 132 μmol) and stirring continued for 45 min. The reaction mixture was then concentrated *in vacuo* and dried under high vac for 1 h. To resulting activated phosphate was added a solution of UPBA (29 mg, 33 μmol) in THF (2 mL) and 5-ethylthio-1*H*-tetrazole (4.3 mg, 33 μmol). The resulting solution was stirred for 96 h under argon at ambient temperature and concentrated *in vacuo*. To this crude mixture was added 1,4-dioxane (1 mL) and a solution of sodium hydroxide (40 mg, 1 mmol) in water (1 mL). The resulting mixture was stirred at 37 ^o^C for 2 h and filtered through an aqueous filter disc, which was washed with 1:1 H­_2_O/1,4-dioxane (2 mL). Lipid II was then purified by HPLC: column = Phenomenex Luna C_18_(2) 100 Å prep-scale column; flow-rate = 20 mL/min, UV = 220 nm, method: solvent A = 50 mM NH_4_HCO_3_(aq), solvent B = MeOH, gradient = 2 to 98 % B over 30 min, 98 % B for 10 min, 98 to 2 % B over 1 min and 2 % B for 4 min. Lipid II eluted between 33.7 – 34.4 min. Product containing fractions were concentrated by rotary evaporator and diluted with H­_2_O, frozen and lyophilized to yield Gram-positive lipid II **22** as a fluffy white powder (17.7 mg, 29%). HRMS (ESI) Calcd for C_94_H_154_N_8_O_26_P_2_ [M-2H]^-^ 936.52302, found 936.52667.

TUN spectra

Octa-*O*-acetyl-tunicamycin
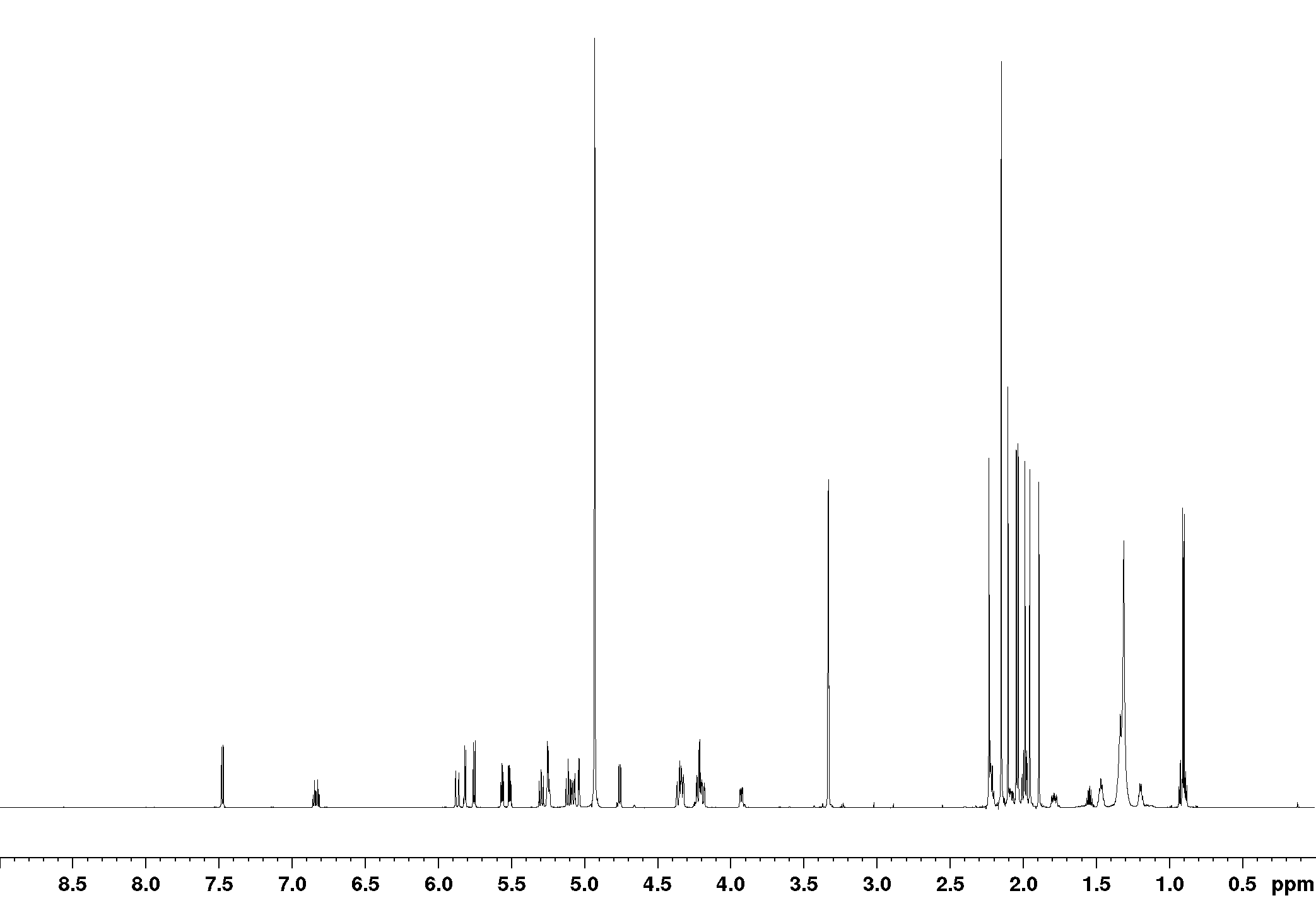


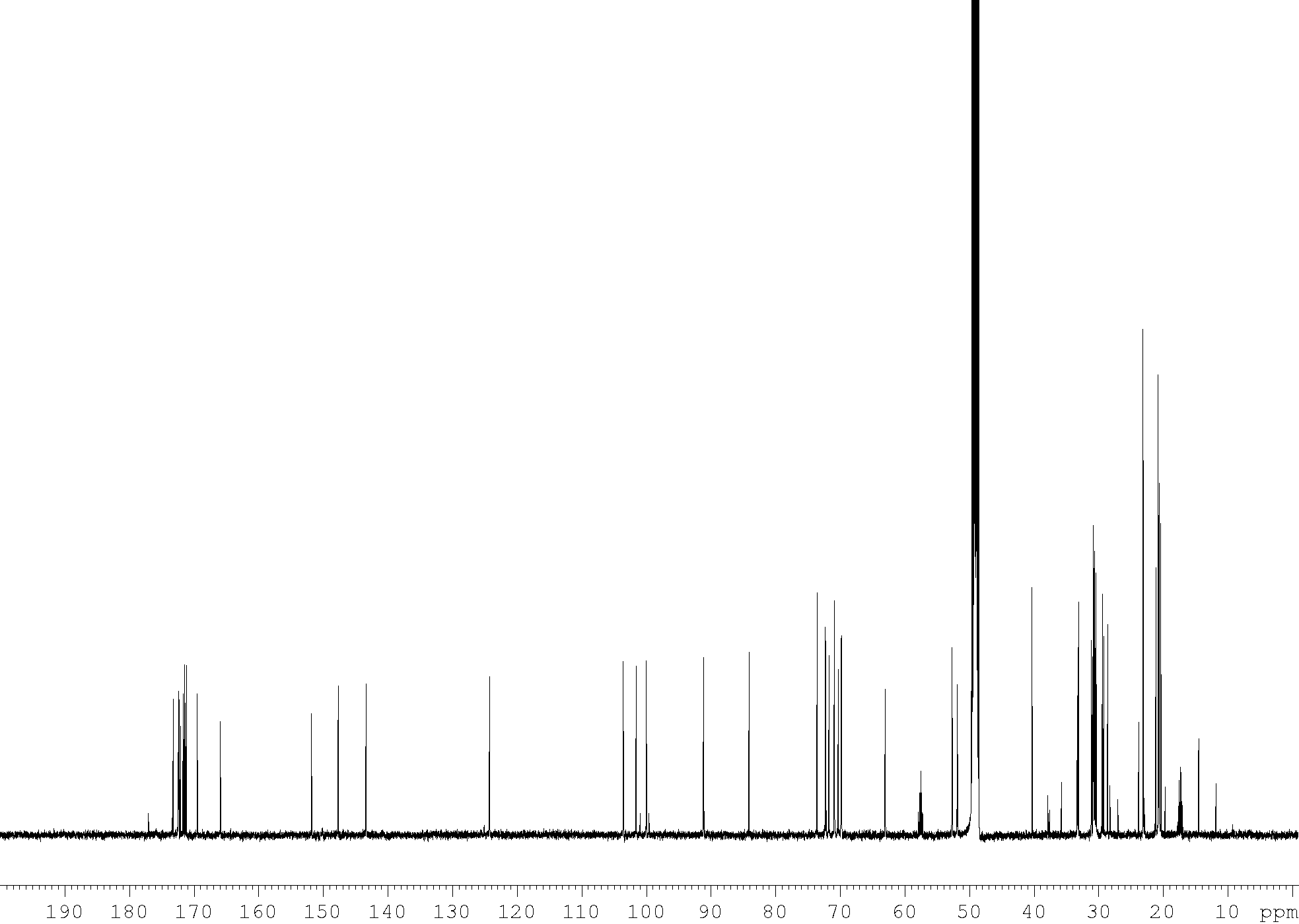


tunicamycin-8OAc-3Boc

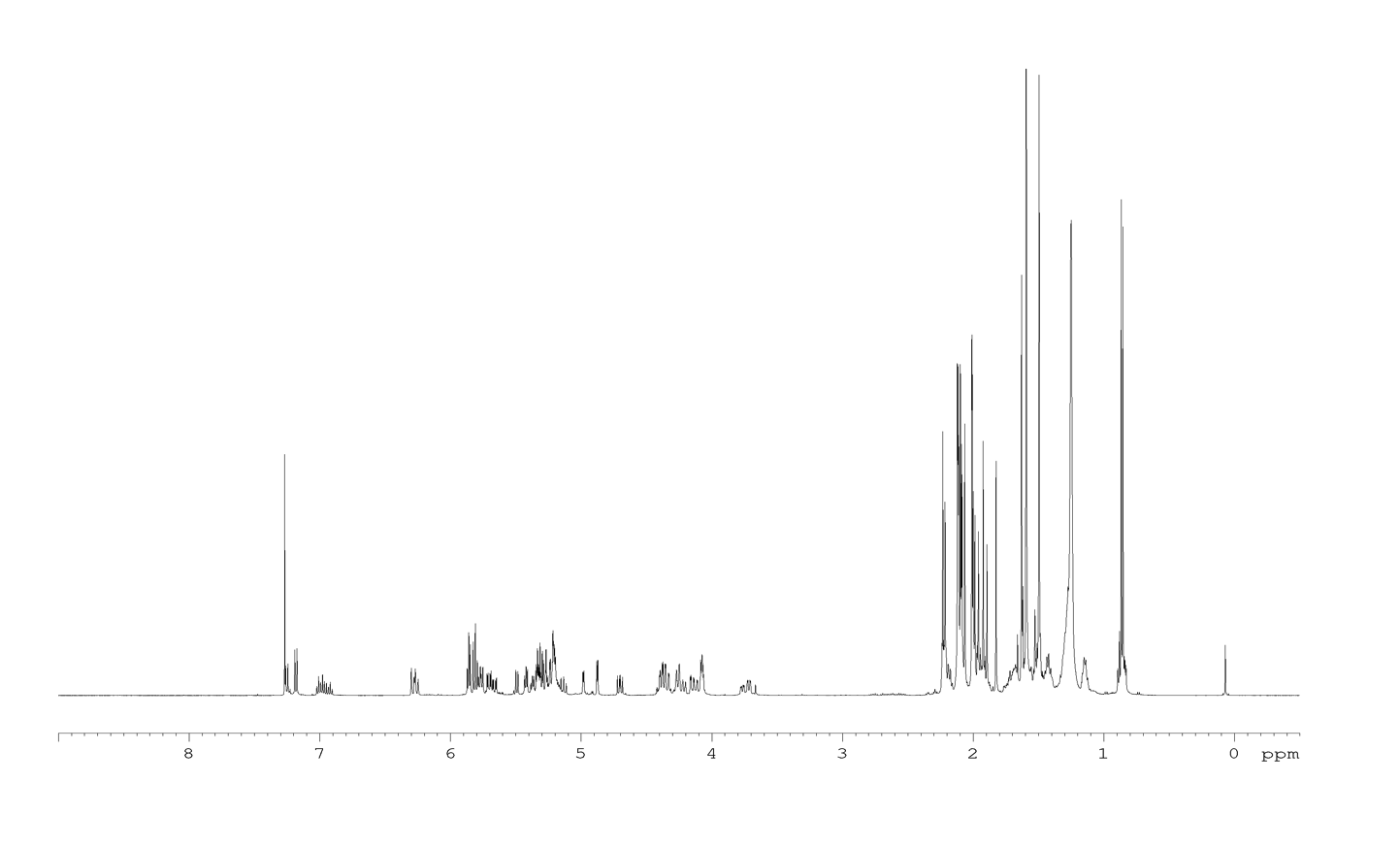


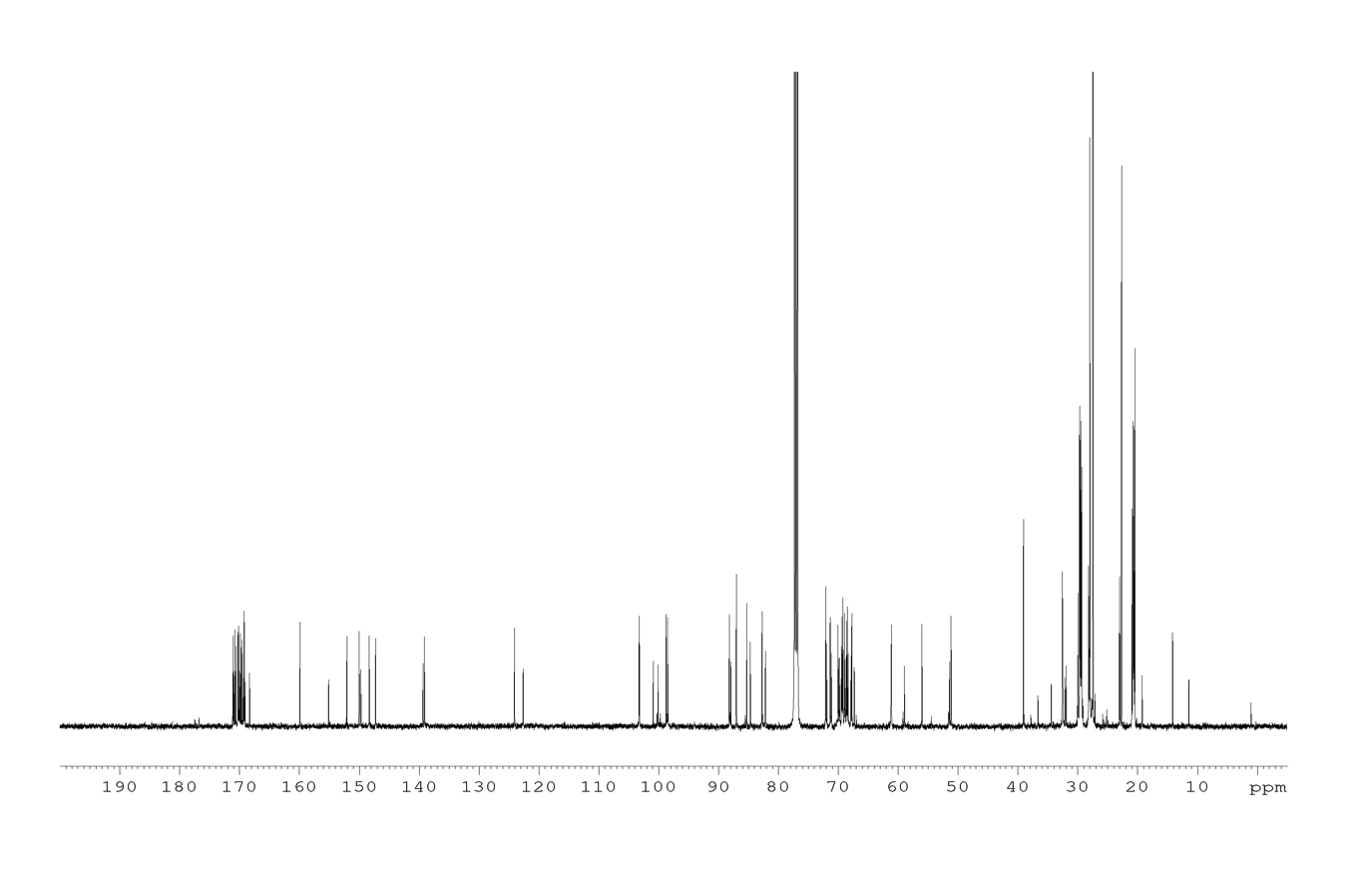


TUN-Boc,Boc

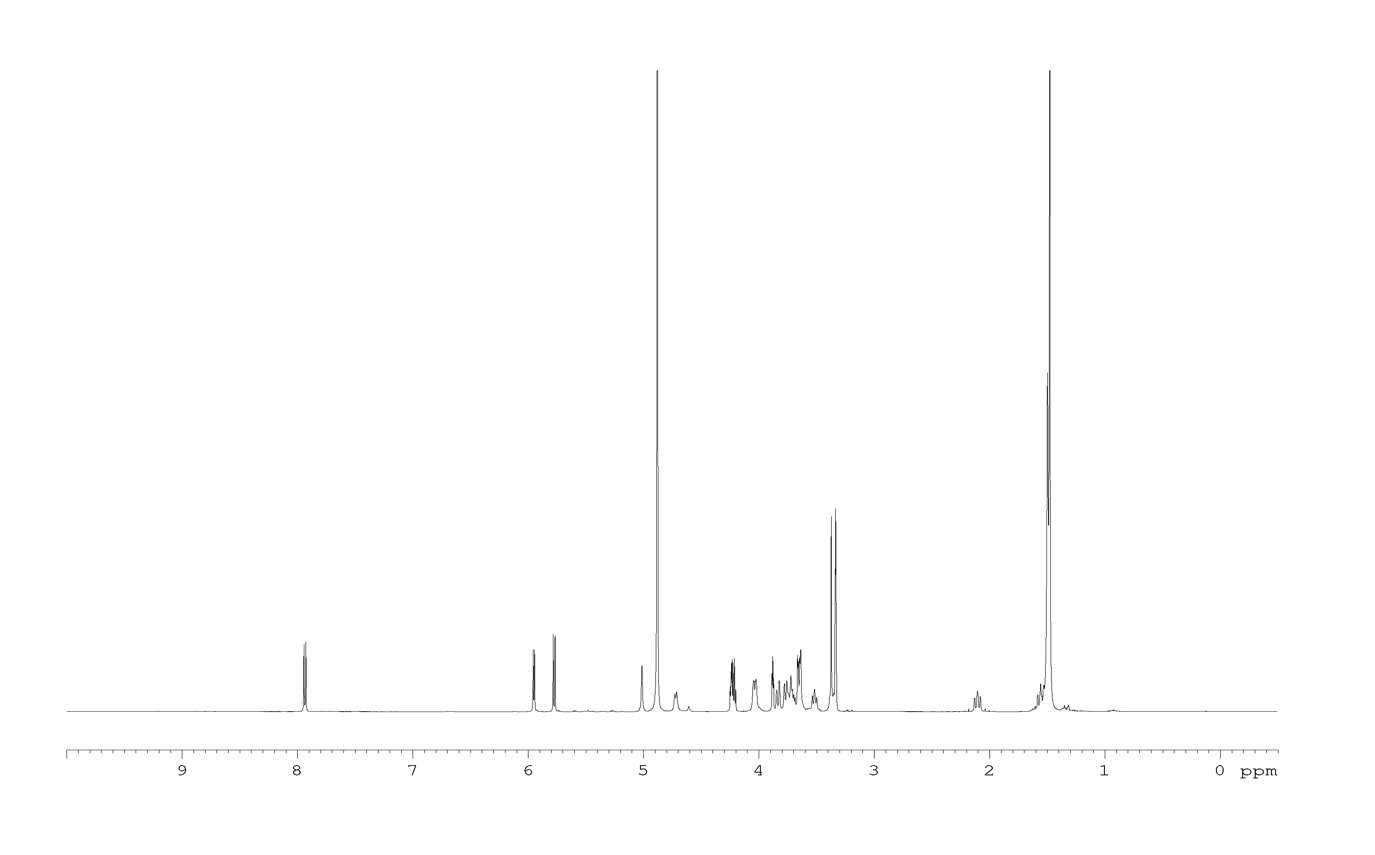


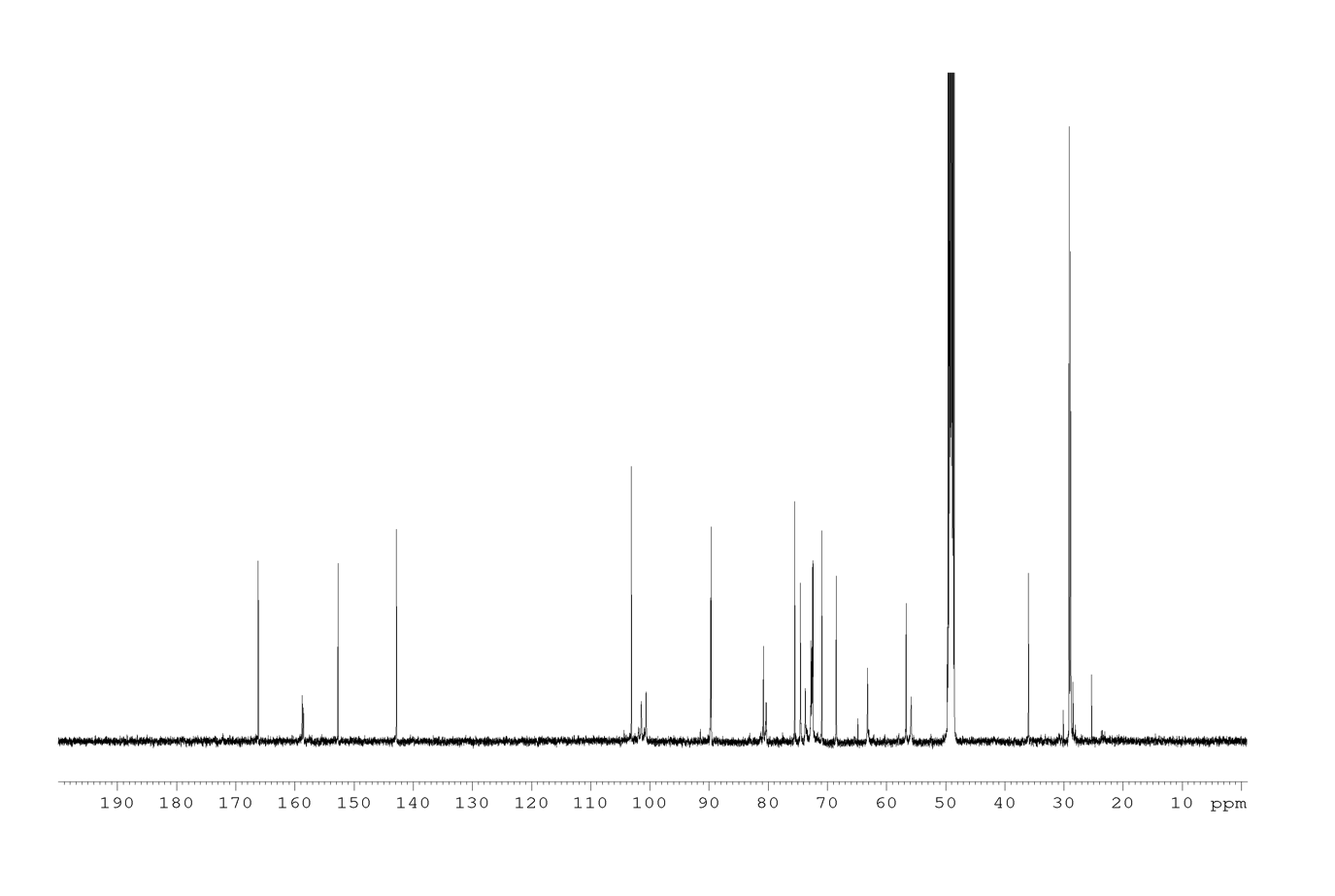


TUN-Ac,Ac

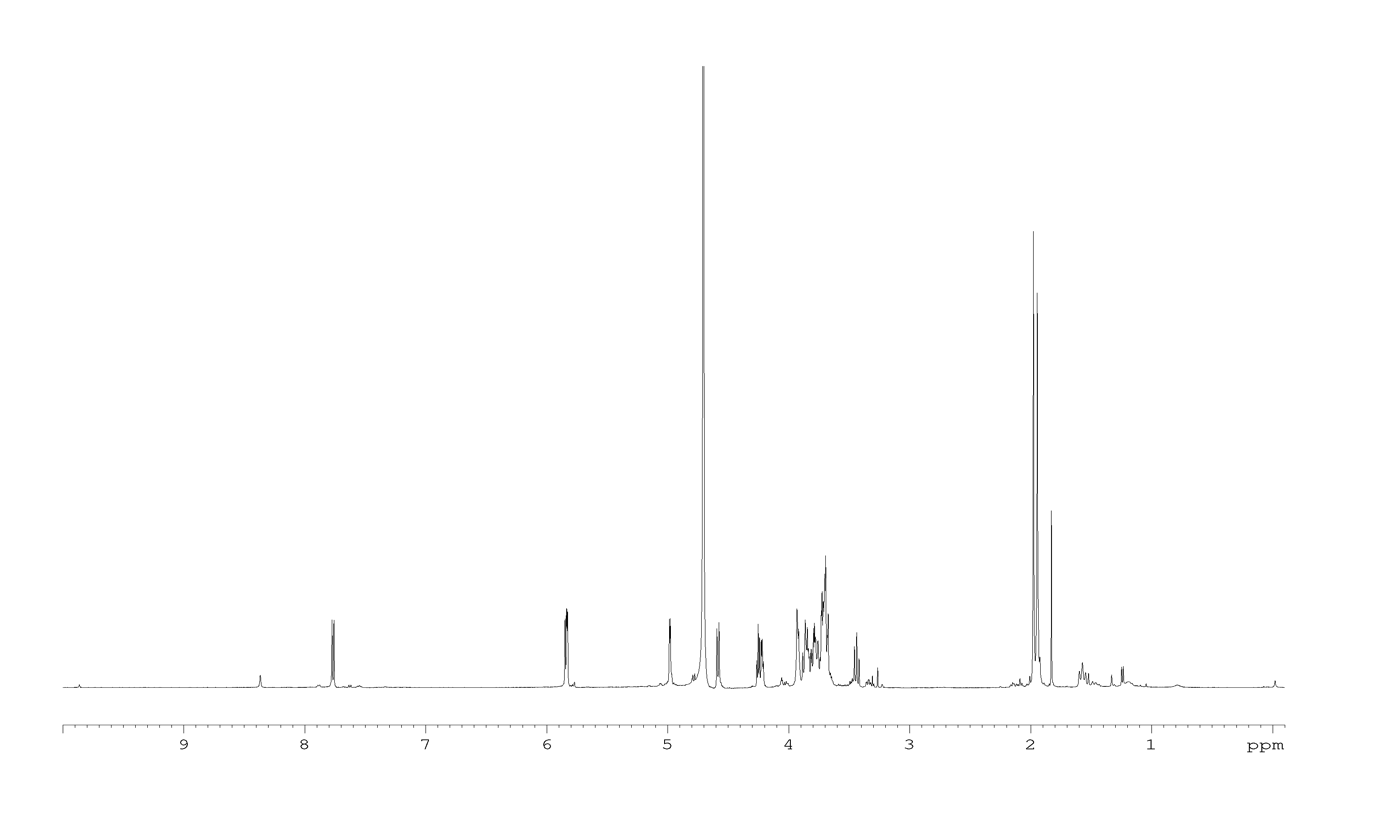


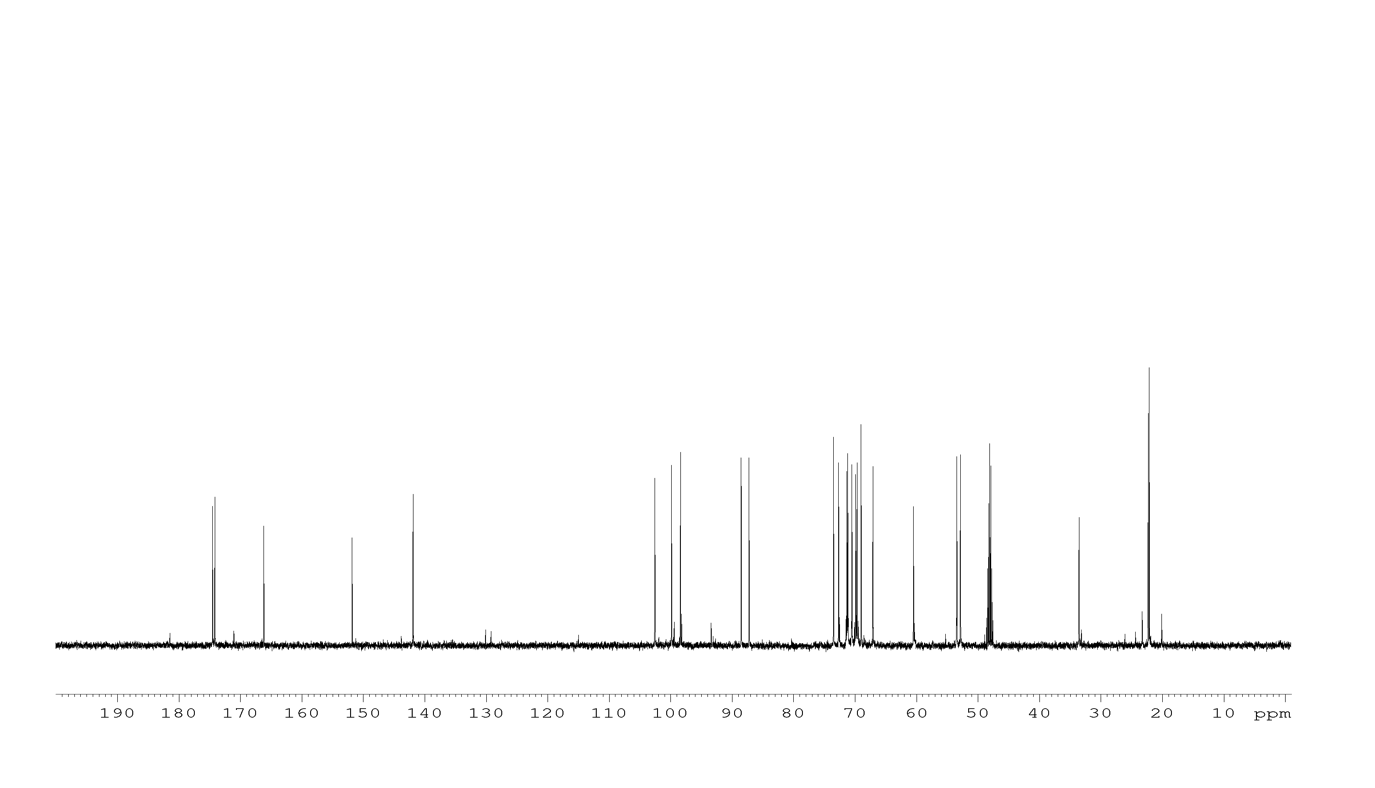


TUN

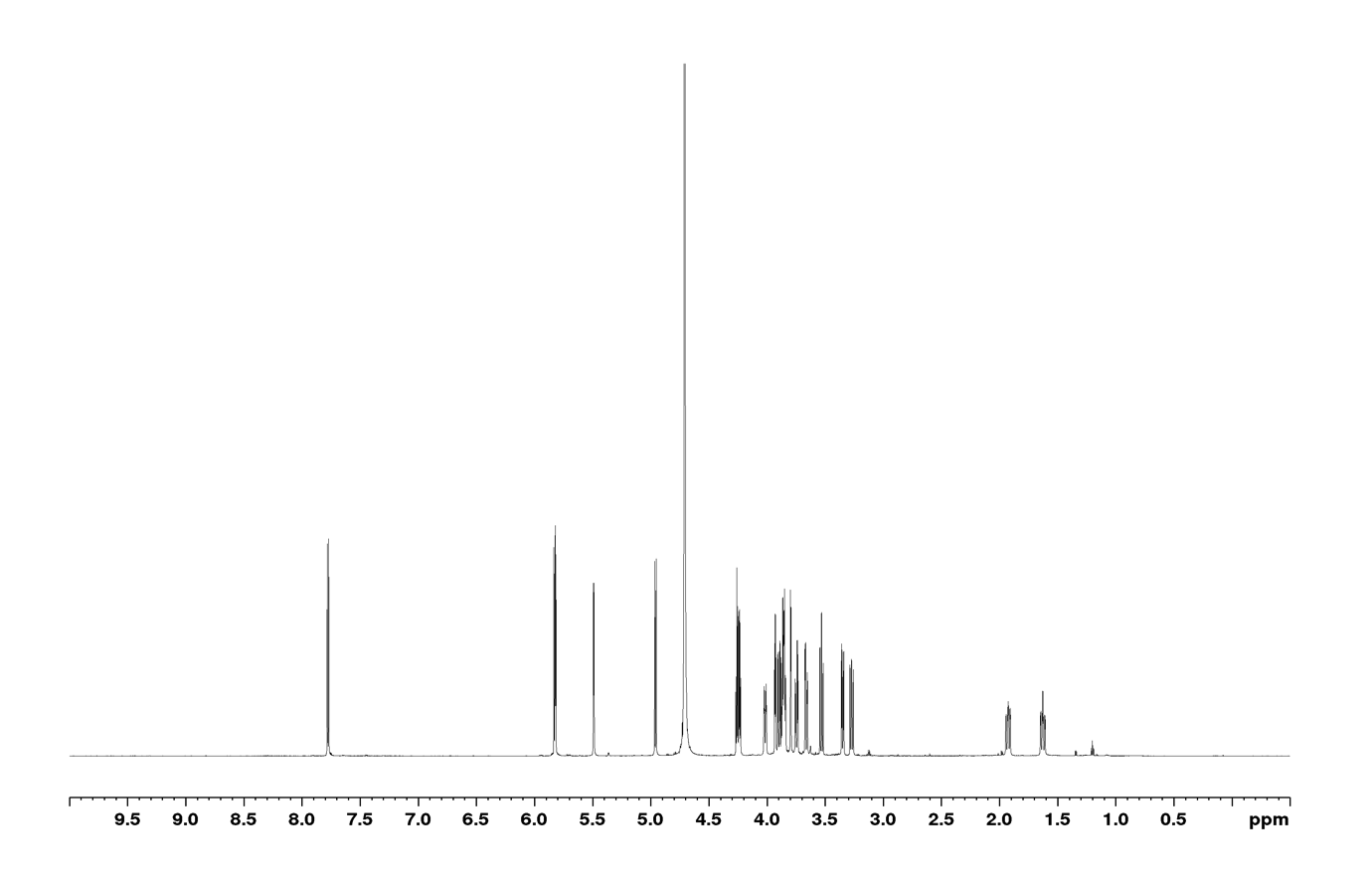


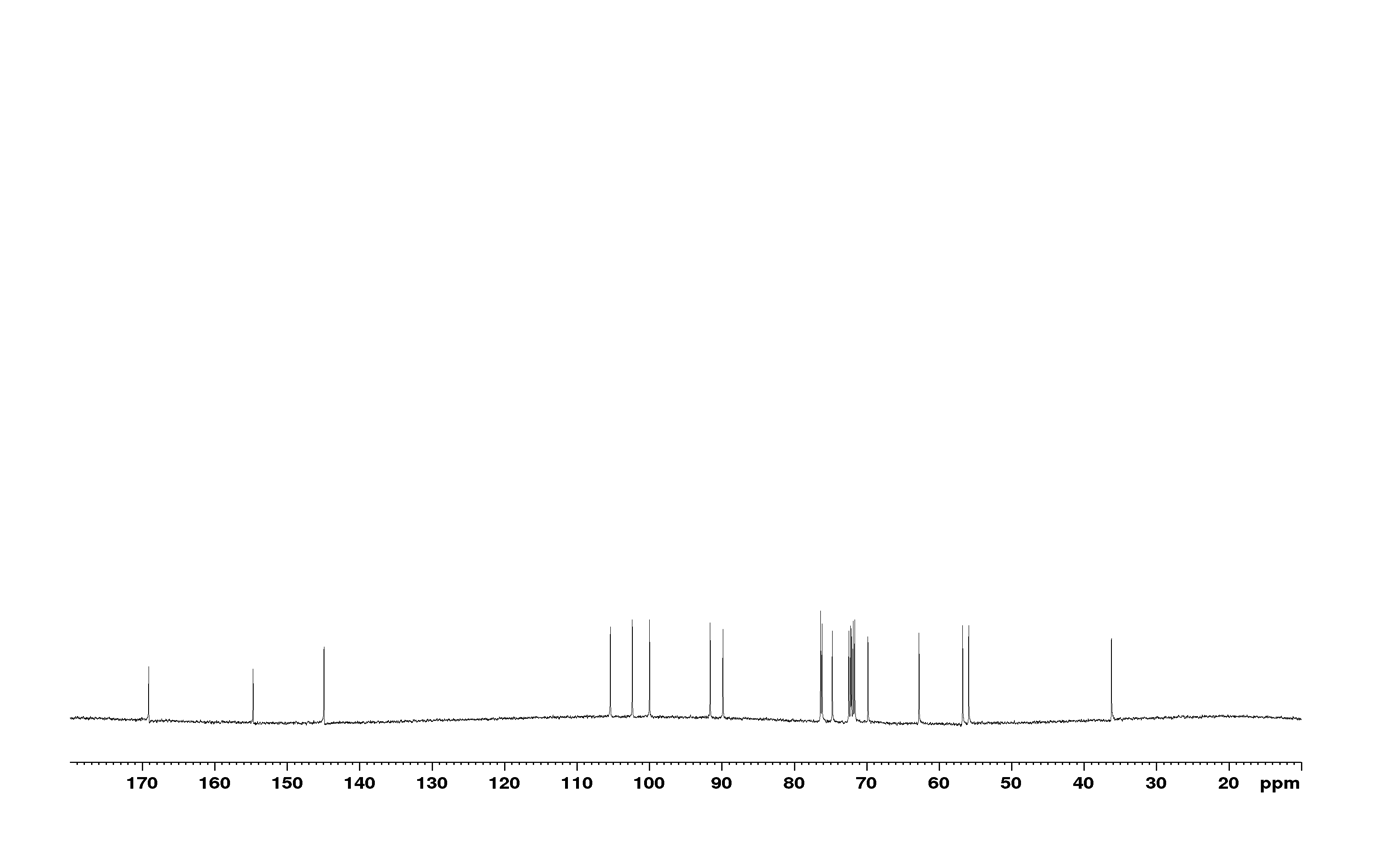


**TUN-7,7**

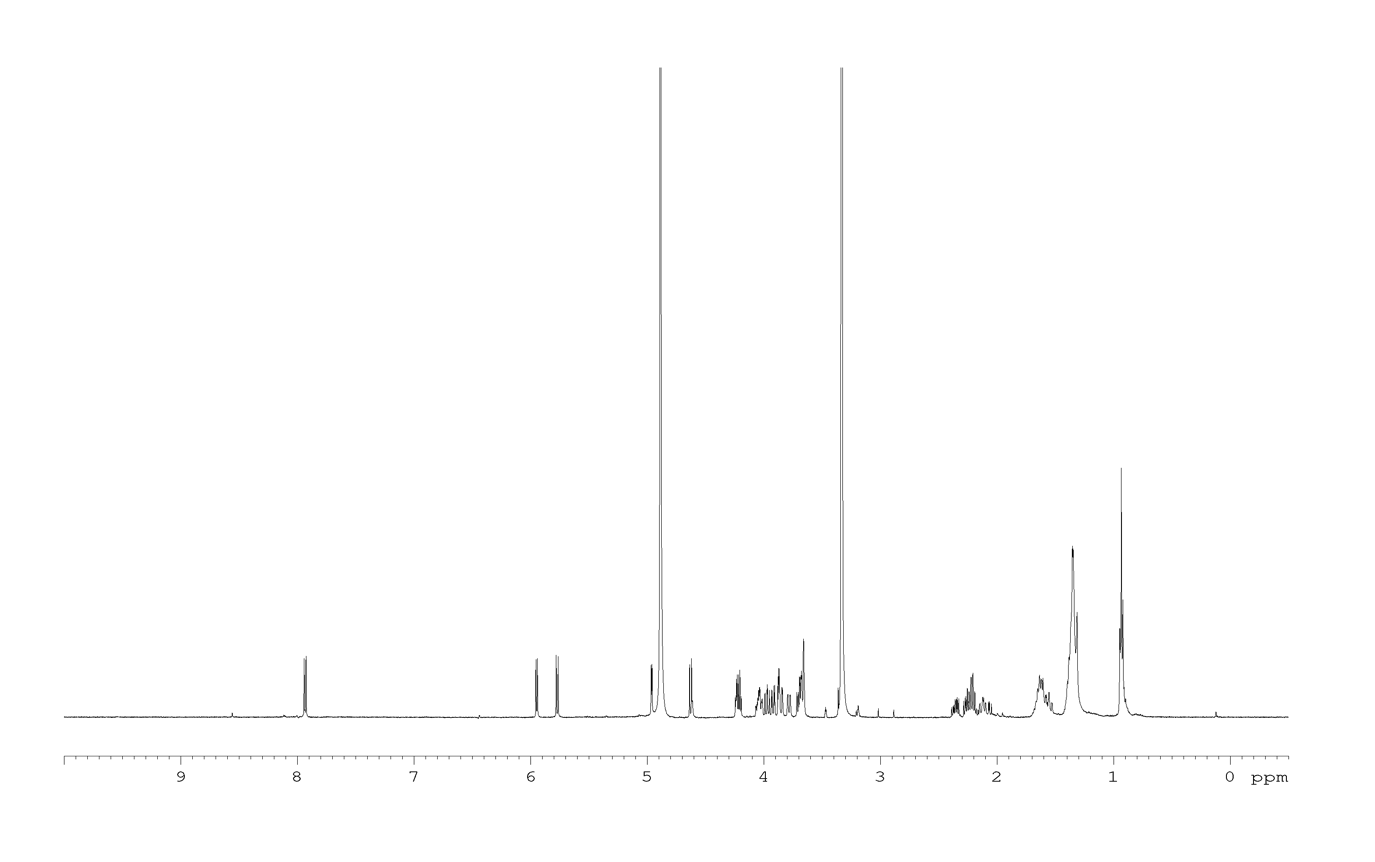


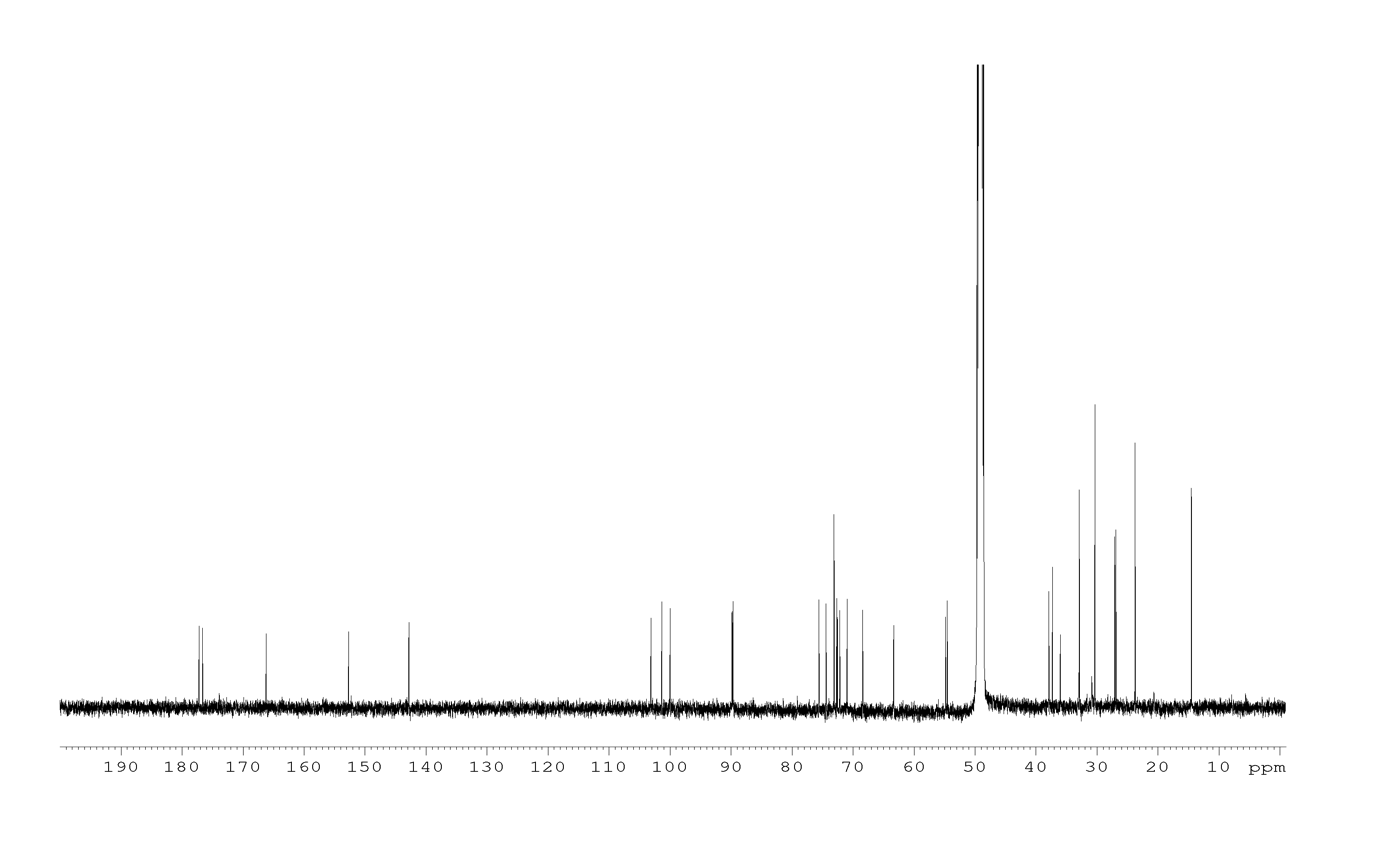


**TUN-8,8**

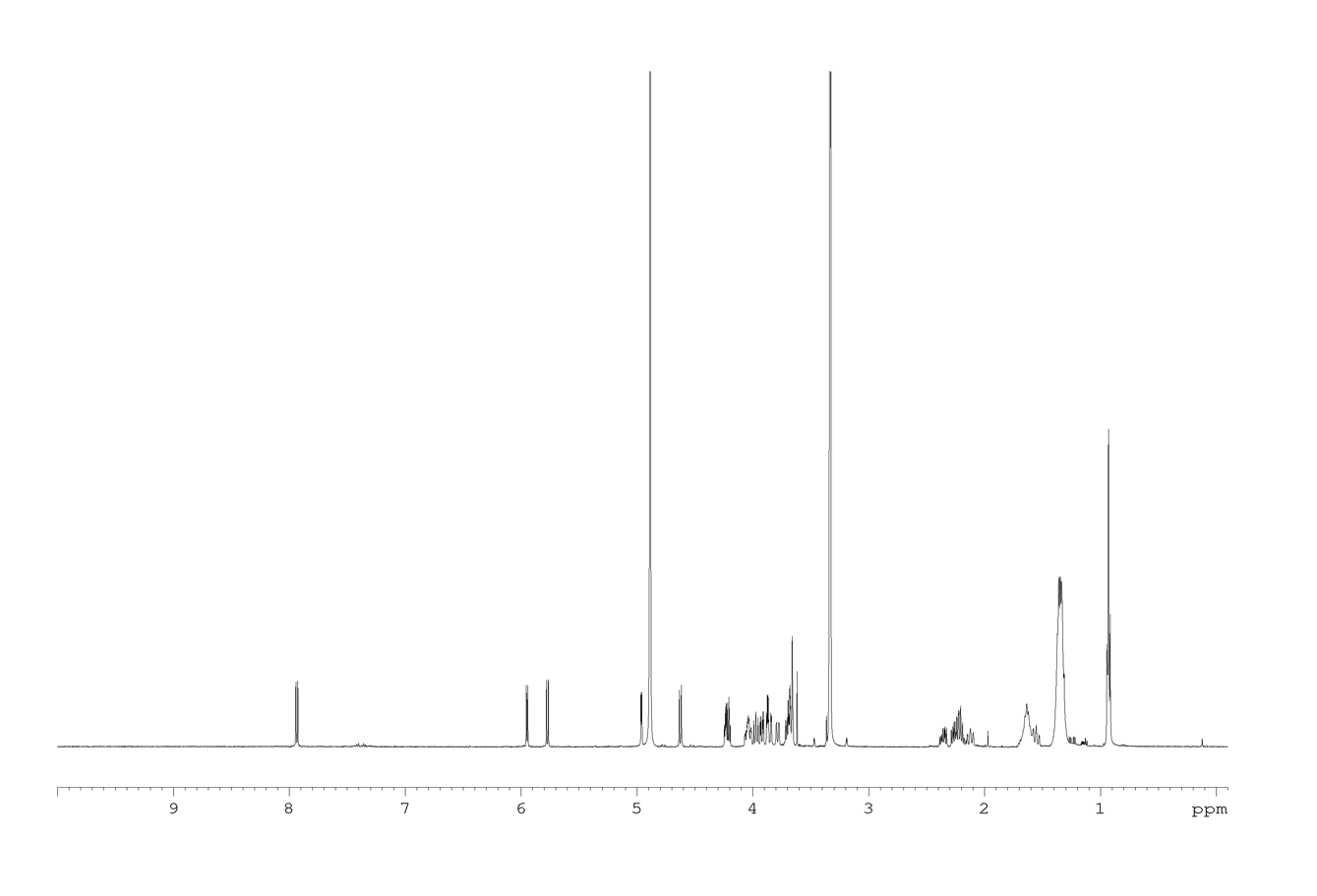


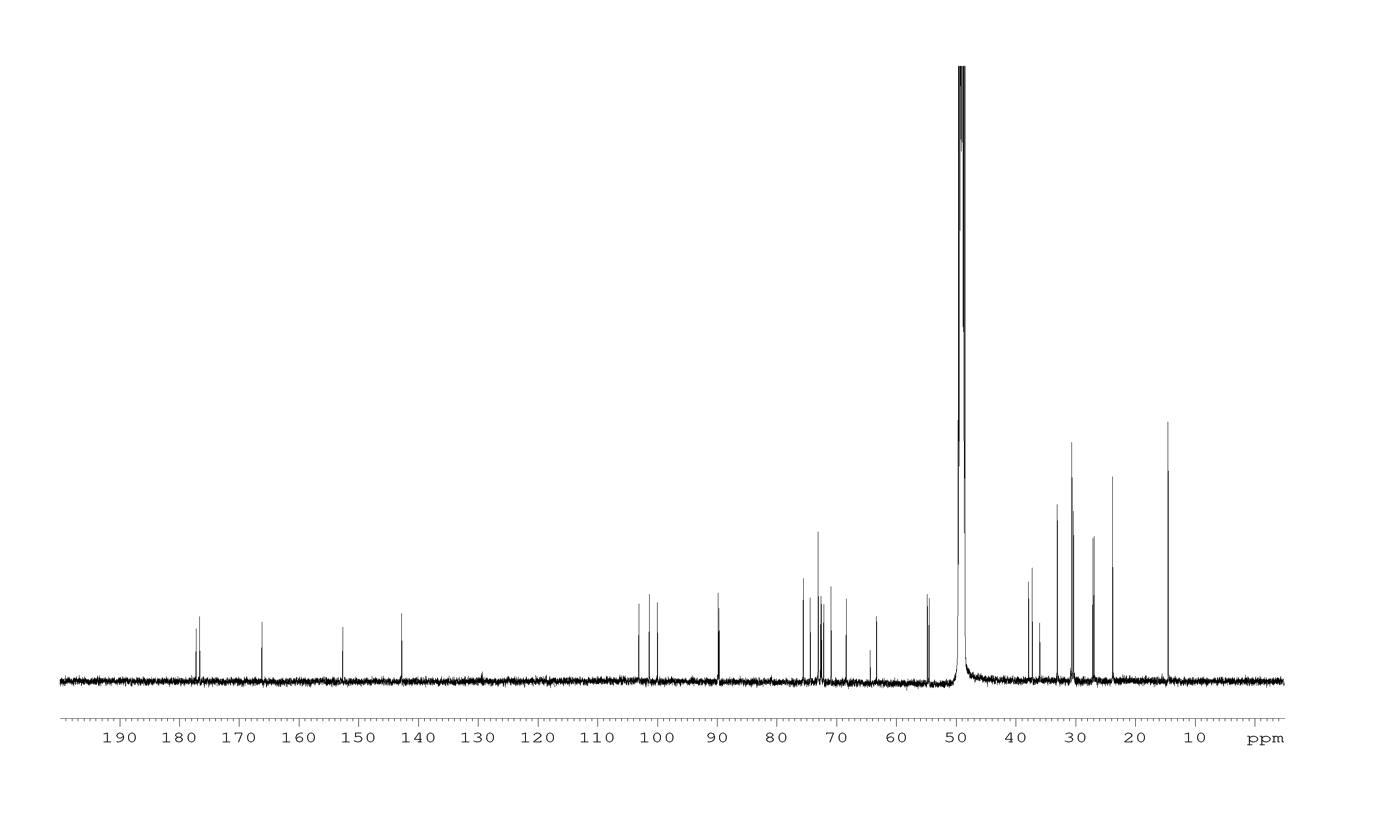


**TUN-9,9**

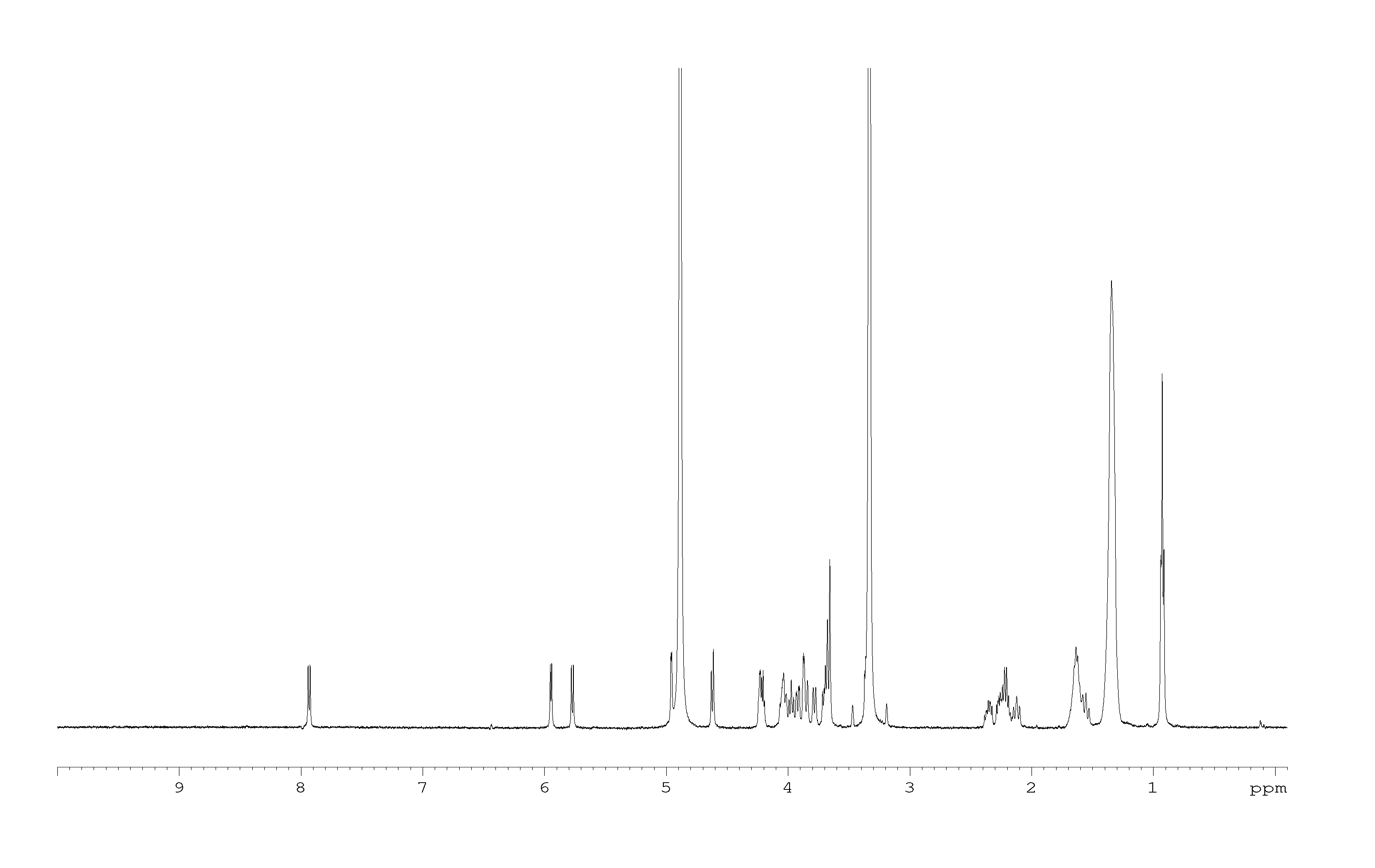


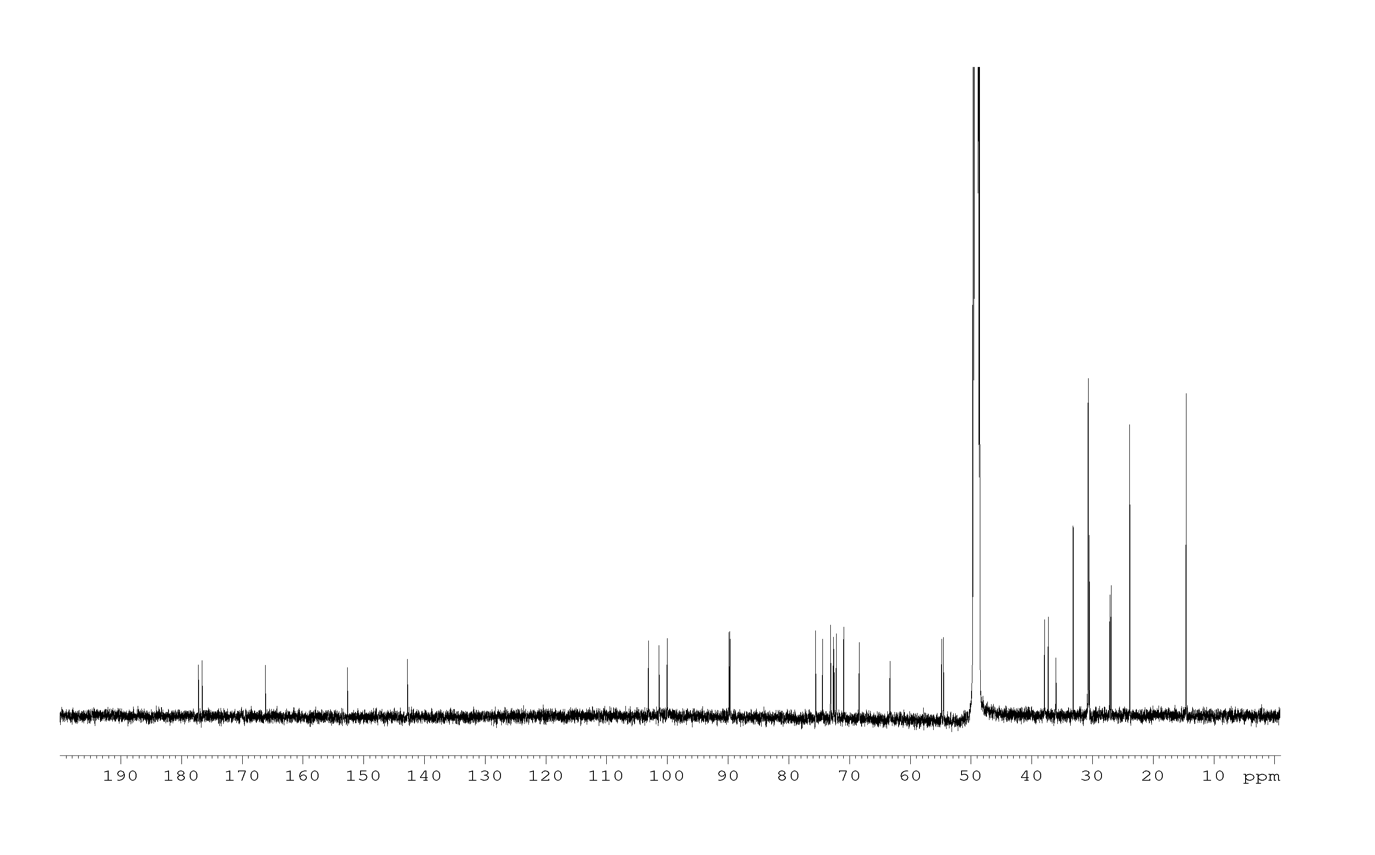


**TUN-10,10**

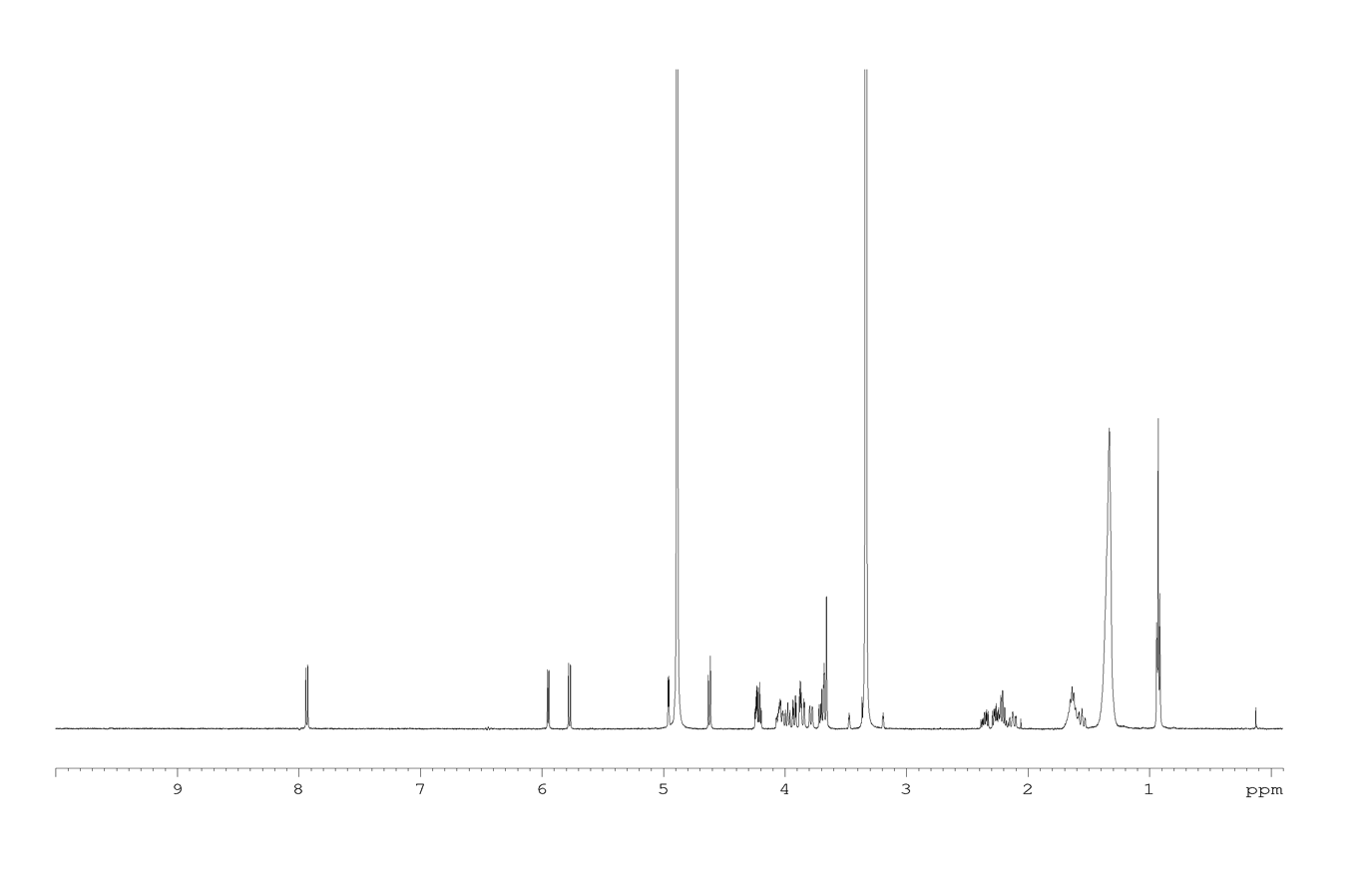


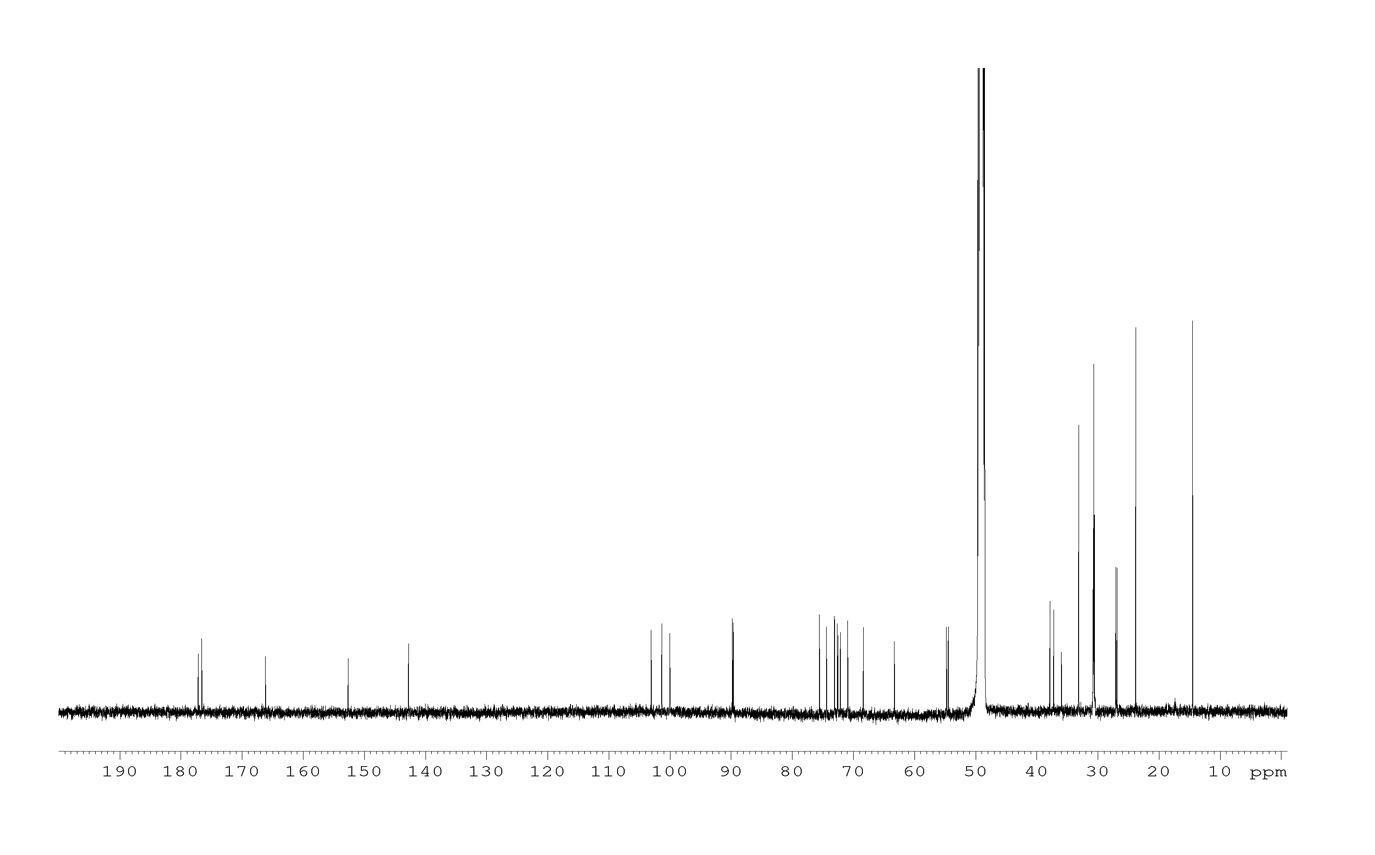


**TUN-11,11**

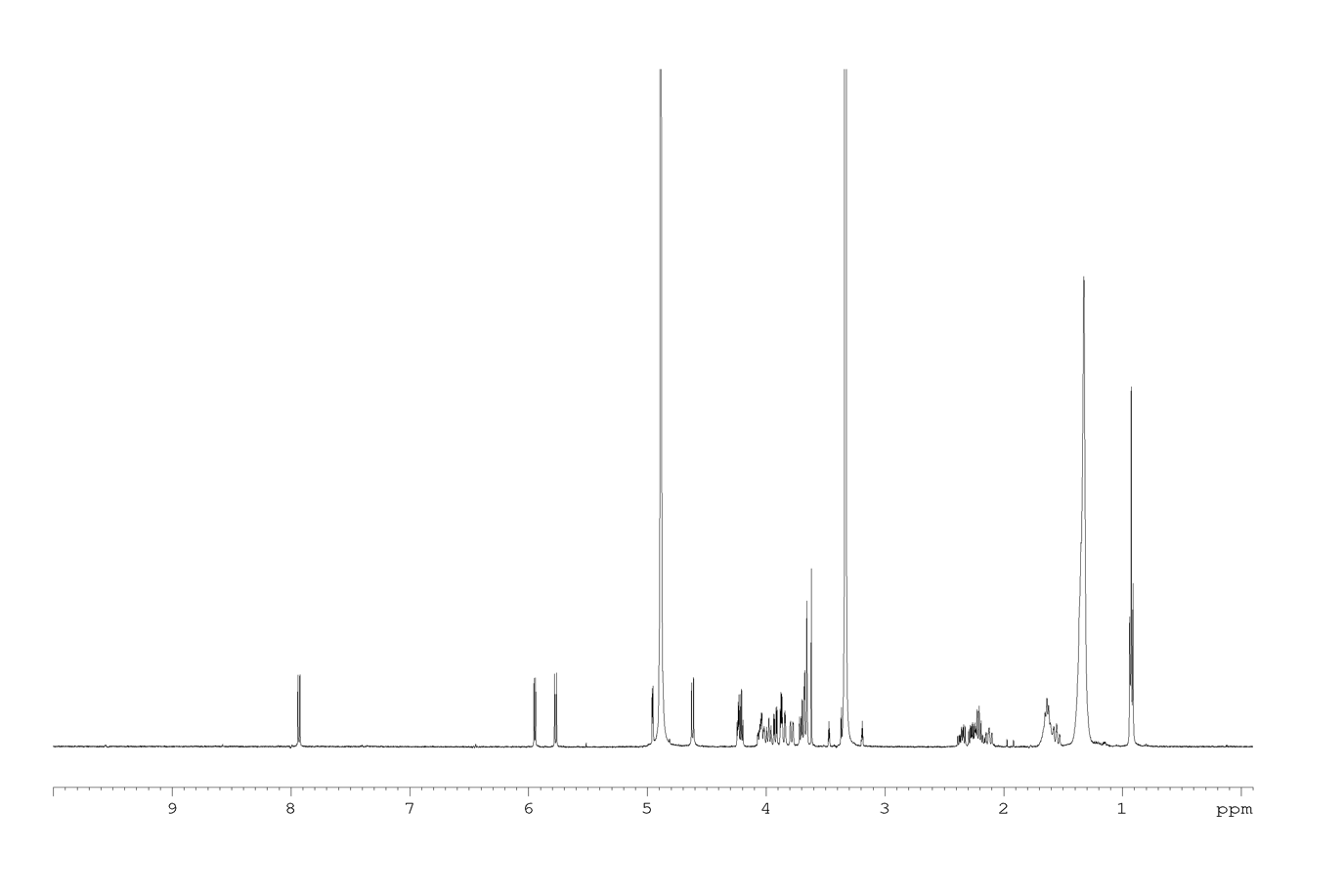

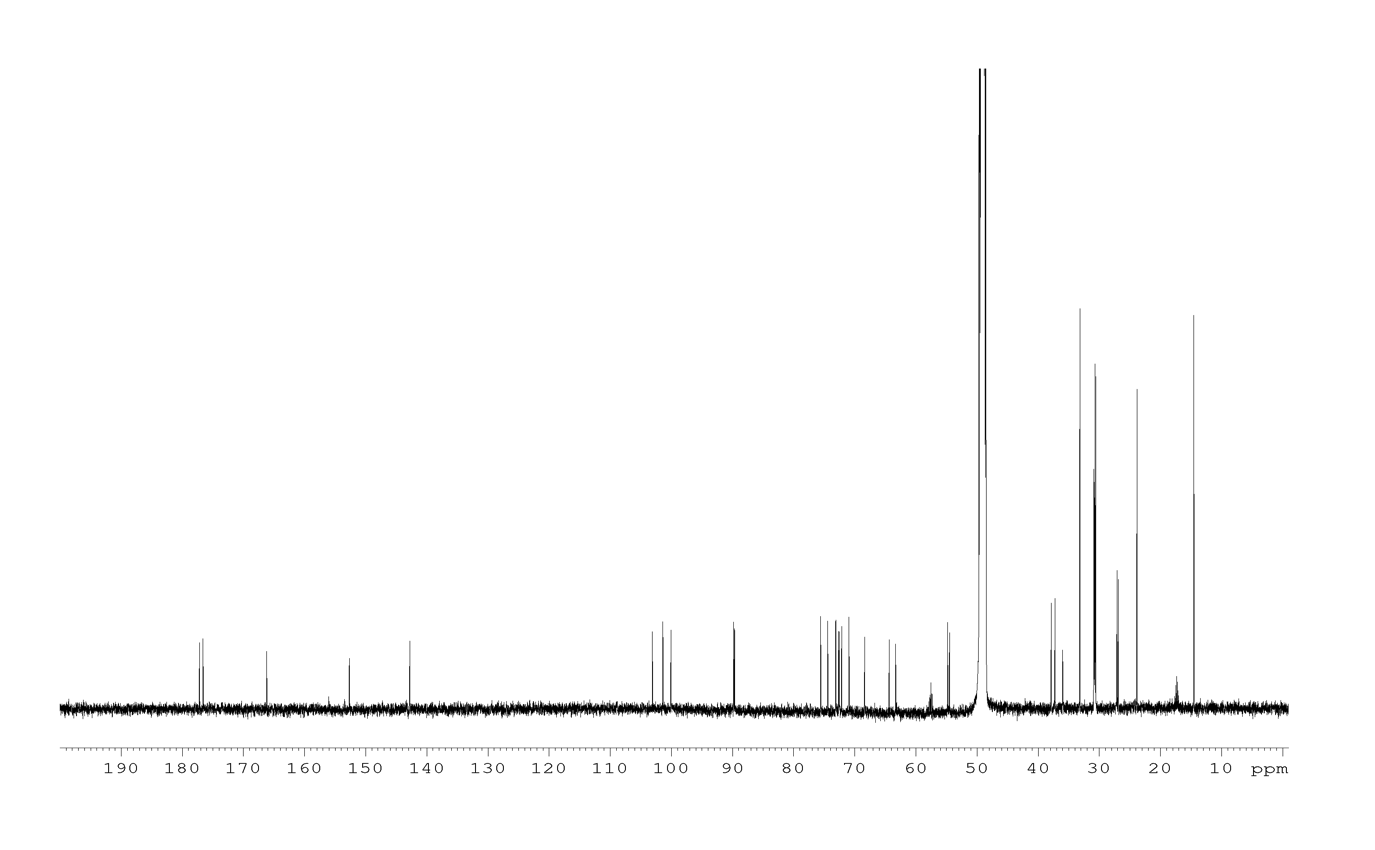


**TUN-12,12**
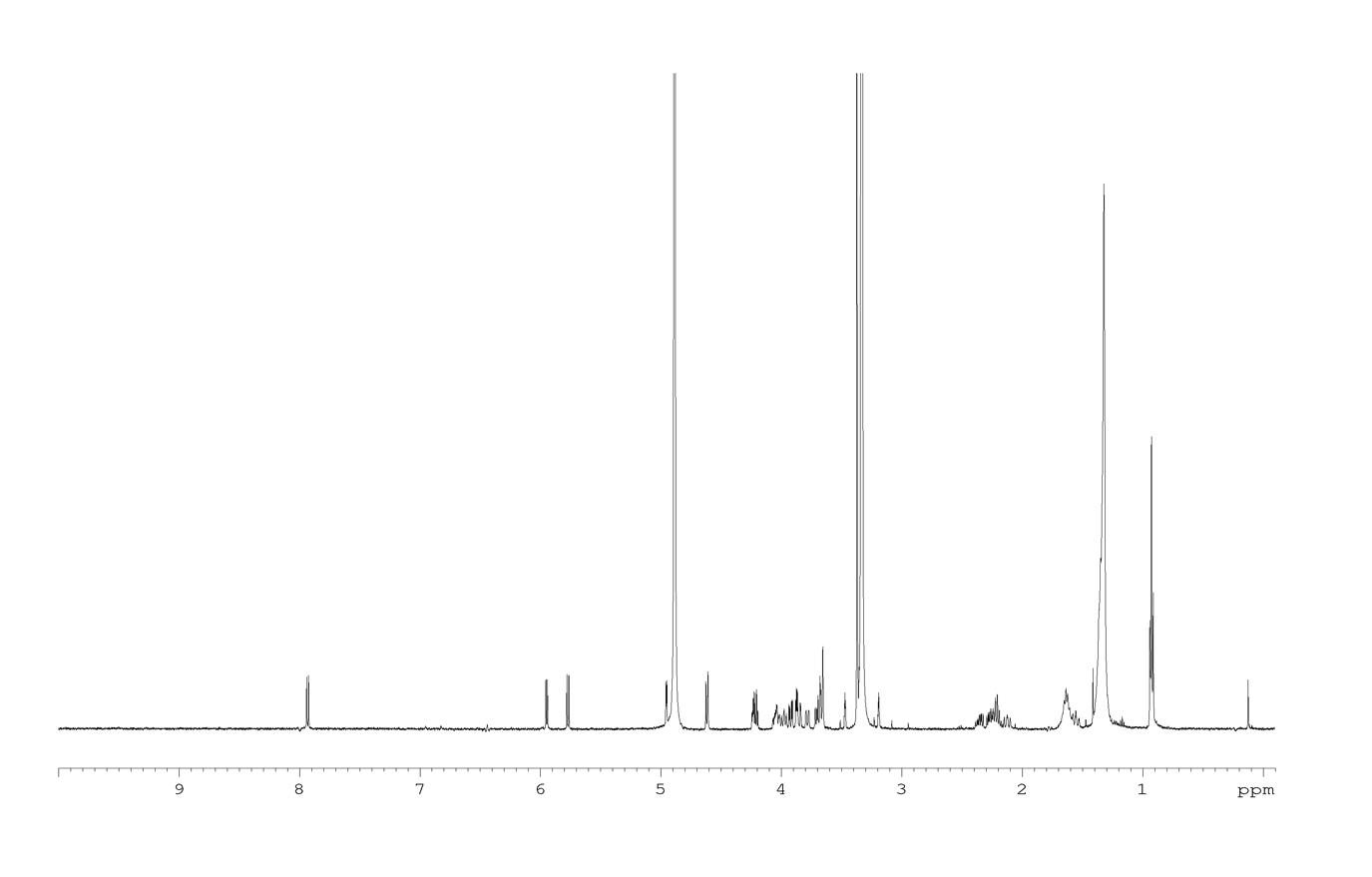

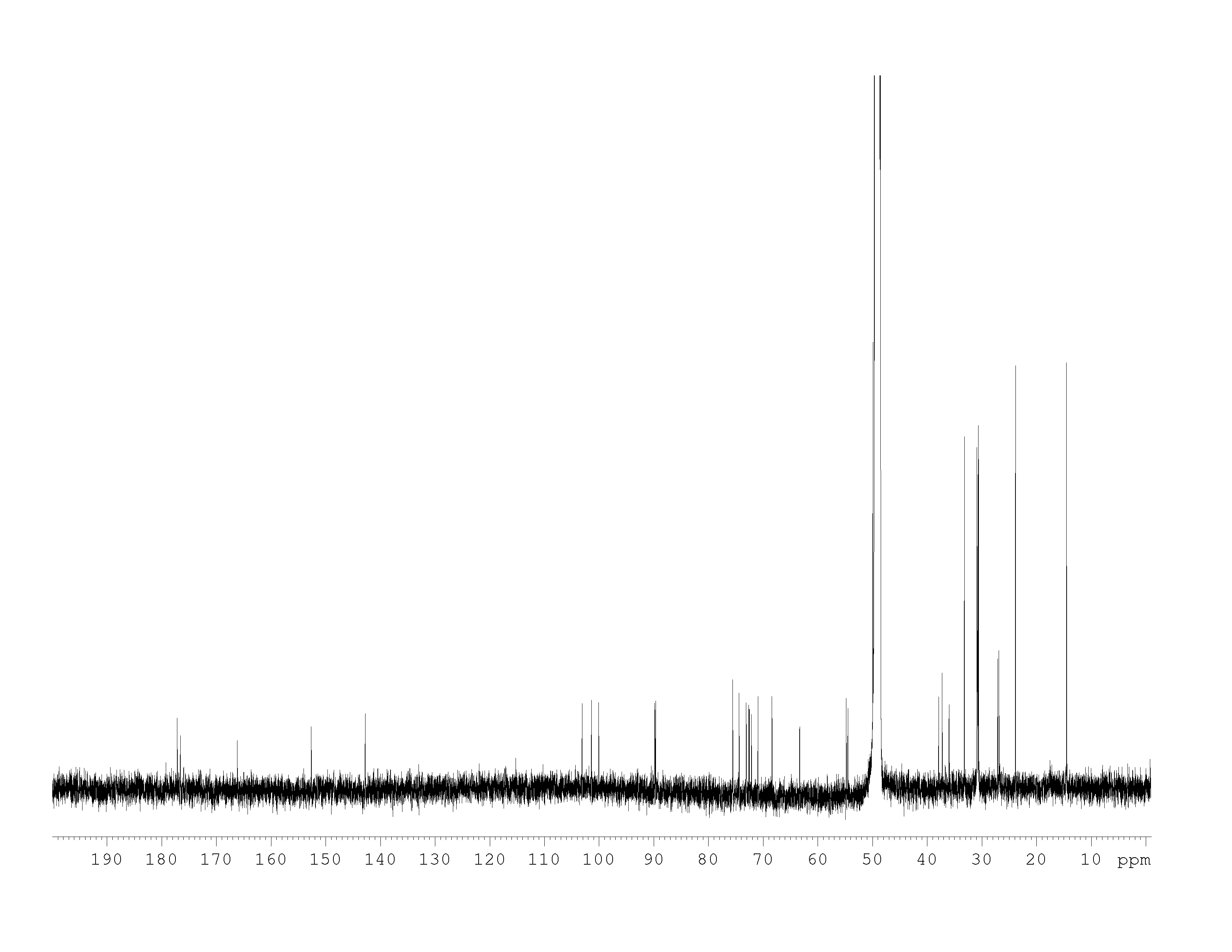


Heptaacetyl-tunicamyl-uracil

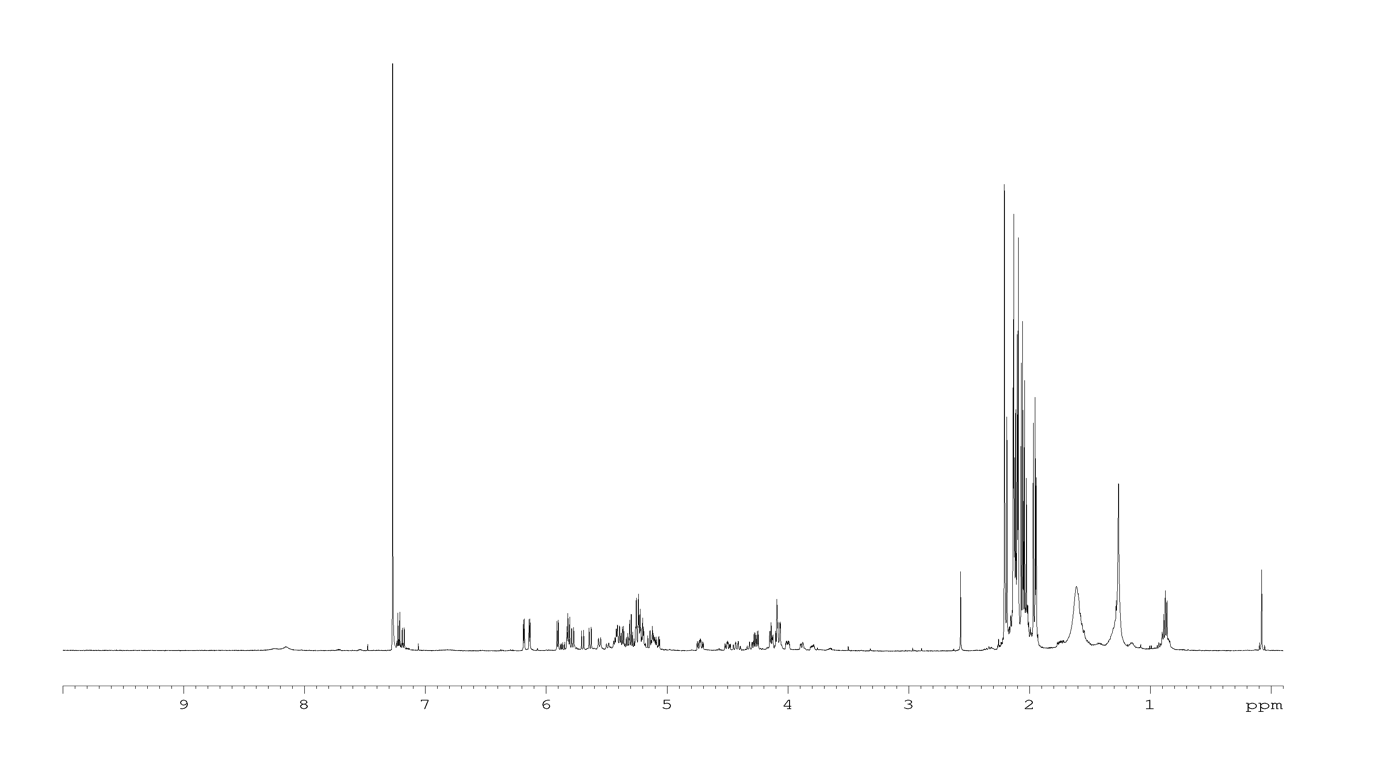


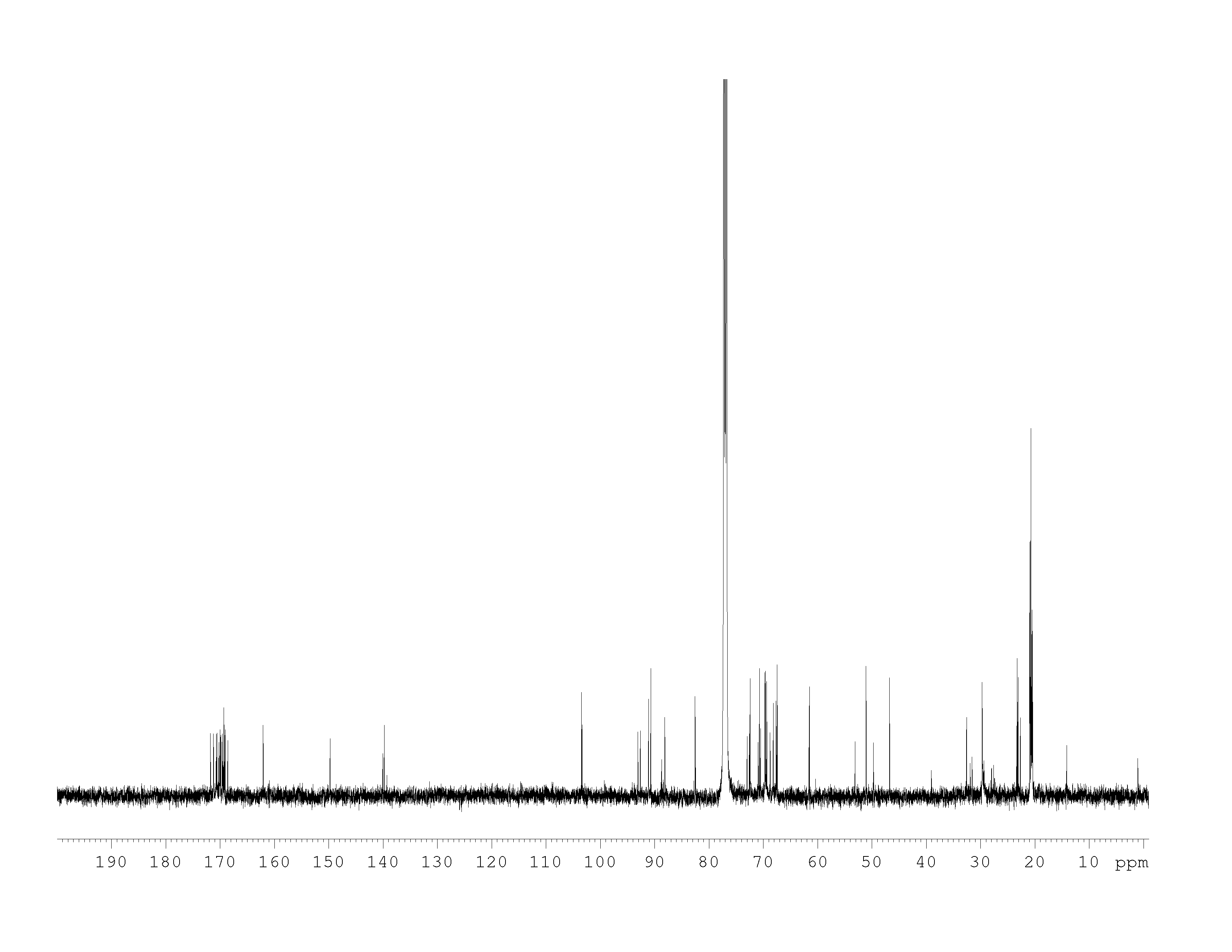


N-acetyl-tunicamyl-uracil

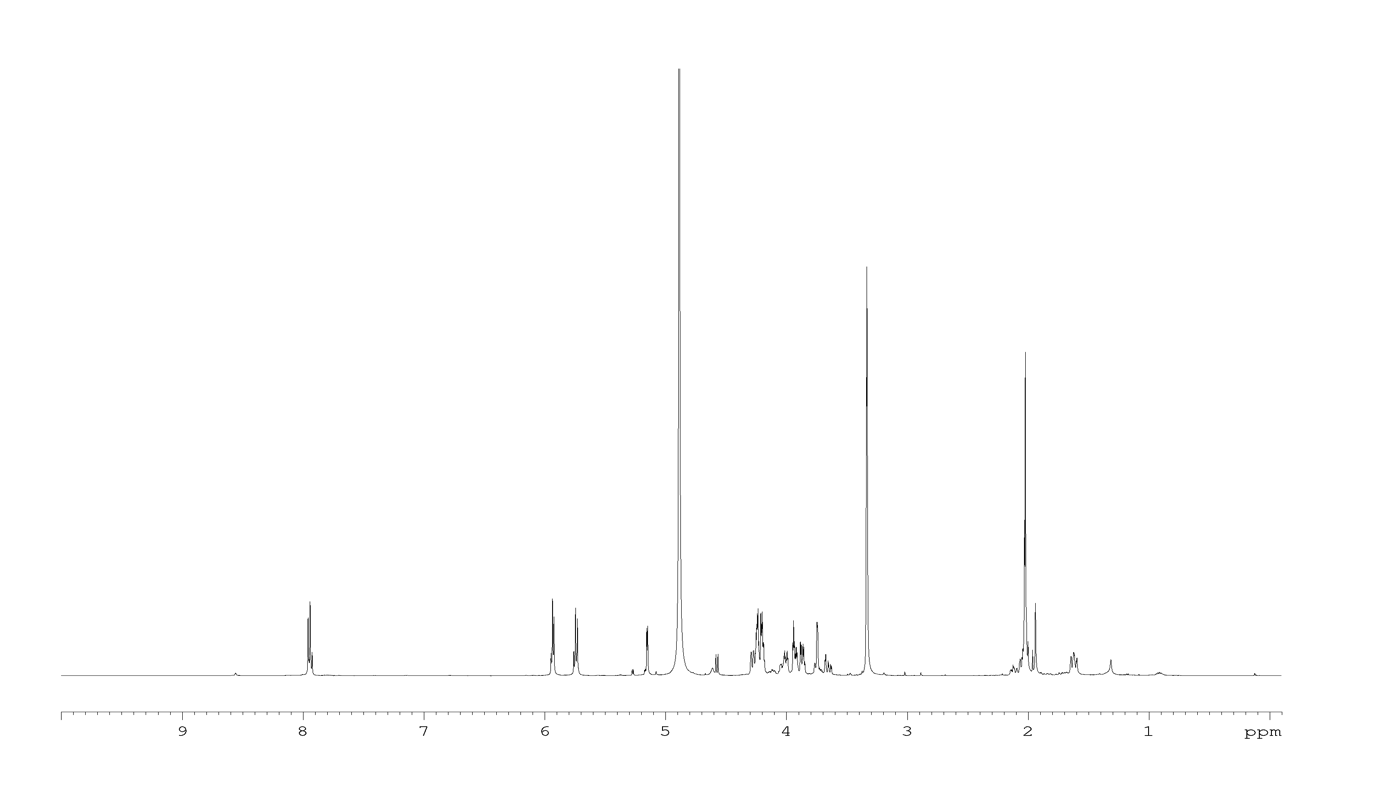


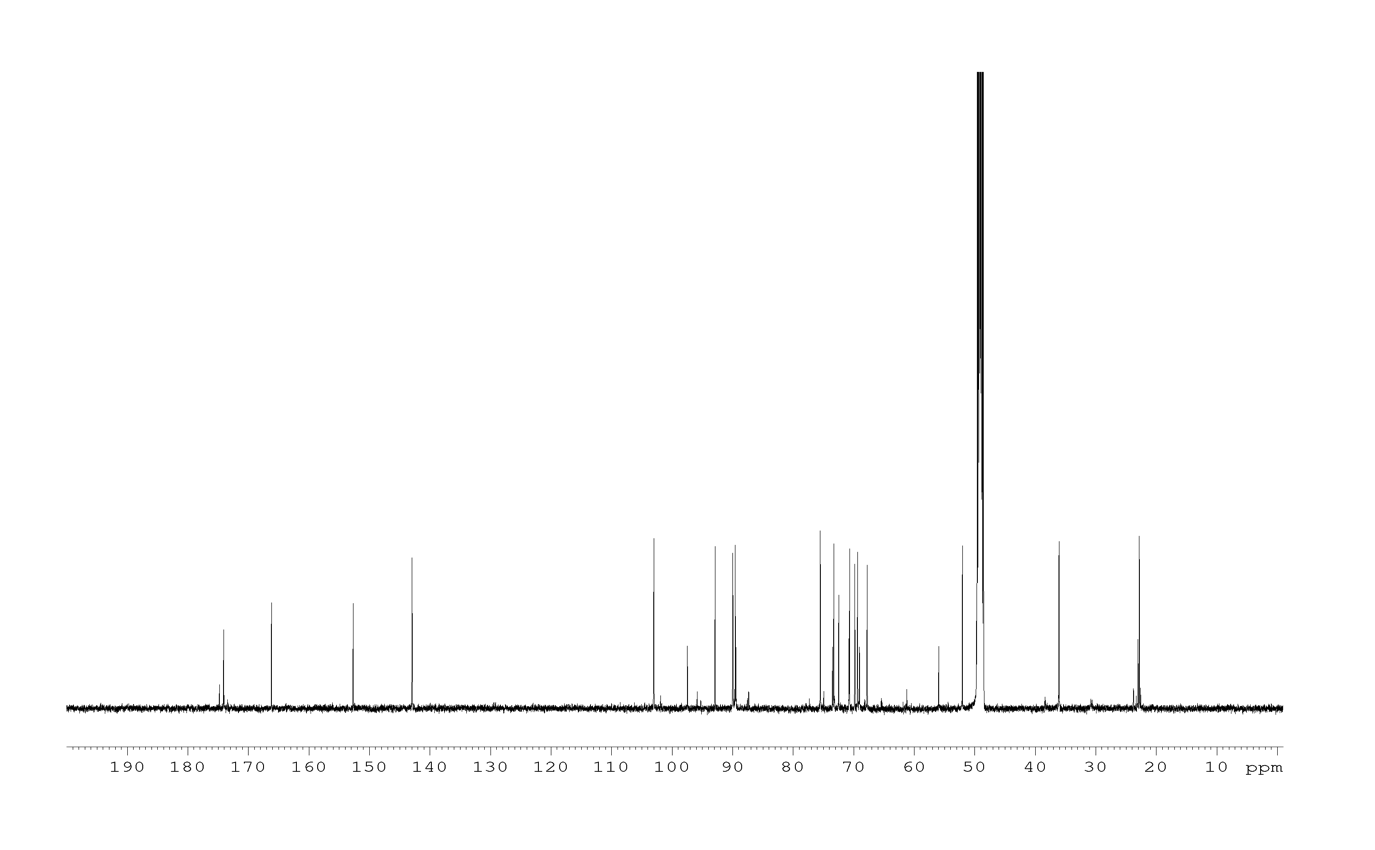


*N*-Octanoyl-*N’*-acetyl tunicamycin

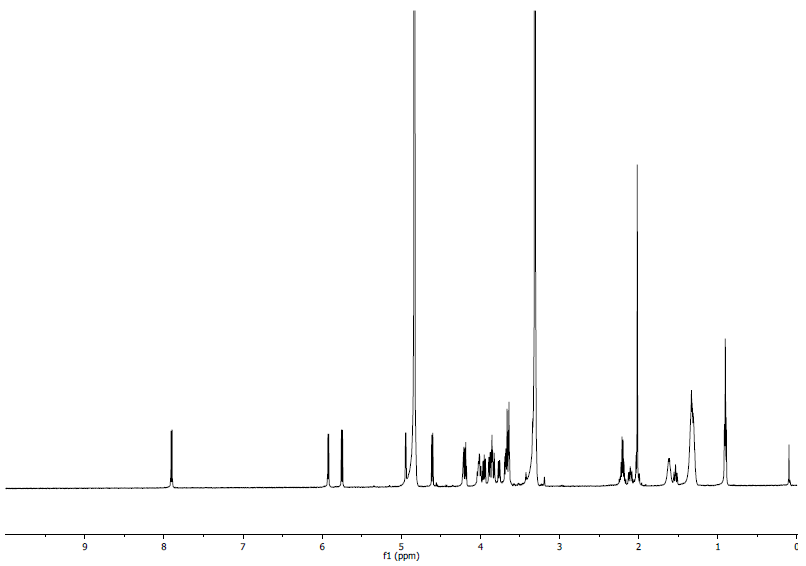


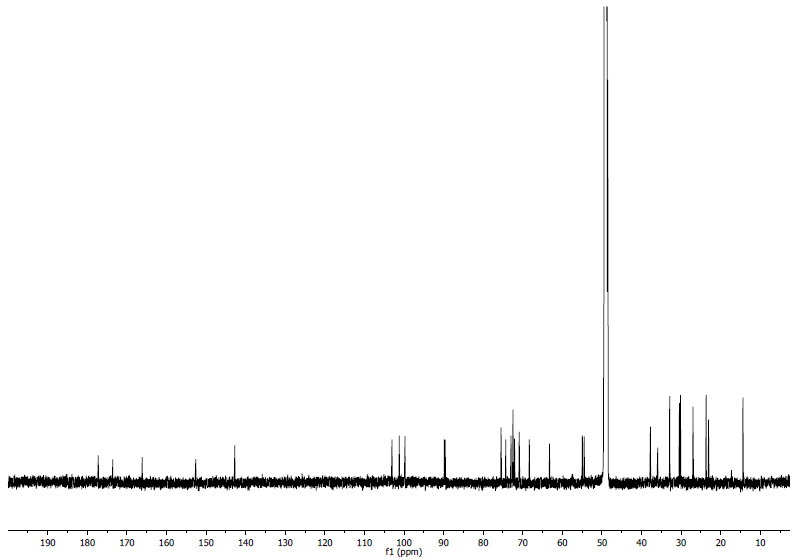

Supplement: Methods S1. PDF file containing methods for semi-synthetic synthesis of the TUN-X,X analogues and related compounds. Related to Figure 5 [file mmc1.docx]
