# Supplementary material for: Structures of DPAGT1 Explain Glycosylation Disease Mechanisms and Advance TB Antibiotic Design
Source: Cell. 2018 Nov 1;175(4):1045–1058.e16. doi: 10.1016/j.cell.2018.10.037 (PMC6218659; doi:10.1016/j.cell.2018.10.037)
Supplement: Document S1. Tables S1–S7, Related to Figures 1, 4, 5, 6, and 7 [file mmc2.pdf]

Supplemental tables

Table S1 Characterisation of DPAGT1 mutations found in patients with congenital myasthenic syndromes (CMS, shown in blue) and congenital disease of glycosylation type-Ij (CDG-Ij, shown in green). Lighter tone indicates variant with greater loss of function. Activity and thermostability data presented are means ± SD (n=9). Related to Figure 4.

Patient	Mutation	Reference	protein yield (mg/L)	Activity	Thermostability ($Tm_{1/2}$)					Comment
					Apo	+DolP	+UDPGlcNAc	+UMP	+ TUN	
	**WT**		0.62±0.19	100.00%	51.7±0.2	58.5 ± 0.3	55.1±0.4	51.7±0.2	83.0±0.4	WT
CMS 1	Leu120Met	Belaya *et al.* 2012, Yuste-Checa *et al.* 2017	0.16±0.04	19.3±3.6	51.3±0.8	-	-	-	-	19% WT activity, Abnormal splicing
CMS 1	Val264Gly	Belaya *et al.* 2012, Yuste-Checa *et al.* 2017	0.52±0.23	252.0±32.5	50.4±0.3	57.2±0.2	54.6±0.4	50.5±0.2	82.6±0.1	Abnormal splicing , 2.5 fold increase in activity
CMS 2	Gly192Ser	Belaya *et al.* 2012	0.04±0.005	10.9±6.2	47.0±0.5	-	-	-	-	11% WT activity
CMS 2	Gly160Ser	Belaya *et al.* 2012	0.24±0.10	173.8±7.6	52.3±0.9	-	-	-	-	Abnormal splicing , 1.7 fold increase in activity
CMS 3	Met108Ile	Belaya et al. 2012	0.02±0.01	23.9±5.6	51.5±0.6	-	-	-	-	24% WT activity
CMS 3	Val117Ile	Belaya et al. 2012	0.08±0.05	58.6±9.8	53.6±0.1	-	-	-	-	60% of WT activity
CMS 4	Thr234Hisfs*116	Belaya *et al.* 2012	-	-	-	-	-	-	-	Truncation, no activity
CMS 4	Val117Ile	Belaya *et al.* 2012	0.08±0.05	58.6±9.8	53.6±0.1	-	-	-	-	60% of WT activity
CMS 5	Ile29Phe	Klein *et al.* 2015	0	-	-	-	-	-	-	Unable to purify protein
CMS 5	Pro30Ser	Klein *et al.* 2015	0.17±0.07	48.8±9.0	52.5±0.6	-	-	-	-	50% WT activity
CMS 6	Leu120Leu	Selcen *et al.* 2014	-	-	-	-	-	-	-	Abnormal splicing
CMS 6	Val264Met	Selcen *et al.* 2014	0.29±0.05	82.2±14.9	51.8±0.6	-	-	-	-	80% of WT activity
CMS 7	Arg218Trp	Basiri *et al.* 2013	0	-	-	-	-	-	-	Unable to purify protein
CMS 8	Met1Leu	Selcen *et al.* 2014	-	-	-	-	-	-	-	Not yet studied
CMS 8	His375Tyr	Selcen *et al.* 2014	-	-	-	-	-	-	-	Not yet studied

Patient	Mutation	Reference	protein yield (mg/L)	Activity	Thermostability ($Tm_{1/2}$)					Comment
					Apo	+DolP	+UDPGlcNAc	+UMP	+ TUN	
CDG1j 1	Ile29Phe	Iqbal *et al.* 2013	0	-	-	-	-	-	-	Unable to purify protein
CDG1j 1	Leu168Pro	Iqbal *et al.* 2013	0.15±0.05	21.6±7.1	50.5±0.5	52.5±0.3	53.8±1.1	50.4±0.6	84.0±0.2	22% WT activity
CDG1j 2	Unidentified splicing Mutation	Wu *et al.*, 2003	-	-	-	-	-	-	-	No protein produced
CDG1j 2	Tyr170Cys	Wu *et al.*, 2003	0.32±0.22	20.0±15.4	50.9±0.6	54.0±1.0	54.0±0.8	50.7±0.3	83.1±0.8	20% WT activity
CDG1j 3	Arg301His	Imtiaz *et al.*, 2012	0.24±0.014	7.7±8.5	51.5±1.0	56.2±1.2	52.4±1.0	51.4±0.9	81.2±0.5	8% WT activity
CDG1j 4	Arg301Cys	Carrera *et al.*, 2012	0.24±0.20	5.5±1.7	51.1±0.7	56.4±0.5	52.5±0.6	51.4±0.8	80.9±0.5	6% WT activity
CDG1j 4	Leu385Arg	Carrera *et al.*, 2012	0.12±0.07	113.2±8.1	52.3±0.3	-	-	-	-	Activity close to WT
CDG1j 5	Splice site mutation, no mRNA made	Timal *et al.*, 2012	-	-	-	-	-	-	-	Abnormal splicing
CDG1j 5	Ile69Asn	Timal *et al.*, 2012	0.25±0.19	85.6±10.7	52.2±0.5	-	-	-	-	Activity close to WT
CDG1j 6	Ala114Gly	Wurde *et al.*, 2012	0.44±0.18	79.3±9.3	52.1±0.7	-	-	-	-	Activity close to WT, Abnormal splicing
CDG1j 7	Arg301His	Yuste-Checa *et al.*, 2017	0.24±0.014	7.7±8.5	51.5±1.0	56.2±1.2	52.4±1.0	51.4±0.9	81.2±0.5	8% WT activity
CDG1j 7	Phe110Ser	Yuste-Checa *et al.*, 2017	-	-	-	-	-	-	-	Not yet studied

Table S2: Data collection, phasing and refinement statistics. Related to Figures 1 and 2.

	**WT**	**Val264Gly**	**Val264Gly-UDP-GlcNAc**	**Val264Gly-tunicamycin**
**PDB Code**	6FM9	5LEV	6FWZ	5O5E
**Data Collection**				
Beamline	I04-1	I24	I24	I24
Space group	*P*6₅22	*P*6₅22	*P*6₅22	*P*6₅22
Crystallisation conditions	0.05M ADA pH 6.5, 24% PEG400	0.1M bicine pH 9.0, 0.05M NaCl, 38% PEG300	0.1M bicine pH 9.0, 0.05M NaCl, 37.5% PEG300	0.1M bicine pH 8.5, 0.05M NaCl, 36% PEG200
Cell dimensions				
a/*b*, *c* (Å)	103.8, 241.1	103.25, 239.15	102.46, 238.21	102.09, 240.06
α, β, γ (°)	90, 90, 120	90, 90, 120	90, 90, 120	90, 90, 120
Resolution [Å][1]	3.6 (3.60-3.69)[1]	3.2 (3.20-3.28)[1]	3.1 (3.10-3.18)[1]	3.4 (3.40-3.49)[1]
Resolution limits [Å][2]	3.84, 3.6	3.47, 3.20	3.49, 3.1	3.67, 3.4
	(3.66, 3.6)	(3.36, 3.2)	(3.37,3.1)	(3.54,3.4)
Nominal Resolution [Å][3]	3.76	3.36	3.35	3.58
$CC_{1/2}$	0.999 (0.666)	0.999 (0.549)	0.999 (0.387)	0.999 (0.435)
R_{meas}	0.061 (1.23)[1]	0.088 (2.453)[1]	0.101 (2.505)[1]	0.095 (2.419)[1]
$I / \sigma I$	17.8 (1.8)[1]	18.2 (1.4)[1]	14.4 (1.1)[1]	13.4 (1.2)[1]
Completeness [%]	99.6 (100)[1]	100.0 (100)[1]	100 (100)[1]	99.9 (99.8)[1]
Redundancy	6.2 (6.6)[1]	7.0 (7.1)[1]	9.5 (9.9)[1]	9.3 (9.8)[1]
**Refinement**				
Resolution (Å)	30 – 3.60	26.7 – 3.2	30 – 3.10	25.87 – 3.40
No. reflections (free)	9435 (479)	12893 (665)	13395 (728)	10786 (552)
R_{work} / R_{free}	24.90 / 27.0	23.4 / 24	22.26 / 23.62	22.90 / 23.60
No. atoms				
Protein	2840	2846	2892	2937
Other	25	36	106	113
B-factors (Å^2)				
Protein	171	143	136	173
Ligand	-	-	106	145
Other	184	130	140	171
R.m.s. deviations				
Bond lengths (Å)	0.009	0.009	0.012	0.009
Bond angles (°)	0.93	0.99	1.554	1.00

[1] Values in parentheses are statistics for highest resolution shell
[2] Anisotropic resolution limits along each of the three principal directions as defined by AIMLESS based on Mn (I/sd(I)) > 2. Values in parentheses are resolution limits in each direction based on half dataset correlation > 0.5 ($CC_{1/2}$).
[3] Nominal resolution is defined based on overall Mn (I/sd(I)) > 2 as estimated by AIMLESS.

Table S3. Anti-microbial susceptibility values of tunicamycin and the TUN-X,X analogues against various bacterial strains. Data presented are means ± SEM (n=3). Related to Figure 5.

MIC (µg/mL)								
	**Tunicamycin**	**TUN-cit,cit**	**TUN-7,7**	**TUN-8,8**	**TUN-9,9**	**TUN-10,10**	**TUN-11,11**	**TUN-12,12**
B. subtilis	0.0018 ±0.0011	5.2 ±1.8	5.2 ±1.8	0.65 ±0.22	0.02 ±0.01	0.33 ±0.11	1.3 ±0.45	83 ±29
M. luteus	-	-	-	-	-	-	-	-
B. cereus	0.17 ±0.06	83 ±29	83 ±29	5.2 ±1.8	0.65 ±0.23	0.33 ±0.11	0.65 ±0.23	42 ±14
S. aureus	42 ±14	>400	170 ±60	170 ±60	83 ±29	83 ±29	170 ±60	170 ±60
P. aeruginosa	>400	170 ±60	83 ±29	83 ±29	83 ±29	83 ±29	83 ±29	170 ±6
E. coli	>400	>400	330 ±120	330 ±120	330 ±120	330 ±120	330 ±120	330 ±120

MBC (µg/mL)								
	**Tunicamycin**	**TUN-cit,cit**	**TUN-7,7**	**TUN-8,8**	**TUN-9,9**	**TUN-10,10**	**TUN-11,11**	**TUN-12,12**
B. subtilis	>0.00.12	>200	>50	>0.78	>0.05	>0.39	>1.56	>200
M. luteus	-	-	-	-	-	-	-	-
B. cereus	25	>200	>200	>50	>25	12.5	>25	>200
S. aureus	>200	>200	>400	400	400	400	400	>400
P. aeruginosa	>400	>400	>400	>400	400	400	400	>400
E. coli	>400	400	>400	400	400	400	400	>400

IC_{50} (µg/mL)								
	**Tunicamycin**	**TUN-cit,cit**	**TUN-7,7**	**TUN-8,8**	**TUN-9,9**	**TUN-10,10**	**TUN-11,11**	**TUN-12,12**
B. subtilis	0.00018 ±0.00006	0.36 ±0.12	3.12 ±1.04	0.20 ± 0.07	0.015 ±0.005	0.12 ±0.04	0.85 ±0.28	58.92 ±19.64
M. luteus	-	-	-	-	-	-	-	-
B. cereus	0.0029 ±0.0010	6.29 ±2.10	13.33 ±4.45	0.78 ±0.26	0.072 ±0.024	0.046 ±0.015	0.088 ±0.029	1.40 ±0.47
S. aureus	20.78 ±6.93	176.70 ±58.90	80.49 ±26.83	58.36 ±19.45	48.92 ±16.31	49.29 ±16.43	58.03 ±19.34	61.88 ±20.63
P. aeruginosa	>400	70.29 ±23.43	62.44 ±20.81	46.57 ±15.52	54.98 ±18.32	53.46 ±17.82	63.17 ±21.06	81.74 ±27.31
E. coli	>400	217.90 ±72.63	131.20 ±43.73	122.70 ±40.90	87.06 ±29.02	91.63 ±30.54	131.10 ±43.70	181.60 ±60.53

Table S4. *Mtb* MIC values with average ± Std. Dev. Data presented are means ± SEM (n=3). Related to Figure 5.

Compound	***1-week MIC in 7H9/ADC/Tw (ug/mL)***	***1-week MIC in GAST/Fe (ug/mL)***
MilliQ water	No inhibition	No inhibition
Methanol	No inhibition	No inhibition
Tunicamycin	0.6 ± 0.2	0.08 ± 0.06
**(2)**	>60	42.5 ± 3.5
**TUN-Ac,Ac**	>=60	21.3 ± 1.8
**TUN**	>60	>60
**TUN-Boc,Boc**	>60	>60
**TUN-Cit,Cit**	16.3 ± 8.8	3.4 ± 3.1
**TUN-7,7**	5.0 ± 3.5	1.6 ± 1.8
**TUN-8,8**	0.7 ±3.3	0.14 ± 0.1
**TUN-9,9**	0.2 ± 0.02	0.03 ± 0.001
**TUN-10,10**	1.2 ± 0.3	0.1 ± 0.1
**TUN-11,11**	4.1 ± 2.2	1.6 ±1.6
**TUN-12,12**	18.7 ± 5.4	6.9 ± 6.2

Table S5. Assessing toxicity of TUN and the analogues in HEK293, HepG2 and Raji cells. LD_{50} calculated from cells cultured in liquid media. Related to Figure 6.

Compound	LD_{50} values (μg mL^{-1}), Avg. ± Std. Dev.		
	**HEK293**	**HepG2**	**Raji**
MilliQ Water	N/A	N/A	N/A
Methanol	N/A	N/A	N/A
**Tunicamycin**	51.25 ±31.27	44.74 ±4.73	26.82 ±11.46
**(2)**	N/A	N/A	303.80 ±9.83
**TUN-Ac,Ac**	N/A	N/A	212.30 ±51.48
**TUN-Boc,Boc**	N/A	N/A	608.9 ±394.9
**TUN**	N/A	N/A	698.3
**TUN-Cit,Cit**	N/A	N/A	177.75 ±75.17
**TUN-7,7**	N/A	N/A	431.10 ±241.81
**TUN-8,8**	N/A	N/A	211.05 ±125.94
**TUN-9,9**	N/A	N/A	355.57 ±194.57
**TUN-10,10**	N/A	N/A	196.17 ±47.61
**TUN-11,11**	N/A	N/A	103.65 ±34.34
**TUN-12,12**	N/A	N/A	81.29 ±30.20

The LD_{50} values are calculated from MTS cell proliferation assay based on dose response curves. The data shown are mean ± SEM errors of three independent experiments. N/A = not available. No or low cytotoxicity observed at highest, saturating concentration tested (400 μg mL^{-1}), thus a reliable dose-response curve could not be generated from the experimental data.

Table S6. Minimal Lethal Dose, Minimal Inhibitory Concentration (µg/mL) and Relative Therapeutic Index (RTI). Related to Figure 5 and 6.

	HepG2	HEK 293	*Mtb* H37RV [a]	RTI	*Mtb* H37RV [b]	RTI	*B. subtilis* EC1524	RTI	*B. cereus* NRRL 11778	RTI
Tunicamycin	100	100	0.6	167	0.08	1250	0.0015	66700	0.195	513
**TUN[c]**	>400	>400	>60		>60		100	8	100	8
**TUN-Cit,Cit**	>400	>400	16.3	49	3.4	235	6.25	128	100	8
**TUN-7,7**	>400	>400	5.0	160	1.6	500	6.25	128	100	8
**TUN-8,8**	>400	>400	0.7	1140	0.14	4714	0.78	1026	6.25	128
**TUN-9,9**	>400	>400	0.2	4000	0.03	27000	0.024	33300	0.78	1026
**TUN-10,10**	>400	>400	1.2	667	0.1	8000	0.39	2050	0.39	2050
**TUN-11,11**	>400	>400	4.1	195	1.6	500	1.56	513	0.78	1026
**TUN-12,12**	>400	>400	18.7	43	6.9	116	100	8	50	16

[a] 1-week in 7H9/ADC/Tw

[b] 1-week in GAST/Fe

[c] as *bis*-hydrochloride salt

Relative therapeutic index (RTI) is the ratio between the minimal lethal dose and the minimal inhibition concentration. The micro-broth dilution and the cytotoxicity tests were carried out by serial 2-fold dilutions. For no detectable cytotoxicity at 400 µg mL^{-1} (>400), a minimal lethal dose of 800 µg mL^{-1} was used to calculate the RTI.

Table S7. Two series of TUN-8,8 pharmacokinetic studies in mouse blood. Data are mean ± SD (n=5). Related to Figure 7.

Time (h)	Concentration (μM)	Relative error	Concentration (μM)	Relative error
0.5	42.66±28.33	66%	52.096±34.47	66%
1	34.63±7.4	21%	42.29±9.04	21%
2	23.66±9.25	39%	28.89±11.29	39%
4	1.32±1.09	83%	1.61±1.33	83%
6	0.21±0.12	57%	0.26±0.15	57%
24	0.06±0.08	147%	0.07±0.1	147%

Table S8. Oligonucleotides used to clone DPAGT1 and mutagenesis. All oligonucleotides purchased from Eurofins Genomics. Related to Key Resources Table and Figure 4.

**Mutation**	**Primer sequence**
pFB-LIC-Bse-DPAGT1 Template Forward	TACTTCCAATCCATGTGGGCCTTCTCGGAATTGC
pFB-LIC-Bse-DPAGT1 Template Reverse:	TATCCACCTTTACTGTCAGACATCATAGAAGAGTCGAACG
DPAGT1 WT Forward:	TACTTCCAATCCATGTGGGCCTTCTCGGAATTGC
DPAGT1 WT Reverse	TATCCACCTTTACTGTCAGACATCATAGAAGAGTCGAACG
Pro30Ser	CAGTCACCCTCATCTCGGCCTTCCGGGGCC
Ile69Asn	F: GTTTTCCTTATCAACCTCTTCTGCTTC<br>R: GAAGCAGAAGAGGTTGATAAGGAAAAC
Leu103Phe	CATGCAGCAGATGGCaaaGAGGGCACCTATCAG
Met108Ile	CATCTGCTGCATCATCTTCCTGGGCTTTGCG
Phe110Ala	CCGCAAAGCCCAGtgcGATCATGCAGCAG
Ala114Gly	F: TTCCTGGGCTTTGGCGATGATGTACTG<br>R: CAGTACATCATCGCCAAAGCCCAGGAA
Asp115Asn	GATTCAGTACATCgttCGCAAAGCCCAGG
Asp115Glu	GATTCAGTACATCttcCGCAAAGCCCAGG
Asp115Ala	GATTCAGTACATCggcCGCAAAGCCCAGG
Asp116Asn	GCAGATTCAGTACattATCCGCAAAGCCC
Asp116Ala	CAGATTCAGTACggcATCCGCAAAGCC
Val117Ile	GCCAGCGCAGATTCAGTATATCATCCGCAAA
Leu120Met	GATGTACTGAATATGCGCTGGCGCCATAAGC
Trp122Ala	CAGCTTATGGCGtgcGCGCAGATTCAG
Lys125Asn	GCTGTAGGTAGCAGCAGattATGGCGCCAGCGCAGAT
Lys125Glu	GCTGTAGGTAGCAGCAGttcATGGCGCCAGCGCAGAT
Lys125Ala	GCTGTAGGTAGCAGCAGcgcATGGCGCCAGCGCAGAT
Leu168Pro	CATGTAGACATAGTAaggGATTCCCAAGTCCAG
Tyr170Cys	F: GGAATCCTGTACTGCGTCTACATGGGG<br>R: CCCCATGTAGACGCAGTACAGGATTCC
Asn182Asp	GGATATTGATGGCatcGGTACAGAACAC
Asn182Ala	GATATTGATGGCtgcGGTACAGAACAC
Asn185Asp	TTAATTCCTGCTAGGATgtcGATGGCATTGGTACAGA
Asn185Ala	TTAATTCCTGCTAGGATggcGATGGCATTGGTACAGA
Gly192Ser	CTAGCAGGAATTAACAGCCTAGAGGCTGGC
Asp252Asn	CACGGGTGTTTGTGGGAaatACCTTCTGTTACTTTGC
Asp252Ala	GTGTTTGTGGGAgccACCTTCTGTTAC
Val264Gly	GCATGACCTTTGCCGGGGTGGGCATCTTGGG
Val264Met	CATGACCTTTGCCatgGTGGGCATCTTGG
Arg301His	F: ATCCCCTGCCCTCATCACCGCATACCC<br>R: GGGTATGCGGTGATGAGGGCAGGGGAT

Arg301Cys	F: ATCCCCTGCCCTTGCCACCGCATACCC<br>R: GGGTATGCGGTGGCAAGGGCAGGGGAT
His302Ala	CCCCTGCCCTCGCgcaCGCATACCCAGAC
Arg303Ala	TCCCCTGCCCTCGCCACgcgATACCCAGACTCAATAT
Leu385Arg	F: TTGCTCCTGCTGCGCCTGCAGATCCTG<br>R: CAGGATCTGCAGGCGCAGCAGGAGCAA
